# Probing the influence of imidazolylidene- and triazolylidene-based carbenes on the catalytic potential of dioxomolybdenum and dioxotungsten complexes in deoxygenation catalysis†

**DOI:** 10.1039/d4qi02392g

**Published:** 2024-12-24

**Authors:** Florian R. Neururer, Florian Heim, Marc Baltrun, Philipp Boos, Julia Beerhues, Michael Seidl, Stephan Hohloch

**Affiliations:** a University of Innsbruck, Department of General, Inorganic and Theoretical Chemistry Innrain 80–82 6020 Innsbruck Austria Stephan.Hohloch@uibk.ac.at; b University of Paderborn, Department of Chemistry Warburger Straße 100 33098 Paderborn Germany; c Freie Universität Berlin, Department of Inorganic Chemistry Fabeckstraße 34–36 14195 Berlin Germany

## Abstract

We report the synthesis of dianionic OCO-supported NHC and MIC complexes of molybdenum and tungsten with the general formula (OCO)MO_2_ (OCO = bis-phenolate benzimidazolylidene M = Mo (1-Mo), bis-phenolate triazolylidene M = Mo (2-Mo), M = W (2-W) and bis-phenolate imidazolylidene, M = Mo (3-Mo), W (3-W)). These complexes are tested in the catalytic deoxygenation of nitroarenes using pinacol as a sacrificial oxygen atom acceptor/reducing agent to examine the influence of the carbene and the metal centre in this transformation. The results show that the molybdenum-based triazolylidene complex 2-Mo is by far the most active catalyst, and TOFs of up to 270 h^−1^ are observed, while the tungsten analogues are basically inactive. Mechanistic studies suggest that the superiority of the triazolylidene-based complex 2-Mo is a result of a highly stable metal carbene bond, strongly exceeding the stability of the other NHC complexes 1-Mo and 3-Mo. This is proven by the structural isolation of a triazolylidene pinacolate complex (5-Mo) that can be thermally converted to a μ-oxodimolybdenum(V) complex 7-Mo. The latter complex is very oxophilic and stoichiometrically deoxygenates nitro- and nitrosoarenes at room temperature. In contrast, azoarenes are not reductively cleaved by 7-Mo, suggesting direct deoxygenation of the nitroarenes to the corresponding anilines with nitrosoarenes as intermediates. In summary, this work showcases the superior influence of MIC donors on the catalytic properties of early transition metal complexes.

## Abstract 2

We report the synthesis of molydenum and tungsten NHC and MIC complexes with the general formula (OCO)MO_2 _(OCO = bis-phenolate NHC/MIC; M = Mo, W) and their utility in the catalytic deoxygenation of nitroarenes.

## Graphical abstract

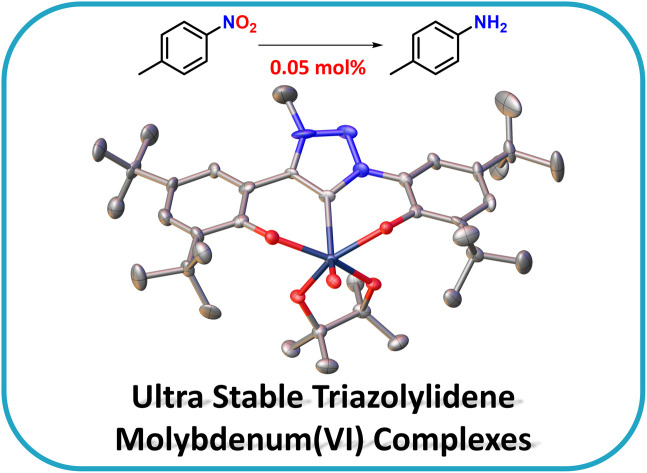

## Introduction

The creation of sustainable and environmentally benign chemical processes is one of the most important tasks of our time.^1,2^ In this context, the development of resource-saving, environmentally friendly, and efficient catalytic reactions is a crucial task to realise this goal. Given the current transition from fossil fuels to environmentally favourable carbon sources, such as biomass,^1,2^ or more generally, oxidatively over-functionalized materials, the need for their efficient reduction to industrially compatible starting materials is of growing importance.

Targeting this goal, a plethora of strategies have been introduced in the past decades,^3–5^ which include the use of either molecular hydrogen^6–8^ or hydrogen surrogates *e.g.*, in transfer-hydrogenation processes.^9–14^ However, both strategies rely on expensive transition metal catalysts,^5,10,11,15–17^ are relatively unselective towards functional groups,^18–22^ and (for direct hydrogenations) pressurized reaction vessels (*e.g.* 50 bar H_2_ pressure) are needed.^23^ Furthermore, transfer hydrogenation requires a large excess of the hydrogen surrogate reagent (*e.g.*, isopropanol, silanes or boranes) producing vast amounts of chemical waste and complicated waste remediation strategies.^17,24–27^

**Fig. 1:**
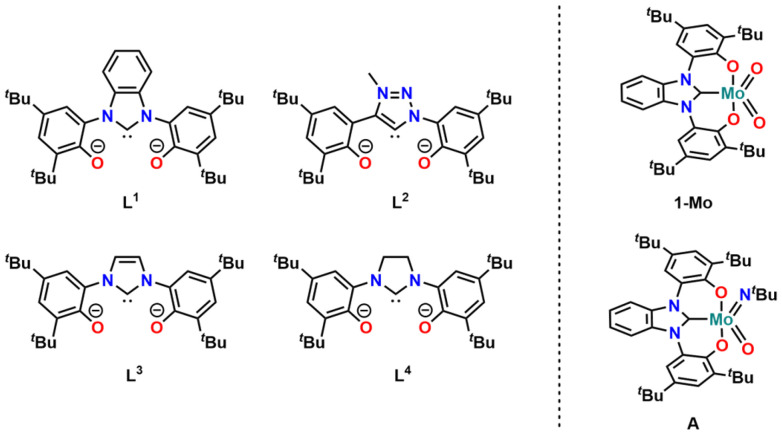
Overview of OCO chelating ligands L^1^–L^4^ used in this work (left) and previously reported NHC molybdenum deoxygenation catalysts 1-Mo and A reported by our group (right).

Overcoming many of these drawbacks, oxygen atom transfer reactions (OATR) have proven to be a useful alternative: they are very atom efficient,^28–33^ do not need pressurized reaction vessels, and most catalysts used in this transformation are based on early transition metals,^34–36^ primarily molybdenum^37–39^ and tungsten.^40–45^ Furthermore, they only “focus” on oxygen-containing groups, leaving other functional groups (alkenes, alkynes, *etc*.) unchanged.^4,46,47^ However, one major drawback in this reaction so far was the need for quite high catalyst loadings of 5–10 mol%^48,49^ for the catalytic deoxygenation of phosphine oxides, sulfoxides, or nitroarenes using simple MoO_2_Cl_2_L_2_ (L = DMF, bpy, *etc*.) catalysts.^46,47,50–54^ Studying the reduction of nitroarenes, we have found that the use of OCO-chelating N-heterocyclic carbenes^55–67^ with a benzimidazolylidene donor^68,69^ (1-Mo, Fig. 1) has a significant effect on the efficiency of the catalyst and we were able to lower the catalyst loading to 0.25 mol%.^70,71^

Here, we expand this strategy to the use of other *N*-heterocyclic and mesoionic carbene donors, namely imidazolylidene,^69^ imidazolidinylidene^55^ and triazolylidene units.^56^ In the past decade, triazolylidene-based carbenes^72^ have found wide application in chemistry.^73–78^ The increased stability of their metal complexes, their modular synthesis *via* the copper,^79,80^ ruthenium,^81^ or base catalysed^82^ [3 + 2] azide-alkyne cycloaddition, and strong σ-donor properties^73,83^ have made them superior ligands in organometallic chemistry^62,84–91^ and chemical catalysis.^11,12,92–103^ The aim of this work is to investigate whether the carbene has an influence on the catalytic potential of the corresponding molybdenum complex in the deoxygenation of nitroarenes, using pinacol as a sacrificial reductant. Furthermore, we aimed at expanding this concept to tungsten-based catalysts supported by the NHC/MIC ligands L,^1–4^ as it was shown that tungsten can sometimes outperform the catalytic potential of molybdenum in OAT reactions.^40^

## Results & discussion

### Molybdenum & tungsten dioxo complexes

**Scheme 1:**
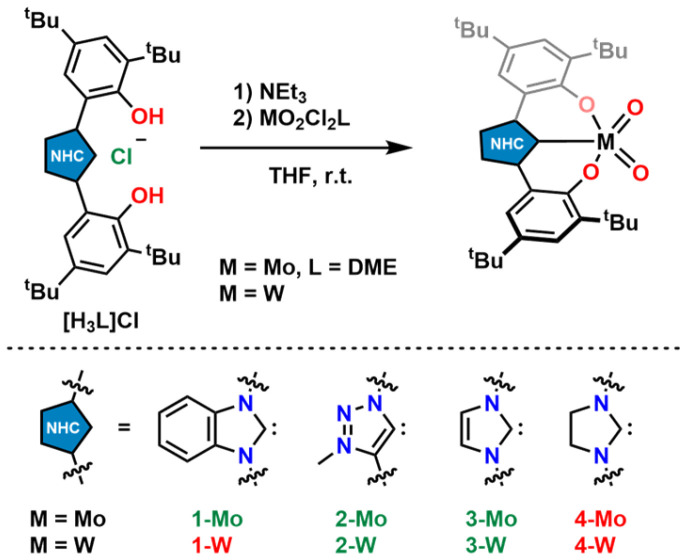
(Attempted) synthesis of an isostructural series of Mo and W complexes supported by OCO-chelating bis-phenolate NHC/MIC donors. Green indicates successful, red unsuccessful synthesis.

**Fig. 2:**
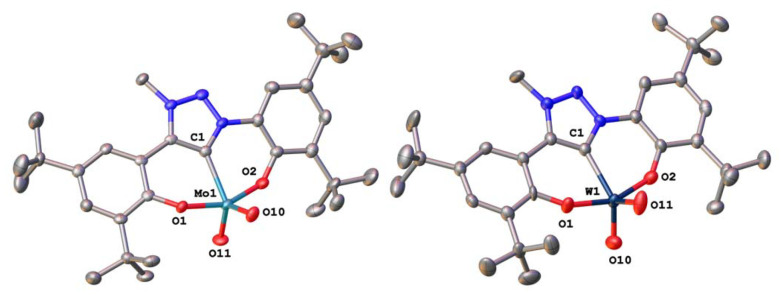
Molecular structure of the triazolylidene molybdenum complex 2-Mo (left) and its tungsten analogue 2-W (right). Hydrogen atoms and solvent lattice molecules have been omitted for clarity. Ellipsoids are shown at a probability level of 50%.

Triazolylidene supported dioxocomplexes 2-Mo and 2-W were prepared *via* an *in situ* deprotonation procedure as described for the benzimidazolylidene-supported complex 1-Mo (Scheme 1).^71^ The ligand precursor was first treated with an excess of triethylamine and then added to a solution/suspension containing the corresponding dioxo metal dichloride (MoO_2_Cl_2_(dme)^104^ or WO_2_Cl_2_) in THF. After stirring the reaction overnight, subsequent removal of triethylammonium chloride and precipitation using *n*-hexane, molybdenum complex 2-Mo was isolated as a bright yellow powder (91% yield), while its tungsten analogue 2-W was obtained as an off-white solid (70% yield). Notably, the molybdenum complex 2-Mo is soluble in THF, dichloromethane, and aromatic hydrocarbons, poorly soluble in diethyl ether, and almost insoluble in aliphatic hydrocarbons. In contrast, the tungsten complex 2-W is only soluble in THF, dichloromethane, and acetonitrile, sparingly soluble in aromatic hydrocarbons, and almost insoluble in diethyl ether, and aliphatic hydrocarbons. In the ^1^H NMR spectra of both compounds, the absence of the triazolium 5-*H* and the phenolic protons is indicative of the presence of MIC complexes (Fig. S1 and S6†). ^13^C-NMR resonances corresponding to a carbene carbon atom are found at 159.3 ppm for 2-Mo (in benzene-*d*_6_, Fig. S2†) and 168.1 ppm for 2-W (in dichloromethane-*d*_2_, Fig. S7†), which is in line with previously reported early transition metal triazolylidene complexes.^56,61^ The infrared-stretching frequencies at 904 cm^−1^ and 861 cm^−1^ corresponding to the terminal molybdenum-oxo frequencies in 2-Mo are well in line with the literature (Fig. S73†).^71^ For 2-W, these frequencies are observed at 904 cm^−1^ and 861 cm^−1^ (Fig. S74†).^40^ Furthermore, single crystals suitable for X-ray diffraction analysis could be obtained for both compounds (Fig. 2). Molybdenum compound 2-Mo was crystallised by slow diffusion of *n*-pentane into a benzene-*d*_6_ solution of the complex. It crystallises in the *monoclinic* space group *P*2_1_/*c* with two complex units and one benzene-*d*_6_ molecule in the asymmetric unit. The molybdenum centre is five-coordinate, displaying a *τ*_5_ value of 0.53. With a carbon-metal bond (Mo1–C1) of 2.170(5) Å, the complex compares to other molybdenum complexes bearing a tridentate OCO ligand framework.^56,71^ The distances between the molybdenum centre and the terminal oxo ligands (Mo1–O10 and Mo1–O11) are 1.696(3) Å and 1.705(3) Å, respectively. Notably, 2-Mo can also be crystallised in the presence of triethylphosphine oxide (OPEt_3_) displaying no interaction between the phosphine oxide and the molybdenum centre (Fig. S86†), contrasting the complexation and coordination behaviour of 1-Mo in the presence of OPEt_3_.^70^ Interestingly, also in solution, no interactions between OPEt_3_ and 2-Mo could be observed (Fig. S62†). Single crystals of 2-W were grown from a concentrated acetonitrile solution at −40 °C. This compound crystallises in the *monoclinic* space group *C*2/*c* with four complex units and four solvent molecules in the asymmetric unit. Like in 2-Mo, the metal centre in 2-W is five-fold coordinated by the ligand and two terminal oxo ligands, displaying a *τ*_5_ value of 0.46. The bonding distances differ in each of the four complex units. For instance, the carbon-metal distances vary between 2.172(6) Å and 2.208(7) Å and are similar to previously reported tungsten(0) triazolylidene complexes.^105,106^ The slight variation in the C1–W1 bond distance is attributed to packing effects. The distance between the metal centre and the terminal oxo ligands (W1–O10 and W1–O11) are 1.714(5) Å and 1.714(5) Å, respectively. Confirming our previous results,^71^ both complexes are stable against air and moisture and dry samples or solutions may be kept under air at room temperature.

**Fig. 3:**
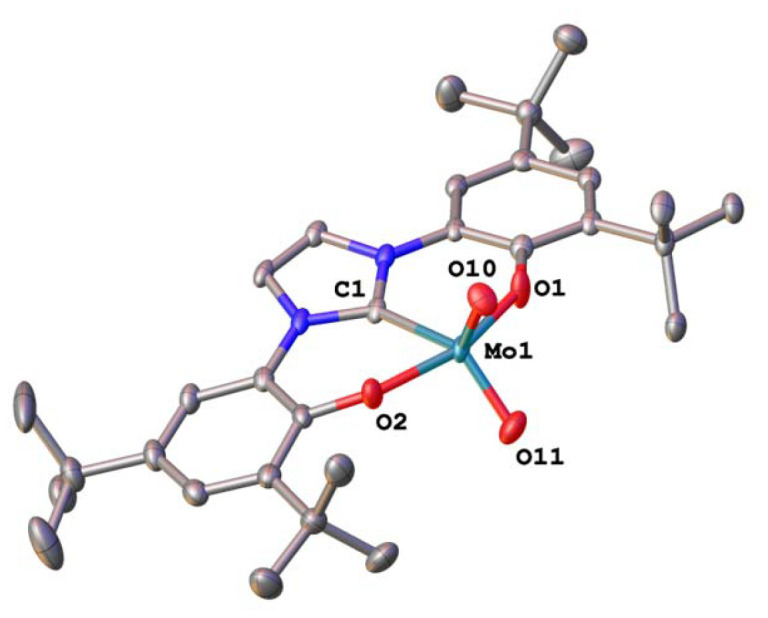
Molecular structure of the triazolylidene molybdenum complex 3-Mo. Hydrogen atoms and solvent lattice molecules have been omitted for clarity. Ellipsoids are shown at a probability level of 50%.

Having the triazolylidene complexes 2-M (M = Mo, W) and the molybdenum benzimidazolylidene complex 1-Mo previously reported by us^70,71^ in hand, we aimed for the isolation of the imidazolylidene based complexes 3-Mo and 3-W, as well as the tungsten benzimidazolylidene complex 1-W, to complete the series. 3-Mo was prepared following a procedure analogous to 1-Mo and 2-Mo (Scheme 1). The ^1^H-NMR spectrum shows the expected signature with absent phenolic OH and imidazolium 2-*H* protons (Fig. S11†). A resonance in the ^13^C{^1^H}-NMR spectrum at 171.9 ppm can be assigned to the carbene carbon atom (Fig. S12†). In comparison with 2-Mo (159.3 ppm), the carbene carbon resonance is significantly shifted downfield, but upfield from 1-Mo (183.1 ppm).^71^ This indicates that the imidazole-containing ligand is a weaker donor than the phenolate-tethered triazolylidene but stronger than the benzimidazolylidene ligand. X-ray quality single crystals were grown from tetrahydrofuran/hexane mixtures at low temperatures (Fig. 3). Apart from the Mo1–C1 distance, which was found at 2.225(3) Å in 3-Mo and is thus slightly longer compared to 2-Mo (2.170(5) Å) and 1-Mo (2.193(4) Å),^71^ the remaining structural parameters of 3-Mo strongly resemble those of complex 1-Mo and 2-Mo, wherefore a detailed discussion will be omitted here (see ESI for further information, Tables S1 and S2†). The synthesis of the tungsten analogues 1-W and 3-W, however, turned out to be more challenging. Unfortunately, neither the general procedure^71^ nor any other protocol (varying solvent, base, and temperature) yielded an analogous benzimidazolylidene complex 1-W, and only intractable mixtures could be obtained. In contrast, the imidazolylidene complex 3-W can be accessed following the general procedure, however, in poor yields only (18%, not optimized). Successful synthesis of 3-W is deduced from the ^1^H NMR spectrum, showing the presence of four resonances and the absence of the characteristic imidazole 2-*H* proton (Fig. S16†). Furthermore, the ^13^C NMR shows the distinctive downfield resonance at 181.1 ppm, which is characteristic for an NHC complex (*vide supra* and Fig. S17†). Finally, crystals suitable for X-ray diffraction analysis could be grown from a concentrated solution of 3-W in *n*-hexane at room temperature. Although these crystals were of poor quality and strongly twinned, the model confirms that 3-W is an NHC complex (Fig. S85†). Interestingly, the asymmetric unit contains two different conformers of 3-W: the expected monomeric form of 3-W_mono_ and a trimeric form 3-W_trimer_ in which three tungsten atoms are bridged by μ-oxo units, depending on the ligand conformation (Fig. S85†). A comparable effect has already been found with 1-Mo, which was observed as a monomer and a dimer in the solid state.^71^ Overall, each tungsten atom is coordinated by two oxo ligands and one ligand L^3^, unambiguously confirming the assignment of the +VI oxidation state for each tungsten atom. Due to the low quality of the structure, no bond distances can be discussed here. Finally, all attempts to isolate an imidazolidine-2-yldidene complex (putative 4-Mo and 4-W, Scheme 1) have failed so far for both molybdenum and tungsten. We thus conclude that only electron-rich NHC ligands are compatible with the strongly Lewis-acidic MO_2_ (M = Mo, W) frameworks.

### Catalytic deoxygenation of nitroarenes

**Fig. 4:**
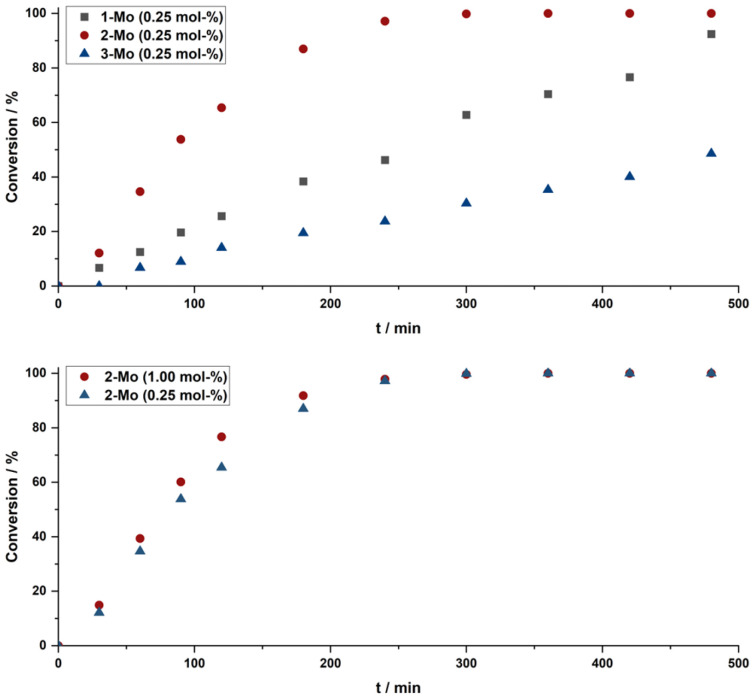
Time-Conversion plots of the catalytic deoxygenation of *o*-nitrotoluene in toluene using pinacol as a sacrificial reducing agent. Conversion was determined *via* GC-MS analysis of aliquots of the reaction mixtures taken after the corresponding times, using mesitylene as an internal standard. Top: 0.25 mol% of the respective catalysts (blue triangles: 3-Mo; red dots 2-Mo and grey squares 1-Mo). Bottom: different catalyst loadings of 2-Mo (red dots: 1.00 mol%, blue triangles: 0.25 mol%).

Since dioxomolybdenum and dioxotungsten compounds are known to efficiently catalyse deoxygenation reactions,^4,40,46,50,54^*e.g.* the deoxygenation of sulfoxides, *N*-oxides, or the reduction of nitroaromatic compounds to the corresponding anilines,^4,46,50–53^ the catalytic potential of 2-Mo was investigated for nitroarene reductions. As a model reaction for initial studies, the conversion of *o*-nitrotoluene to *o*-toluidine using pinacol as a sacrificial reducing agent was chosen. To test the general activity of complex 2-Mo, the deoxygenation reaction was conducted in anhydrous toluene at 130 °C, and four equivalents of pinacol and 1.00 mol% of 2-Mo were applied. After 6 hours, an aliquot was removed from the reaction and analysed *via* gas chromatography, which confirmed that the reaction was complete. Given the fact that the analogous benzimidazolylidene complex 1-Mo allowed catalyst loadings as low as 0.25 mol%,^70^ we aimed at reducing the catalyst loading of 2-Mo as well. Catalyst loadings as low as 0.25 mol% resulted in complete conversion within 6 hours. (Table 1, entry 2) Furthermore, time-dependent experiments have shown that applying 0.25 mol% is almost as efficient as applying 1.00 mol%, indicating that the reaction does not benefit from increasing the catalyst loading (Fig. 4, bottom). Further decrease of the catalyst loading of 2-Mo resulted in lower conversions. However, at loadings of 0.1 mol%, still 94% conversion is achieved within 6 hours (Table 1, entry 3). Further reduction of the catalyst loading to 0.05 mol% shows 79% conversion after 6 hours (Table 1, entry 4). Translated into turnover numbers (TON) and turnover frequencies (TOF): at 0.05 mol%, a TON of 1600 and a TOF of approx. 270 h^−1^ is achieved with 2-Mo as a catalyst. To set this into relation, initial reports by Sanz *et al.* have used 5 mol% catalyst loadings, resulting in a TON of 20 and TOF of 9 h^−1^.^54^ Compared to 1-Mo, which has shown a TON of 280 and a TOF of 47 h^−1^ at 0.25 mol% after 6 hours, the triazolylidene complex 2-Mo exhibits increased activity. To set the activity of 2-Mo into further relation with other metals, Grieco and Blacque recently reported the use of (IMes)_2_Re^V^OBr_3_ using phenylsilane as a sacrificial reductant in a microwave-assisted reaction. This was performed at 0.5 mol% of catalyst and only nitrobenzene could be converted, while functionalized nitroarenes could not be reduced.^107^ Thus, we can confidently state, that the triazolylidene complex 2-Mo is by far the most active group VI NHC/MIC-based catalyst in the deoxygenation of nitroarenes reported so far.

**Table 1:** Conditions screening for nitroarene deoxygenation catalysis using NHC/MIC supported molybdenum and tungsten complexes with pinacol as a sacrificial reductant^a^

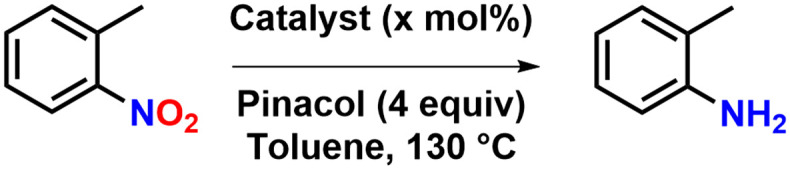				
Entry	Cat.	Loading mol%	Conv./%	TON/TOF d h^−1^
1	2-Mo	1.00	>99^b^	100/17
2	2-Mo	0.25	>99^b^	400/100^e^
3	2-Mo	0.10	94^b^	940/160^f^
4	2-Mo	0.05	79^b^/84^c^	1600/270^f^
5	1-Mo	0.25	70^b^/93^c^	280/47
6	3-Mo	0.25	35^b^/49^c^	140/35
7	2-W	1.00	0^c^	—
8	3-W	1.00	0^c^	—

a Reactions were carried out in 10 ml J-Young tubes, using 1 mmol of substrate, 4 mmol of pinacol and the indicated amount of catalyst in 3 ml toluene. Conversions were determined by GC using mesitylene as an internal standard. Each reaction was performed three times to achieve redundancy of the values.

b Conversion after 6 hours.

c Conversion after 8 hours.

d TOF values correspond to a reaction time of 6 h.

e conversion is measured after 4 hours.

f TON/TOF values are rounded to two significant digits.

To further study the influence of the NHC donor group, time-dependent catalysis was performed with 0.25 mol% catalyst loading, applying the molybdenum dioxo complex 1-Mo, 2-Mo, and 3-Mo (Fig. 4, top), respectively. As already indicated by the comparison between 1-Mo and 2-Mo (*vide supra*), the ligand has a major impact on the catalytic activity in the model reaction. Under these specified conditions (0.25 mol% catalyst loading), as expected, complex 2-Mo with the mesoionic triazolylidene donor was found to be the most active catalyst and full conversion was observed within 4 hours (TON 400, TOF 100 h^−1^). In contrast, the imidazolylidene complex 3-Mo was found to be the least active and only 35% conversion was observed over the course of 6 hours/49% over the course of 8 h (TON 140/200; TOF 35/25 h^−1^). The decrease of the TOF in 3-Mo after 6 compared to 8 hours of reaction time further indicates a limited stability of the active species in solution for 3-Mo (*vide infra*). The performance of the benzimidazolylidene complex 1-Mo, was found to lie in between 2-Mo and 3-Mo, showing conversion of 70% after 6 and 93% after 8 hours (TON 280/370 TOF 47/47 h^−1^). Finally, we were interested in how the tungsten complexes 2-W and 3-W would compete in the reaction. However, the tungsten analogues did not show any activity, even after prolonged reaction times or higher catalyst loadings of 1 mol% (Table 1, entries 7 and 8).

**Scheme 2:**
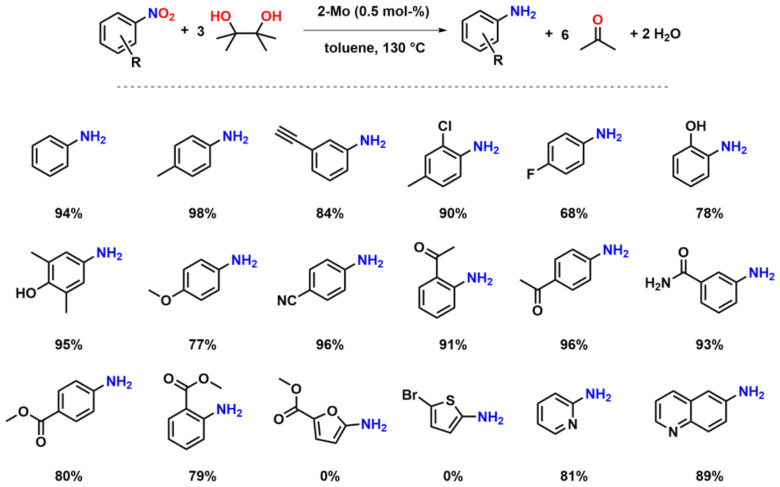
Substrate scope. Yields given correspond to isolated yields (see ESI† for further information on workup). Reactions were carried out in 10 ml J-Young tubes, using 1 mmol of substrate, 4 mmol of pinacol and the indicated amount of catalyst in 3 ml toluene.

Since *o*-nitrotoluene is efficiently converted to *o*-toluidine, we were finally interested in the scope of the triazolylidene complex 2-Mo and whether easy-identifiable substrate limitations would exist (Scheme 2). To ensure full conversion, all further nitro-reduction reactions were conducted with 0.50 mol% of 2-Mo and four equivalents of pinacol in anhydrous toluene. All yields reported correspond to isolated yields. The scope shows that complex 2-Mo is a valuable catalyst, transforming protic (phenols), halogenated, nitriles, amides, alkyne and keto-functionalized nitroarenes efficiently to the corresponding anilines. We furthermore studied the scope of functionalized heterocycles. This shows, that complex 2-Mo efficiently deoxygenates nitropyridines and quinolines, but no product formation is observed if more strained substrates such as differently substituted furans and thiophenes are used. It is unclear, if the catalyst fails using these substrates, or if the substrates do not tolerate the conditions required. In the case of thiophenes fast precipitation of insoluble, black solids is observed, indicating potential polymerisation of the thiophene units. Notably, the low isolated yield of 68% of 4-fluoroaniline most likely results from mechanical losses during column chromatography, since crude NMR spectra recorded after the catalytic transformation indicate 92% product formation (Fig. S32†).

### Mechanistic investigations for the molybdenum mediated process

**Scheme 3:**
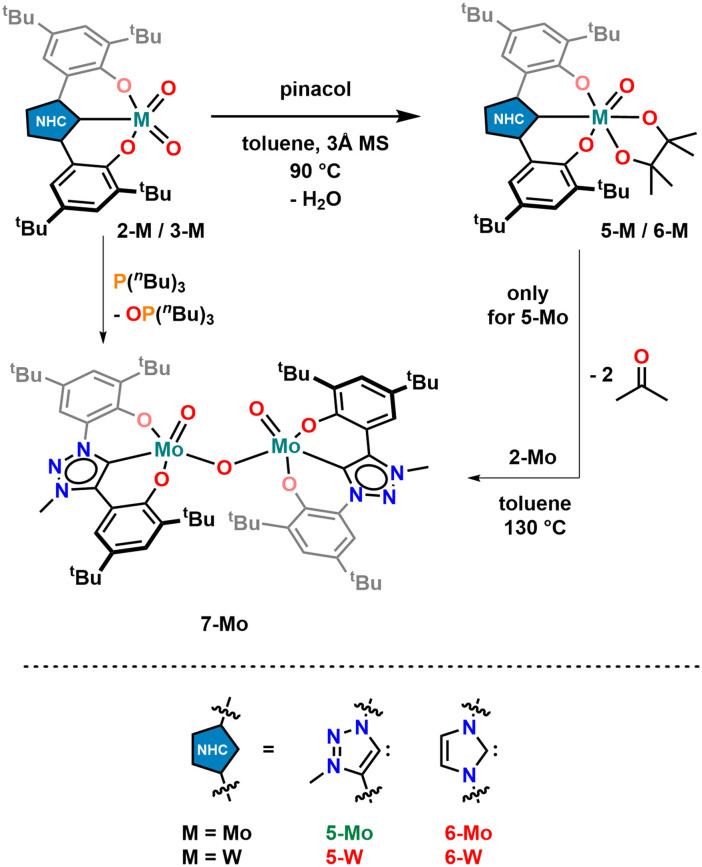
Reaction of dioxomolybdenum complexes 2-Mo/3-Mo with pinacol to give pinacolate complex 5-Mo/6-Mo and subsequent comproportionation reaction of 5-Mo and 2-Mo to the μ-oxo bridged dimer 7-Mo. Green labels indicate successful, red labels unsuccessful synthesis.

**Fig. 5:**
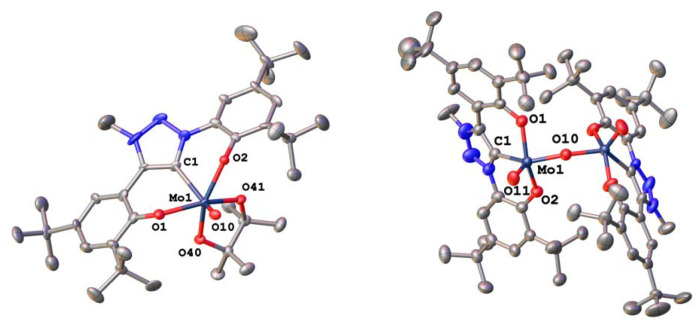
Molecular structures of the molybdenum(vi) pinacolate complex 5-Mo (left) and the reduced μ-oxo dimolybdenum(v) complex 7-Mo (right). Hydrogen atoms and solvent lattice molecules have been omitted for clarity. Ellipsoids are shown at a probability level of 50%.

To examine the activity differences observed in the series 1-Mo, 2-Mo, 3-Mo, 2-W and 3-W, further mechanistic studies were executed. First, we examined the stability of the complexes towards pinacol, which serves as a sacrificial reducing agent. Previous results have shown that the benzimidazolylidene complex 1-Mo is not stable towards pinacol, resulting in the partial protonation of the benzimidazolylidene ligand.^70^ To examine whether an analogous, partially hydrolysed intermediate is possible with 2-Mo and 3-Mo both complexes were mixed with pinacol (2 equiv.) in benzene-*d*_6_ (Scheme 3). However, after a few hours at room temperature, no new resonances were observed in the ^1^H-NMR spectrum (Fig. S63 and S65†). When these mixtures are heated to 70 °C, they take on a blood red colour but turn bright yellow again when cooled to room temperature, indicating a strongly dynamic reaction/equilibrium. Notably, this “switching” can be done for 2-Mo several times without the indication of any complex decomposition (Fig. S64†), while for 3-Mo severe signs of multiple decomposition products are already observed within the first cycle (Fig. S66†). This indicates that similar to 1-Mo, the imidazolylidene complex 3-Mo is not stable under protic conditions, while the triazolylidene complex 2-Mo is unaffected by the presence of pinacol. This supports the observed catalytic potential of 3-Mo, and decreasing TOF values, which already indicated decomposition of the imidazolylidene complex during catalysis (*vide supra*). We propose that the red product present at elevated temperatures in the reaction between 2-Mo and pinacol is a molybdenum oxo pinacolate complex 5-Mo forming under the concomitant release of water (1 equiv.). The released water facilitates hydrolysis back to 2-Mo, which likely explains why complex 5-Mo is not readily isolable after heating. To verify this, the experiment was repeated in the presence of a water scavenger, *i.e.* molecular sieves, giving access to 5-Mo (Scheme 3). The ^1^H-NMR spectrum of 5-Mo shows a slight redistribution of the ligand resonances along with two singlets in the aliphatic region integrating to 12 protons, which are attributed to the pinacolate-CH_3_ groups (Fig. S21†). These can also be found in the ^13^C NMR spectrum of 5-Mo, which shows two distinct resonances at 25.6 and 25.8 ppm (Fig. S22†). Additionally, a quaternary carbon resonance at *δ*_C_ = 97.0 ppm in 5-Mo is observed, corresponding to the carbon atom adjacent to the pinacolate oxygen atoms. Full proof of the molecular structure was obtained *via* X-ray diffraction analysis (Fig. 5). Suitable single crystals were grown from a concentrated diethyl ether solution. Complex 5-Mo crystallises in the *orthorhombic* space group *P*2_1_2_1_2_1_ in a distorted octahedral geometry. The attachment of the pinacolate leads to a slight stretching of the ligand-metal bonds with respect to the parent dioxo complex 2-Mo. Bond lengths for the phenolates (Mo1–O1 and Mo1–O2) are 2.013(9) Å and 2.044(8) Å, respectively. The carbene bond is stretched to 2.187(12) Å and the terminal oxide bond length is found at 1.699(8) Å, comparable to the dioxo complex (see Tables S1 and S2† for more information). Focussing on tungsten, no reaction between 2-W/3-W and pinacol was observed, neither at room temperature nor at 130 °C in C_6_D_6_. This suggests that the oxo groups in the tungsten framework are less basic compared to those in the molybdenum complexes. This could also be one reason why the tungsten complexes are completely inactive in the catalytic deoxygenation of nitroarenes (Table 1, entries 7 and 8), as the initial reaction step (deprotonation of pinacol) appears to be thermodynamically hindered.

To further demonstrate the participation of pinacolate complex 5-Mo in the deoxygenation reaction, stoichiometric reactions starting from *o*-nitrosotoluene or *o*-nitrotoluene were conducted. For each oxygen atom to be removed one equivalent of pinacolate complex was added (overall stoichiometry 2 : 1 for 5-Mo:nitrotoluene and 1 : 1 for 5-Mo:nitrosotoluene) and the reaction progress was monitored by ^1^H-NMR spectrometry (Fig. S67 and S68†). As expected, no reaction occurred at room temperature. But when heated to 130 °C, the blood red mixtures turned dark brown within one hour and light orange within four hours. After 12 hours, the ^1^H-NMR spectrum showed complete conversion of 5-Mo to 2-Mo.

Although in many cases a Mo^IV^ species is proposed as the catalytically active species, it is also known that such Mo^IV^ complexes immediately comproportionate with remaining Mo^VI^ to form μ-oxo bridged dimeric Mo^V^ compounds.^29,34,39^ Thus, the thermolysis of 5-Mo was conducted in the presence of one equivalent of 2-Mo (Scheme 3).^70^ The mixture turned dark brown within 2 hours at 130 °C and the corresponding NMR spectrum suggests the presence of a paramagnetic compound 7-Mo (Scheme 3 and Fig. S26, S27†).^70^

To verify the assumption that this new compound arises from reduction and is indeed a μ-oxo bridged dimeric complex, 2-Mo was also reduced with tri-*n*-butylphosphine (P(^*n*^Bu)_3_) at 130 °C (Scheme 3). After the work-up, a dark grey powder was obtained, and the NMR signature is in-line with the results from thermolysis of 5-Mo, indicating the formation of 7-Mo (Fig. S26†). An effective magnetic moment of 1.94 *μ*_B_ was observed, which is consistent with previously reported μ-oxo dimolybdenum(v) complexes.^70^ To unambiguously determine the molecular structure of this complex, single crystals were grown from a concentrated toluene solution of the complex at −40 °C (Fig. 5). Complex 7-Mo crystallises in the *triclinic* space group *P*1̄ and each of the two molybdenum centres is pentacoordinate in a distorted trigonal pyramidal geometry (*τ*_5_ = 0.38 for Mo1 and 0.13 for Mo1A). The Mo1–O1–Mo1A bridge was found to be slightly deviating from linearity, showing an angle of 162.62(17)° with Mo–O distances of 1.890(3) Å (Mo1–O10) and 1.896(3) Å (Mo1A–O10). These Mo–O distances are substantially longer compared to the terminal Mo=O units at 1.656(3) Å (Mo1–O11) and 1.667(3) Å (Mo1A–O11A). The molybdenum carbene distance was found to be 2.123(4) Å and 2.124(4) Å for Mo1–C1 and Mo1A–C1A respectively, indicating some minor back-bonding effects from the d^1^ Mo centre. Similar observations have already been made in other d^1^ NHC/MIC complexes with vanadium^60,61^ and molybdenum.^70,108^

**Fig. 6:**
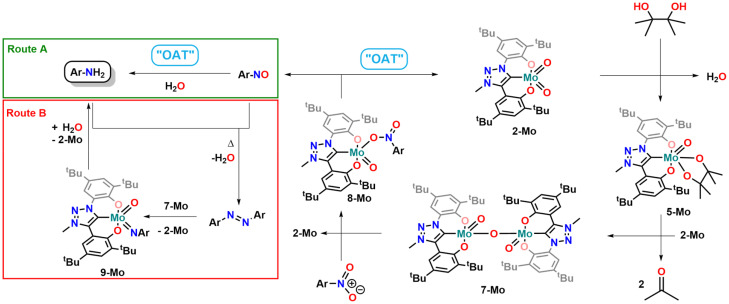
Proposed mechanism for the oxygen-atom-transfer reaction mediated by 2-Mo in the presence of pinacole in catalytic reduction of nitroarenes (right part) and the proposed mechanism of nitrosoarene reduction *via* direct deoxygenation reaction (Route A) or *via* azoarene cleavage (Route B/left part).

To further check whether a Mo^IV^ species could also be isolated we attempted various trapping strategies. However, all attempts to accomplish this, *i.e.* conducting the deoxygenation in the presence of stabilizing ligands (*e.g.* PMe_3_), remained unsuccessful. A different strategy, that led to the successful isolation of Re^V^ di-oxo compounds from Re^VII^ tri-oxo compounds, was the addition of alkynes to generate a corresponding metallacyclopropene complex.^2,109,110^ Similar strategies have also been used to prepare “masked” low-valent M^IV^ halide complexes of molybdenum^111–115^ and tungsten.^111,116–118^ In analogy to this, an NMR sample of complex 5-Mo was mixed with an excess of 3-hexyne and heated to 130 °C. The NMR spectrum however does not show a diamagnetic metallacyclopropene complex but the dimeric μ-oxo bridged complex 7-Mo was found instead (Fig. S71†). These findings suggest that no Mo^IV^ complex is formed during the catalytic process (Fig. 6, right part) and that the catalytic active complex is indeed the μ-oxo bridged dimeric complex 7-Mo. Further proof for this assumption was brought by stoichiometric reactions of the Mo^V^ compound 7-Mo and *o*-nitrotoluene or *o*-nitrosotoluene (Fig. S69 and S70†). Both reactions were conducted at room temperature and monitored *via* NMR spectroscopy. The reaction starting from nitrosotoluene is complete within a few minutes and complete deoxygenation of the nitro compound is achieved within 12 hours. In contrast, azobenzene does not react with 5-Mo and 7-Mo at 130 °C within 24 h, which is in accordance with previous experiments using 1-Mo as a catalyst.^70^ This further indicates, that despite changing the NHC donor, the general mechanism of nitroarene deoxygenation remains the same and follows a direct deoxygenation pathway (Route A, Fig. 6) deoxygenating nitroarenes *via* nitrosoarenes to the corresponding anilines, with the “azobenzene pathway” (Route B, Fig. 6) being unlikely.^11,119^ This has also been confirmed computationally in a recent publication by Nieto-Faza and Sanz elucidating the mechanism of molybdenum/pinacol-mediated nitroarene reduction.^120^ Finally, the mechanistic similarity between our previous investigations using 1-Mo as a catalysts,^70^ in combination with the enhanced catalytic potential of 2-Mo in the deoxygenation of nitroarenes, unambiguously confirms that the carbene has a major influence on the catalytic potential of early transition metal complexes.

## Conclusions

We have synthesized and isolated a series of OCO-chelated NHC/MIC complexes of Mo^VI^ and W^VI^. We found that the complexation reaction requires electron-rich carbenes and allows only distinctive combinations of NHC/MIC and MO_2_Cl_2_L precursors (L = DME or none, M = Mo, W). In catalytic deoxygenations, such as the reduction of nitroarenes to anilines, we found that the tungsten complexes are inactive, but that mesoionic carbene based complex 2-Mo (with a triazolylidene donor) exhibits unprecedented catalytic activity, reaching a TOF of 270 h^−1^ at catalyst loadings of 0.05 mol%. Compared to previous systems,^54^ this states an increase of the catalytic activity of over one order of magnitude (30-fold increase). We were further able to show, that the NHC donor has a drastic influence on the catalytic activity, but also stability of the complexes. While *n*NHC based complexes tend to decompose under the conditions chosen (pinacol, 130 °C) the MIC metal carbon was found to be highly stable, and storage of stock solutions and synthesis of this unique triazolylidene complex can be even performed under air and moisture. Mechanistic experiments suggest that the reaction occurs *via* the stepwise formation of a molybdenum(VI) pinacolate complex, that is thermally converted to a μ-oxo dimolybdenum(V) complex, which is capable of directly deoxygenating nitroarenes, *via* nitrosoarenes, to the corresponding anilines, with no azobenzene playing a role in the reduction process.^70^

This is the first report showcasing the drastic influence of NHC and MIC donors on the potential and stability of early transition metal catalysts, highlighting the importance and future of this research field and the necessity to further study the chemistry and potential of MIC ligands in early transition metal chemistry.

## Author contributions

The project was designed by FRN and SH. Complex synthesis was carried out by FRN, FH and MB. Catalytic experiments were carried out by FRN, FH and PB. Mechanistic investigations were performed by FRN. FRN recorded all experimental data/spectra except for X-ray diffraction analysis, which was performed by SH, MS and JB. The manuscript was written by FRN and SH and final versions were proof read and acknowledged by all authors.

## Data availability

NMRs, IR and cyclic voltammograms have been included (plotted) into the ESI.† Raw data is stored on the university servers and can be accessed *via* us if necessary.

Crystallographic data (CIF-files) have been uploaded to the CCDC and can be obtained *via* the CCDC homepage using the CCDC numbers assigned in the ESI.† Raw data and frames are stored on the university servers and can be accessed *via* us if necessary.

## Conflicts of interest

There are no conflicts to declare.

## Supplementary Material

QI-012-D4QI02392G-s001

QI-012-D4QI02392G-s002
