# Supplementary material for: Probing the influence of imidazolylidene- and triazolylidene-based carbenes on the catalytic potential of dioxomolybdenum and dioxotungsten complexes in deoxygenation catalysis
Source: Inorg Chem Front. 2024 Dec 24;12(6):2224–35. doi: 10.1039/d4qi02392g (PMC11771132; doi:10.1039/d4qi02392g)
Supplement: QI-012-D4QI02392G-s001 [file QI-012-D4QI02392G-s001.pdf]

Supplementary Information (SI) for Inorganic Chemistry Frontiers.
This journal is © the Partner Organisations 2025

Electronic Supporting Information (ESI) for

Probing The Influence of Imidazolylidene and Triazolylidene Donors on the Catalytic Potential of Dioxomolybdenum and Dioxotungsten Deoxygenation Catalysts

Florian R. Neururer,[a] Florian Heim [a] Marc Baltrun[a], Philipp Boos[b], Julia Beerhues[c], Michael Seidl[a] and Stephan Hohloch*[a]

1. General considerations 2
2. Synthetic procedures 2
 [$Mo^{VI}L^2O_2$] (2-Mo) 2
 [$W^{VI}L^2O_2$] (2-W) 3
 [$Mo^{VI}L^3O_2$] (3-Mo) 3
 [$W^{VI}L^3O_2$] (3-W) 4
 [$Mo^{VI}L^2O$(pin)] (5-Mo) 4
 [($Mo^{V}OL^2)_2(\mu$-O)] (7-Mo) 5
3. Catalytic transformations 6
 General remarks 6
 Catalyst screening 6
 Time dependent catalysis 6
 Substrate screening 6
4. Mechanistic investigations 9
 Stoichiometric deoxygenation reactions mediated by 5-Mo 9
 Stoichiometric deoxygenation reactions mediated by 7-Mo 9
5. NMR spectra 10
6. IR spectroscopy 50
7. UV-Vis-NIR spectra 52
8. Crystallographic details 55
9. Literature 59

1. General considerations

Unless stated otherwise, all transformations were conducted under an inert atmosphere using standard Schlenk techniques or an argon filled glove box. High temperature reactions were carried out in preheated oil baths or aluminium blocks. Low temperature reactions were performed using a precooled aluminium block or dewar vessels filled with cooling mixtures consisting of ethanol/liquid nitrogen. Solvents for synthetic purposes were purified using an *MBraun* SPS system and stored over activated molecular sieves. Triethylamine was dried by refluxing over calcium hydride, subsequent distillation, and storage over activated molecular sieves. Solvents used for aqueous workup or chromatography (technical grade) were used as received by a commercial supplier. Chemicals and reagents were purchased from *Sigma Aldrich*, *BLD* or *Alfa Aesar* and used as received. Deuterated solvents (benzene-d_6 and dichloromethane-d_2) were stored over activated molecular sieves. Chloroform-*d* and dimethylsulfoxide-d_6 were used as received. Ligand precursors (H_3L^1Cl[1,2], H_3L^2Cl[3], H_3L^3Cl[1]) and complex **1-Mo**[4] were prepared following literature known procedures. NMR spectra were recorded on a *Bruker Ascend 400* spectrometer. ^{1}H and ^{13}C{^{1}H} chemical shifts are reported in ppm and calibrated using residual solvent resonances. Elemental analyses (C, H, N) were performed on a *Vario Microtube* instrument. GC-MS experiments were conducted using a *Shimadzu Nexis GC 2030* instrument equipped with a flame ionization detector and an autosampler. ATR-Infrared spectroscopy was conducted on a *Bruker Alpha* IR spectrometer. X-ray diffraction crystallography was performed at the University of Innsbruck. All crystals were kept at 120(2) K or 153(2) K throughout data collection. Data collection was performed using the ApexIV software package on a Bruker D8 Quest instrument. Data refinement and reduction was performed using the Bruker ApexIV suite 2021. All structures were solved with SHELXT[5] and refined using the OLEX 2 software package.[6] Strongly disordered solvent molecules have been removed using the SQUEEZE[7] operation. All nonhydrogen atoms were refined anisotropically, and hydrogen atoms were included at the geometrically calculated positions and refined using a riding model.

2. Synthetic procedures

[$Mo^{VI}L^2O_2$] (2-Mo)

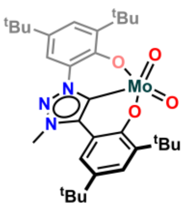

To a 100 mL Schlenk flask containing a solution of the ligand precursor H_3L^2Cl (700 mg, 1.0 equiv., 1.33 mmol) in THF (10 mL), triethylamine (0.65 mL, 3.5 equiv., 4.66 mmol) was added *via* syringe at ambient temperature. The resulting bright orange suspension was kept at room temperature for approx. 60 minutes, before it was added dropwise to a 100 mL J. Young Schlenk flask containing a solution of [MoO_2Cl_2(dme)] (384 mg, 1 equiv., 1.33 mmol) in THF (10 mL). The suspension gradually turned bright yellow. After 15 to 20 hours, triethylammonium chloride was removed *via* cannula filtration and the residue was washed with THF (3 x 5 mL). The filtrate was concentrated until some precipitation (approx. 5 mL) was observed. This was completed by slow addition of *n*-hexane (50 mL) and vigorous stirring at ambient temperature for approx. 30 minutes. The pale-yellow precipitate was collected *via* filtration through a medium porosity Schlenk-frit, washed with *n*-hexane (3 x 10 mL) and dried *in vacuo* to obtain a yellow powder. Yield: 91% (752 mg, 1.22 mmol).

Alternative work-up: The bright yellow suspension was transferred into a 100 mL round bottom flask and the THF was removed on a rotary evaporator. The residue was dissolved in dichloromethane (25 mL), washed with water (3 x 10 mL), brine (2 x 10 mL), dried over anhydrous sodium sulphate and evaporated to dryness. The residue was suspended in hexanes and filtered.

^{1}H NMR (400 MHz, Benzene-d_6) δ 8.08 (d, *J* = 2.4 Hz, 1H), 7.65 (d, *J* = 2.3 Hz, 1H), 7.64 (d, *J* = 2.4 Hz, 1H), 7.09 (d, *J* = 2.3 Hz, 1H), 3.24 (s, 3H), 1.66 (s, 18H), 1.37 (s, 9H), 1.33 (s, 9H).

$^{13}C\{^{1}H\}$ NMR (101 MHz, Benzene-d_6) δ 159.2, 157.7, 151.0, 143.7, 143.2, 142.3, 141.8, 141.2, 126.8, 126.0, 125.0, 118.6, 113.6, 113.4, 39.8, 36.3, 36.2, 34.8, 34.7, 31.8, 31.7, 30.4, 30.3.

IR (cm^{-1}): 2957, 1525, 1486, 1362, 1301, 1258, 1237, 1153, 1129, 1029, 933, 904, 874, 861, 800, 772, 759, 712, 694, 639, 559, 469, 451.

UV-Vis-NIR: λ_{max} = 326 nm (ε= 29770 L mol^{-1} cm^{-1}), 360 nm (ε = 32440 L mol^{-1} cm^{-1}).

Elemental analysis (%) *calc'd* for $C_{31}H_{43}MoN_3O_4$: C, 60.28; H, 7.02; N, 6.80; found C, 60.19; H, 7.21; N, 6.46.

$[W^{VI}L^{2}O_2]$ (2-W)

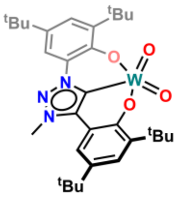

Following the same procedure as described for complex **2-Mo**, starting from H_3L^2Cl (700 mg, 1.0 equiv., 1.33 mmol), triethylamine (0.65 mL, 3.5 equiv., 4.66 mmol) and $[W^{VI}O_2Cl_2]$ (381 mg, 1.0 equiv., 1.33 mmol). Off-white solid. Yield: 70% (655 mg, 0.245 mmol).

^{1}H NMR (400 MHz, Benzene-d_6) δ 8.06 (d, *J* = 2.4 Hz, 1H), 7.66 (d, *J* = 2.3 Hz, 1H), 7.65 (d, *J* = 2.4 Hz, 1H), 7.12 (d, *J* = 2.4 Hz, 1H), 3.31 (s, 3H), 1.69 (d, *J* = 3.3 Hz, 18H), 1.35 (s, 9H), 1.32 (s, 9H).

^{1}H NMR (400 MHz, Dichloromethane-d_2) δ 8.13 (d, *J* = 2.4 Hz, 1H), 7.80 (d, *J* = 2.3 Hz, 1H), 7.65 (d, *J* = 2.3 Hz, 1H), 7.57 (d, *J* = 2.5 Hz, 1H), 4.71 (s, 3H), 1.48 (s, 9H), 1.48 (s, 9H), 1.41 (s, 9H), 1.40 (s, 9H).

$^{13}C\{^{1}H\}$ NMR (101 MHz, Dichloromethane-d_2) δ 168.1, 156.5, 149.5, 145.1, 144.1, 143.4, 142.8, 141.9, 128.1, 126.8, 125.5, 119.0, 114.0, 41.9, 36.3, 36.2, 35.3, 35.2, 31.8, 31.7, 30.3, 30.2.

IR (cm^{-1}): 2961, 2871, 1521, 1486, 1460, 1423, 1394, 1362, 1292, 1256, 1245, 1202, 1158, 1129, 1076, 1055, 949, 923, 904, 878, 861, 798, 770, 759, 712, 694, 643, 557, 467, 416.

UV-Vis-NIR: λ_{max} = 352 nm (ε = 71240 L mol^{-1} cm^{-1}).

Elemental analysis (%) *calc'd* for $C_{31}H_{43}N_3O_4W$: C, 52.77; H, 6.14; N, 5.96; found C, 52.09; H, 6.32; N, 5.96.

$[Mo^{VI}L^{3}O_2]$ (3-Mo)

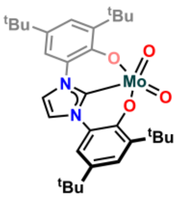

In an argon filled glovebox, a 20 mL scintillation vial was charged with H_3L^3Cl (128 mg, 1.0 equiv., 0.250 mmol) and THF (1.5 mL). A solution of triethylamine (89 mg, 3.5 equiv., 0.875 mmol) in THF (1 mL) was added at room temperature. After 30 minutes at room temperature, the suspension was added into a 20 mL scintillation vial containing a solution of $[MoO_2Cl_2(dme)]$ (72 mg, 1.0 equiv., 0.250 mmol) in THF (1 mL), resulting in a brown suspension. After 15 hours at room temperature, the mixture was filtered through a pipette equipped with a glass fibre filter. The filtrate was evaporated to dryness and the residue was triturated with *n*-hexane to afford a brown powder. The crude product was redissolved in THF (2 mL), concentrated, layered with *n*-hexane and cooled to –40 °C. Pale yellow crystals were obtained within 2 days, separated from the solution, and dried *in vacuo*. Yield: 66% (99 mg, 0.16 mmol).

^{1}H NMR (400 MHz, Benzene-d_6) δ 7.54 (d, *J* = 2.2 Hz, 2H), 7.03 (d, *J* = 2.2 Hz, 2H), 6.94 (s, 2H), 1.65 (s, 18H), 1.34 (s, 18H).

$^{13}C\{^{1}H\}$ NMR (101 MHz, Benzene-d_6) δ 171.9, 149.6, 142.4, 141.5, 125.6, 123.6, 118.4, 112.7, 36.2, 34.7, 31.7, 30.3.

IR (cm^{-1}): 2955, 2869, 1478, 1448, 1394, 1360, 1327, 1268, 1245, 1217, 1149, 1102, 933, 908, 855, 843, 774, 755, 727, 678, 667, 641, 559, 518, 451, 437.

UV-Vis-NIR: λ_{max} = 326 nm (ε = 34480 L mol^{-1} cm^{-1}).

Elemental analysis (%) *calc'd* for $C_{31}H_{42}MoN_2O_4$ C, 61.79; H, 7.03; N, 4.65; found C, 61.49; H, 7.39; 4.29.

[W^{VI}L^{3}O$_2$] (3-W)

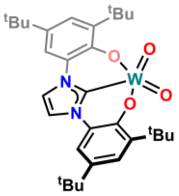

Following the same procedure as described for complex **3-Mo**, starting from H_3L^3Cl (75 mg, 1.0 equiv., 0.146 mmol mmol), triethylamine (59 mg, 4.0 equiv., 0.59 mmol) and [W^{VI}O$_2$Cl$_2$] (42 mg, 1.0 equiv., 0.146 mmol). Off-white solid. Yield: 18% (18 mg, 0.026 mmol).

^{1}H NMR (400 MHz, Benzene-*d*$_6$) δ 7.55 (d, *J* = 2.2 Hz, 2H), 7.04 (d, *J* = 2.0 Hz, 4H), 1.69 (s, 18H), 1.32 (s, 18H).

^{1}H NMR (400 MHz, Dichloromethane-*d*$_2$) δ 8.28 (s, 2H), 7.51 (d, *J* = 2.3 Hz, 2H), 7.50 (d, *J* = 2.2 Hz, 2H), 1.49 (s, 18H), 1.40 (s, 18H).

^{13}C{^{1}H} NMR (101 MHz, Dichloromethane-*d*$_2$) δ 181.1, 148.0, 144.2, 142.1, 126.0, 124.3, 120.0, 113.2, 36.1, 35.1, 31.7, 30.2.

IR (cm^{-1}): 2957, 1480, 1448, 1394, 1362, 1323, 1266, 1100, 955, 917, 845, 774, 757, 719, 674, 557, 486, 431.

UV-Vis-NIR: λ_{max} = 327 nm (ε = 30970L mol^{-1} cm^{-1}).

Elemental analysis (%) *calc'd* for $C_{31}H_{42}WN_2O_4$ C, 53.92; H, 6.13; N, 4.06; found C, 54.22; H, 6.46; 4.04.

[Mo^{VI}L^{2}O(pin)] (5-Mo)

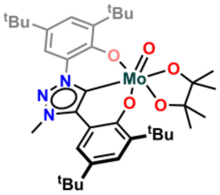

In an argon filled glovebox, a 100 mL J. Young Schlenk flask was charged with complex **2-Mo** (99 mg, 1.0 equiv., 0.160 mmol), pinacol (19 mg, 1.0 equiv., 0.160 mmol), 3 Å molecular sieves and toluene (10 mL). The flask was closed, brought outside of the glovebox, and heated to 90 °C. The bright yellow mixture turned blood red within 30 minutes. After 15 hours, the solvent was removed *in vacuo*. The residue was extracted with pentane (3 x 5 mL), concentrated, and crystallized at –40 °C. Crystalline material was obtained within a few days. This procedure was repeated twice to remove residual pinacol. Single crystals suitable for x-ray analysis were obtained by slow evaporation of diethyl ether at room temperature. Blood red crystals. Yield: 45 % (52 mg, 0.072 mmol).

^{1}H NMR (400 MHz, Benzene-*d*$_6$) δ 8.13 (d, *J* = 2.4 Hz, 1H), 7.80 (d, *J* = 2.4 Hz, 1H), 7.78 (d, *J* = 2.4 Hz, 1H), 7.17 (d, *J* = 2.4 Hz, 1H), 3.07 (s, 3H), 1.96 (s, 18H), 1.33 (s, 9H), 1.28 (s, 9H), 1.01 (s, 6H), 0.93 (s, 6H).

^{13}C{^{1}H} NMR (101 MHz, Benzene-*d*$_6$) δ 180.5, 160.6, 154.5, 145.1, 143.7, 143.0, 142.0, 141.9, 126.0, 125.5, 118.5, 116.8, 113.5, 97.1, 38.0, 36.7, 36.5, 34.8, 34.7, 31.7, 31.6, 30.9, 30.9, 25.8, 25.6.

IR (cm^{-1}): 2957, 2867, 1531, 1480, 1427, 1413, 1392, 1360, 1294, 1256, 1198, 1145, 972, 908, 898, 874, 841, 776, 751, 710, 694, 678, 623, 537, 492, 445.

UV-Vis-NIR: λ_{max} = 326 nm (ε = 43520 L mol^{-1} cm^{-1}), 351 nm (ε = 43250 L mol^{-1} cm^{-1}), 358 nm (ε = 43540 L mol^{-1} cm^{-1}), 466 nm (ε = 54720 L mol^{-1} cm^{-1}), 486 nm (ε = 63300 L mol^{-1} cm^{-1}).

Elemental analysis (%) *calc'd* for $C_{37}H_{55}MoN_3O_5$: C, 61.91; H, 7.72; N, 5.85; found C, 61.90; H, 7.81; N, 5.51.

[(Mo^{V}OL^{2})$_2$(μ-O)] (7-Mo)

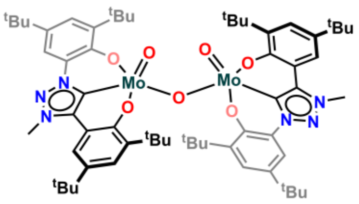

Route A: In an argon filled glovebox, a 100 mL J. Young Schlenk flask was charged with **2-Mo** (125 mg, 1.0 equiv., 0.200 mmol), tri-*n*-butyl phosphine (430 mg, 10.6 equiv., 2.13 mmol) and toluene (20 mL, 0.1 m), giving a bright yellow solution. The flask was capped, brought outside the glovebox, and heated to 130 °C. After 12 hours, volatile materials were removed *in vacuo*. Under vigorous stirring, the residue was suspended in *n*-hexane (10 mL). After 10 minutes, the precipitate was allowed to settle, and the supernatant was removed *via* cannula filtration. The dark grey residue was washed with further portions of *n*-hexane (2 x 5 mL, until the washings came colourless) and dried *in vacuo*. Dark grey, air-sensitive powder. Yield: 60% (73 mg, 0.060 mmol).

Route B: In an argon filled glove box, a J. Young NMR tube was charged with complex **2-Mo** (10 mg, 1.0 equiv., 0.016 mmol), pinacolate complex **5-Mo** (12 mg, 1.0 equiv., 0.016 mmol) and benzene-d_6 (0.4 mL), yielding a blood red solution. A control NMR was recorded. The tube was heated to 130 °C, the solution gradually turned light brown. When all starting materials were consumed (monitored by ^{1}H-NMR spectroscopy), the tube was brought back into the glove box. Solvents were removed *in vacuo* and the residue was extracted with toluene, filtered, and concentrated. Crystalline material was obtained within 2 days at –40 °C.

IR (cm^{-1}): 2955, 2906, 2869, 1607, 1525, 1480, 1445, 1417, 1394, 1362, 1296, 1245, 1200, 1153, 1129, 1072, 1053, 957, 917, 874, 841, 790, 757, 733, 712, 696, 678, 643, 553, 527, 490, 453.

UV-Vis-NIR: λ_{max} = 326 nm (ε = 53730 L mol^{-1} cm^{-1}), 368 nm (ε = 58640 L mol^{-1} cm^{-1}), 388 nm (ε = 55520 L mol^{-1} cm^{-1}).

Elemental analysis (%) *calc'd* for $C_{37}H_{55}MoN_3O_5$: C, 61.07; H, 7.11; N, 6.89; found C, 61.35; H, 7.46; N, 6.15.

3. Catalytic transformations

General remarks

Initially, the FID response factors for *o*-nitrotoluene and *o*-toluidine were determined. This was conducted using a dilution series (c = 1.00, 2.00, 4.00 and 8.28 mmol L^{-1}) of the analyte and mesitylene as an internal standard (c = 4.00 mmol L^{-1}), the ratio of peak areas was plotted against the ratio of concentrations and the slope of the linear function determined *via* linear regression corresponds to the response factor.

GC-MS measurements were conducted using the following the method:

- Injection method/temperature: split/130 °C
- start temperature: 60 °C (hold for 5 min)
- heating rate: 15 °C/min
- end temperature: 300 °C (hold for 5 min)

Catalyst screening

A 10 mL J. Young Schlenk tube was charged with pinacol (4.00 mmol), dry toluene (3 – *x* mL), *o*-nitrotoluene (118 µL, 1.0 equiv., 1.00 mmol) and mesitylene (65.9 µL, 0.5 equiv., 0.500 mmol) in the given order. The mixture was heated to 130 °C in a preheated aluminium block and a given volume *x* mL (*x* = 0.10, 0.25, 1.00 mL) of a previously prepared stock solution (c = 0.01 M) of (pre)catalyst was introduced *via* syringe and the vessel was closed. Within 30 minutes the mixture turned blood red, then dark brown and yellow after 2 - 4 hours. After 6 hours, an aliquot (approx. 25 µL) was removed, filtered through a pipette charged with a short pad of silica (5 mm) and eluted with ethyl acetate (1.5 mL). The composition of this solution was analysed *via* gas chromatography.

Time dependent catalysis

A 10 mL J. Young Schlenk flask was charged with pinacol (473 mg, 4.0 equiv., 4.00 mmol), dry toluene (2.8 mL), *o*-nitrotoluene (118 µL, 1.0 equiv., 1.00 mmol) and mesitylene (65.9 µL, 0.5 equiv., 0.500 mmol). The mixture was stirred vigorously to dissolve all reagents. A 5.0 mM stock solution of the corresponding catalyst **1-Mo**, **2-Mo** or **3-Mo** (0.5 mL, 0.0025 equiv., 0.0025 mmol) was added *via* syringe, the vessel was closed and heated to 130 °C in a preheated aluminium mantle. Aliquots of approx. 25 µL were removed at given times in a stream of argon, filtered through a pipette charged with short pad of silica (approx. 5 mm) and eluted with ethyl acetate (1.5 mL) and analysed using gas chromatography.

Substrate screening

In a 10 mL J. Young Schlenk flask, pinacol (473 mg, 4 equiv., 4.00 mmol) was dissolved in dry toluene (3.5 mL). The corresponding nitro compound (1 mmol) and a 0.01 M solution of the catalyst **2-Mo** in toluene (0.5 mL, 0.5 mol-%) were added. The flask was sealed and heated to 130 °C in a preheated aluminium block. After 15 hours, the mixture was cooled to room temperature and diluted with diethyl ether (10 mL). This mixture was washed with 0.5 M aqueous sodium hydroxide solution (3 x 10 mL). The ether phase was extracted with 1.0 M hydrochloric acid (3-4 x 10 mL). The collected acidic phases were neutralized with 15-20 mL of 2.0 M aqueous NaOH and extracted with dichloromethane (3 x 10 mL). The collected organic phases were dried over anhydrous sodium sulphate and concentrated on a rotary evaporator, providing the corresponding aniline.

Aniline

Light brown oil. Yield: 94%. ^{1}H NMR (400 MHz, Chloroform-*d*) δ 7.17 (m, 2H), 6.78 (tt, *J* = 7.4, 1.1 Hz, 1H), 6.71 (m, 2H), 3.58 (s, 2H). ^{13}C{^{1}H} NMR (101 MHz, Chloroform-*d*) δ 146.3, 129.4, 118.8, 115.3.

p-toluidine

Light brown solid. Yield: 98%. ^{1}H NMR (400 MHz, Chloroform-*d*) δ 6.99 (m, 2H), 6.63 (m, 2H), 3.43 (s, 2H), 2.26 (s, 3H). ^{13}C{^{1}H} NMR (101 MHz, Chloroform-*d*) δ 143.8, 129.9, 128.0, 115.5, 20.6.

4-fluoroaniline

Following the general work-up, this compound still contained approx. 7% of pinacol. (Yield: 103 mg, 99%) Purification was achieved *via* column chromatography (5% to 20% ethyl acetate in petroleum ether). Rf = 0.23 (10% EtOAc in petroleum ether). Yellowish oil. Yield: 68%. ^{1}H NMR (400 MHz, Benzene-d_6) δ 6.69 (m, 2H), 6.03 (m, 2H), 2.54 (s, 2H). ^{13}C{^{1}H} NMR (101 MHz, Benzene-d_6) δ 157.8, 155.5, 143.3, 115.9, 115.8, 115.7. ^{19}F NMR (376 MHz, Benzene-d_6) δ -127.46 (tt, *J* = 9.1, 4.6 Hz).

2-aminophenol

Instead of following the general work-up procedure, the solvent was removed *in vacuo* and the residue was washed with *n*-hexane. Grey solid. Yield: 78%. ^{1}H NMR (400 MHz, Dimethylsulfoxide-d_6) δ 8.90 (s, 1H), 6.63 (dd, *J* = 7.7, 1.5 Hz, 1H), 6.57 (dd, *J* = 7.7, 1.9 Hz, 1H), 6.53 (td, *J* = 7.4, 1.4 Hz, 1H), 6.38 (td, *J* = 7.4, 1.9 Hz, 1H), 4.45 (s, 2H). ^{13}C{^{1}H} NMR (101 MHz, Dimethylsulfoxide-d_6) δ 144.0, 136.5, 119.5, 116.4, 114.4.

4-amino-2,6-dimethylphenol

Instead of following the general work-up procedure, the solvent was removed *in vacuo* and the residue was washed with *n*-hexane (20 mL). Light brown solid. Yield: 95%. ^{1}H NMR (400 MHz, DMSO-d_6) δ 7.15 (s, 1H), 6.16 (s, 1H), 4.31 (s, 2H), 2.03 (s, 3H). ^{13}C NMR (101 MHz, DMSO-d_6) δ 143.9, 140.8, 125.2, 114.3, 16.8.

2'-aminoacetophenone

Brown oil. Yield: 91%. ^{1}H NMR (400 MHz, Chloroform-*d*) δ 7.72 (dd, *J* = 8.0, 1.5 Hz, 1H), 7.27 (m, 1H), 6.68 (m, 2H), 6.31 (s, 2H), 2.58 (s, 3H). ^{13}C{^{1}H} NMR (101 MHz, Chloroform-*d*) δ 200.9, 149.8, 134.5, 132.1, 118.7, 117.6, 116.3, 28.0.

4'-aminoacetophenone

Yellow solid. Yield: 96%. ^{1}H NMR (400 MHz, Chloroform-*d*) δ 7.80 (m, 2H), 6.64 (m, 2H), 4.14 (s, 2H), 2.50 (s, 3H). ^{13}C{^{1}H} NMR (101 MHz, Chloroform-*d*) δ 196.6, 151.3, 130.9, 128.0, 113.8, 26.2.

4-aminobenzonitrile

Off-white solid. Yield: 96%. ^{1}H NMR (400 MHz, Dimethylsulfoxide-d_6) δ 7.38 (m, 2H), 6.61 (m, 2H), 5.93 (s, 2H). ^{13}C{^{1}H} NMR (101 MHz, Dimethylsulfoxide-d_6) δ 152.9, 133.4, 120.7, 113.5, 95.6.

4-anisidine

Reddish-brown solid. Yield: 77%. ^{1}H NMR (400 MHz, Chloroform-*d*) δ 6.75 (m, 2H), 6.65 (m, 2H), 3.74 (s, 3H), 3.37 (s, 2H). ^{13}C{^{1}H} NMR (101 MHz, Chloroform-*d*) δ 153.0, 140.0, 116.6, 114.9, 55.9.

3-aminobenzamide

Instead of following the general work-up procedure, the reaction mixture was evaporated and washed with hexane and diethyl ether. Off-white powder. Yield: 93%. ^{1}H NMR (400 MHz, Dimethylsulfoxide-*d*$_6$) δ 7.71 (s, 1H), 7.12 (s, 1H), 7.04 (m, 2H), 6.98 (dt, *J* = 7.8, 1.3 Hz, 1H), 6.68 (m, 1H), 5.17 (s, 2H). ^{13}C NMR (101 MHz, Dimethylsulfoxide-*d*$_6$) δ 168.8, 148.6, 135.2, 128.6, 116.5, 114.7, 113.1.

Methyl 4-aminobenzoate

White solid. Yield: 80%. ^{1}H NMR (400 MHz, Chloroform-*d*) δ 7.84 (m, 2H), 6.63 (m, 2H), 4.09 (s, 2H), 3.84 (s, 3H). ^{13}C NMR (101 MHz, Chloroform-*d*) δ 167.3, 151.0, 131.7, 119.7, 113.9, 51.7. ^{13}C NMR (101 MHz, Benzene-*d*$_6$) δ 168.7, 151.2, 134.1, 131.6, 116.7, 116.1, 110.9, 51.0.

Methyl 2-aminobenzoate

Pale yellow oil. Yield: 79%. ^{1}H NMR (400 MHz, Benzene-*d*$_6$) δ 7.97 (dd, *J* = 8.1, 1.6 Hz, 1H), 7.00 (ddd, *J* = 8.5, 7.1, 1.6 Hz, 1H), 6.50 (ddd, *J* = 8.2, 7.1, 1.1 Hz, 1H), 6.17 (dd, *J* = 8.3, 1.1 Hz, 1H), 5.44 (s, 2H), 3.42 (s, 3H).

3-Ethynylaniline

Reddish-brown oil. Yield: 84%. ^{1}H NMR (400 MHz, Chloroform-*d*) δ 7.10 (t, *J* = 7.8 Hz, 1H), 6.91 (dt, *J* = 7.6, 1.2 Hz, 1H), 6.81 (t, *J* = 2.0 Hz, 1H), 6.66 (ddd, *J* = 8.1, 2.4, 1.0 Hz, 1H), 3.64 (s, 2H), 3.03 (s, 1H). ^{13}C NMR (101 MHz, Chloroform-*d*) δ 146.4, 129.4, 122.8, 122.6, 118.4, 115.9, 84.0, 76.6.

2-chloro-p-toluidine

Light brown to colourless oil. Yield: 90%. ^{1}H NMR (400 MHz, Chloroform-*d*) δ 7.08 (d, *J* = 1.0 Hz, 1H), 6.88 (dd, *J* = 8.0, 1.2 Hz, 1H), 6.69 (d, *J* = 8.1 Hz, 1H), 3.94 (s, 2H), 2.23 (s, 3H). ^{13}C NMR (101 MHz, Chloroform-*d*) δ 140.3, 129.8, 129.0, 128.4, 119.4, 116.1, 20.3.

2-aminopyridine

Instead of following the general work-up procedure, the crude mixture was evaporated and subjected to flash column chromatography (0-70% ethyl acetate/hexane). Brownish solid. Yield: 81%. R_f = 0.12 (50% ethyl acetate/hexane) ^{1}H NMR (400 MHz, Chloroform-*d*) δ 8.05 (ddd, *J* = 5.2, 1.9, 0.9 Hz, 1H), 7.43 (ddd, *J* = 8.3, 7.2, 1.9 Hz, 1H), 6.64 (ddd, *J* = 7.2, 5.1, 1.0 Hz, 1H), 6.50 (dt, *J* = 8.3, 1.0 Hz, 1H), 4.40 (s, 2H). ^{13}C NMR (101 MHz, Chloroform-*d*) δ 158.4, 147.9, 138.1, 114.1, 108.9.

6-aminoquinoline

Additional to the general work-up procedure, the residue was washed with hexane (2 x 5 mL) and diethyl ether (2 x 5 mL). Orange-brown powder. Yield: 89%. ^{1}H NMR (400 MHz, Benzene-*d*$_6$) δ 8.66 (dd, *J* = 4.1, 1.7 Hz, 1H), 8.14 (d, *J* = 8.9 Hz, 1H), 7.47 (dt, *J* = 8.3, 1.2 Hz, 1H), 6.82 (dd, *J* = 8.3, 4.1 Hz, 1H), 6.62 (dd, *J* = 8.9, 2.6 Hz, 1H), 6.35 (d, *J* = 2.6 Hz, 1H), 3.08 (s, 2H). ^{13}C NMR (101 MHz, Benzene-*d*$_6$) δ 146.9, 145.2, 144.4, 133.1, 131.3, 130.2, 121.5, 121.5, 107.1.

4. Mechanistic investigations

Stoichiometric deoxygenation reactions mediated by 5-Mo

Inside an argon filled glove box, a J. Young NMR tube was charged with **5-Mo**, *o*-nitrosotoluene or *o*-nitrotoluene and benzene-d_6 (0.4 mL). A ^{1}H-NMR spectrum was recorded, and the tube was heated to 130 °C. The mixture turned light brown within 2 hours. After 12 h at 130 °C, the vessel was cooled to room temperature and another ^{1}H-NMR spectrum was recorded. The tube was brought back inside the glove box and the solvent was evaporated. The crude residue was washed with hexane and dried *in vacuo*, providing a bright yellow powder, which was identified as **2-Mo**.

2-nitrosotoluene

From **5-Mo** (12 mg, 1.0 equiv., 0.017 mmol) and *o*-nitrosotoluene (2.0 mg, 1.0 equiv., 0.17 mmol).

2-nitrotoluene

From **5-Mo** (20 mg, 1.0 equiv., 0.028 mmol) and *o*-nitrotoluene (1.9 mg, 0.5 equiv., 0.014 mmol).

Stoichiometric deoxygenation reactions mediated by 7-Mo

2-nitrosotoluene

Inside an Argon filled glove box, a J. Young NMR tube was charged with **7-Mo** (10 mg, 1 equiv., 0.008 mmol) and a solution of 2-nitrosotoluene (1 mg, 1 equiv., 0.008 mmol) in benzene-d_6 (0.4 mL) was added. The dark brown solution turns bright orange within a few seconds. According to ^{1}H-NMR spectroscopy, complex **2-Mo** is formed quantitatively along with some unknown product resulting from the deoxygenation of the nitroso compound.

2-nitrotoluene

Inside an Argon filled glove box, a J. Young NMR tube was charged with **7-Mo** (18 mg, 1.0 equiv., 0.015 mmol) and a solution of 2-nitrotoluene (1 mg, 0.5 equiv., 0.007 mmol) in benzene-d_6 (0.4 mL) The reaction was monitored *via* ^{1}H-NMR spectroscopy. In contrast to the deoxygenation of 2-nitrosotoluene, this reaction proceeds at a considerably slower rate. Complete conversion is observed within 12 hours at room temperature, forming a red solution.

5. NMR spectra

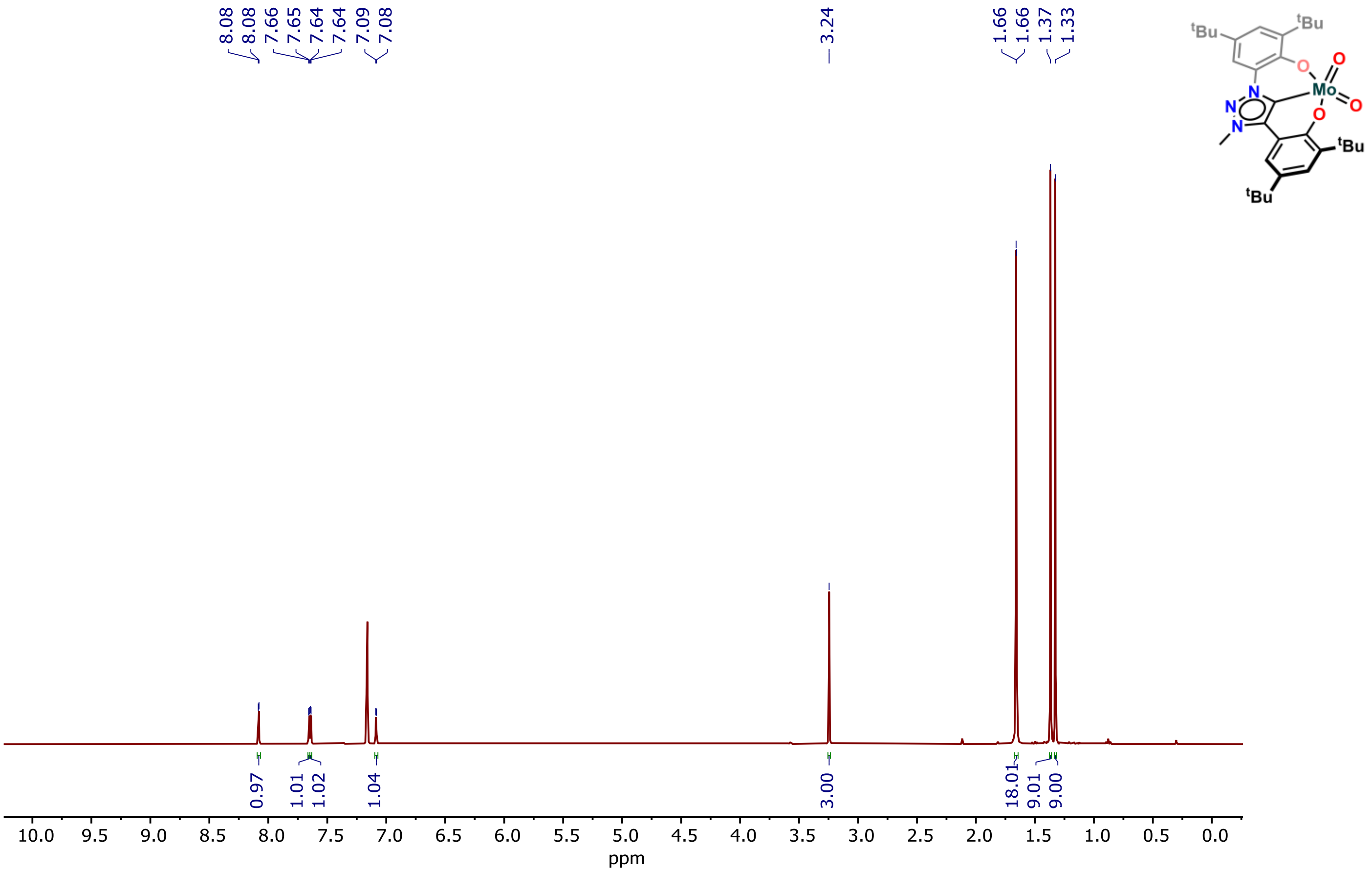

Figure S 1: ^{1}H-NMR spectrum of **2-Mo** in benzene-d_6 at 298 K.

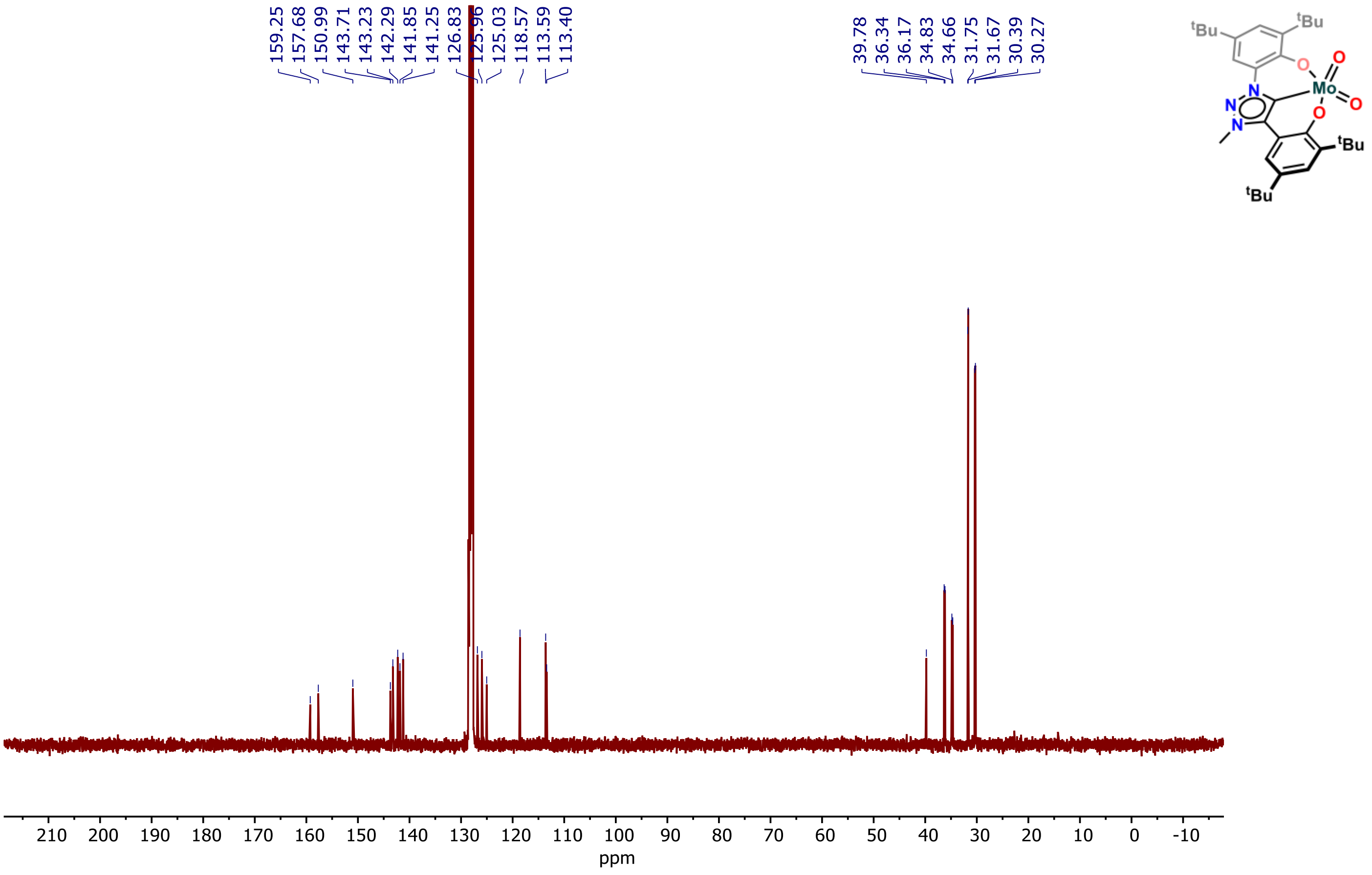

Figure S 2: $^{13}C\{^{1}H\}$-NMR spectrum of **2-Mo** in benzene-d_6 at 298 K.

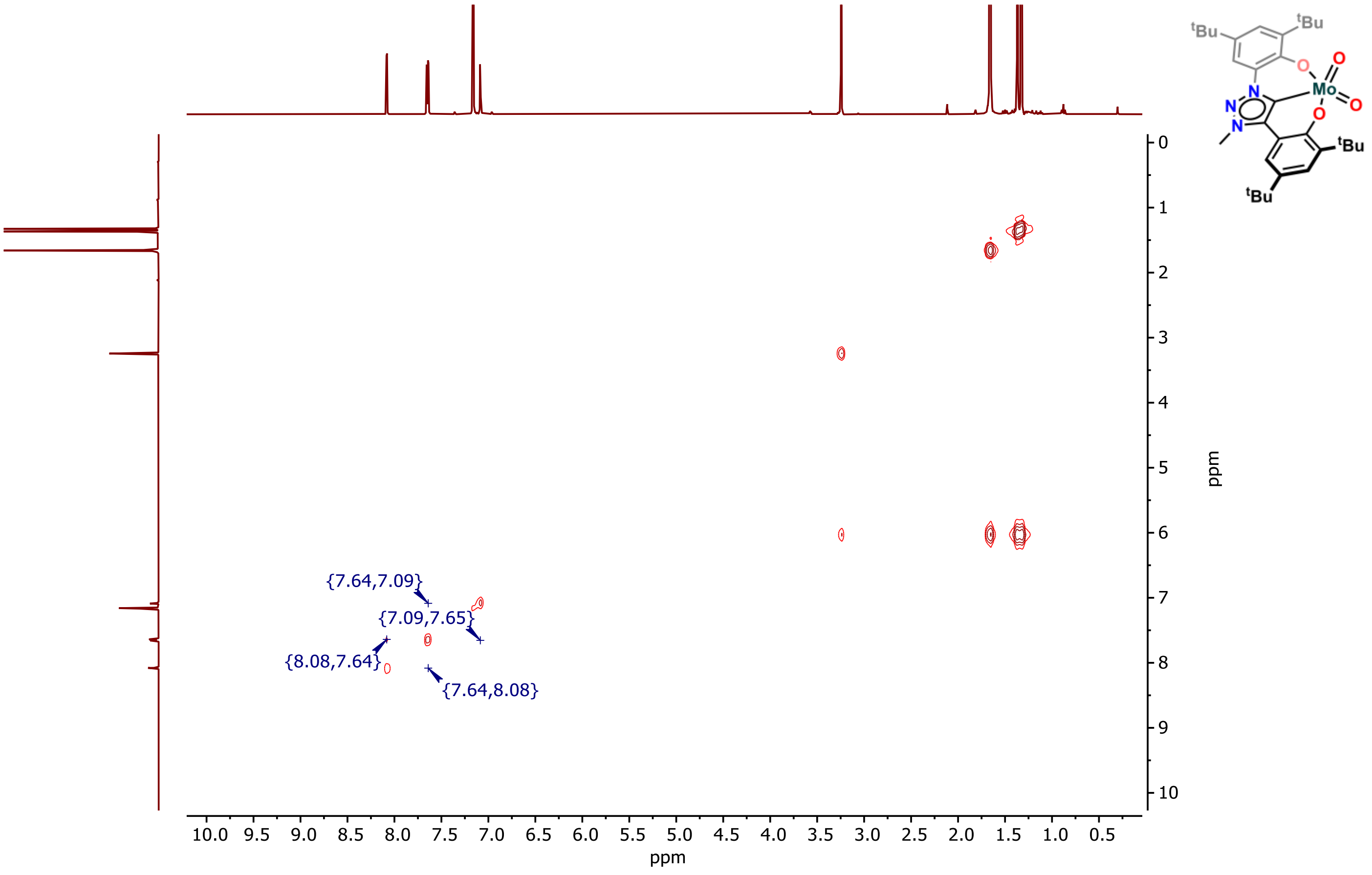

Figure S 3: ^{1}H-^{1}H COSY spectrum of **2-Mo** in benzene-d_6 at 298 K.

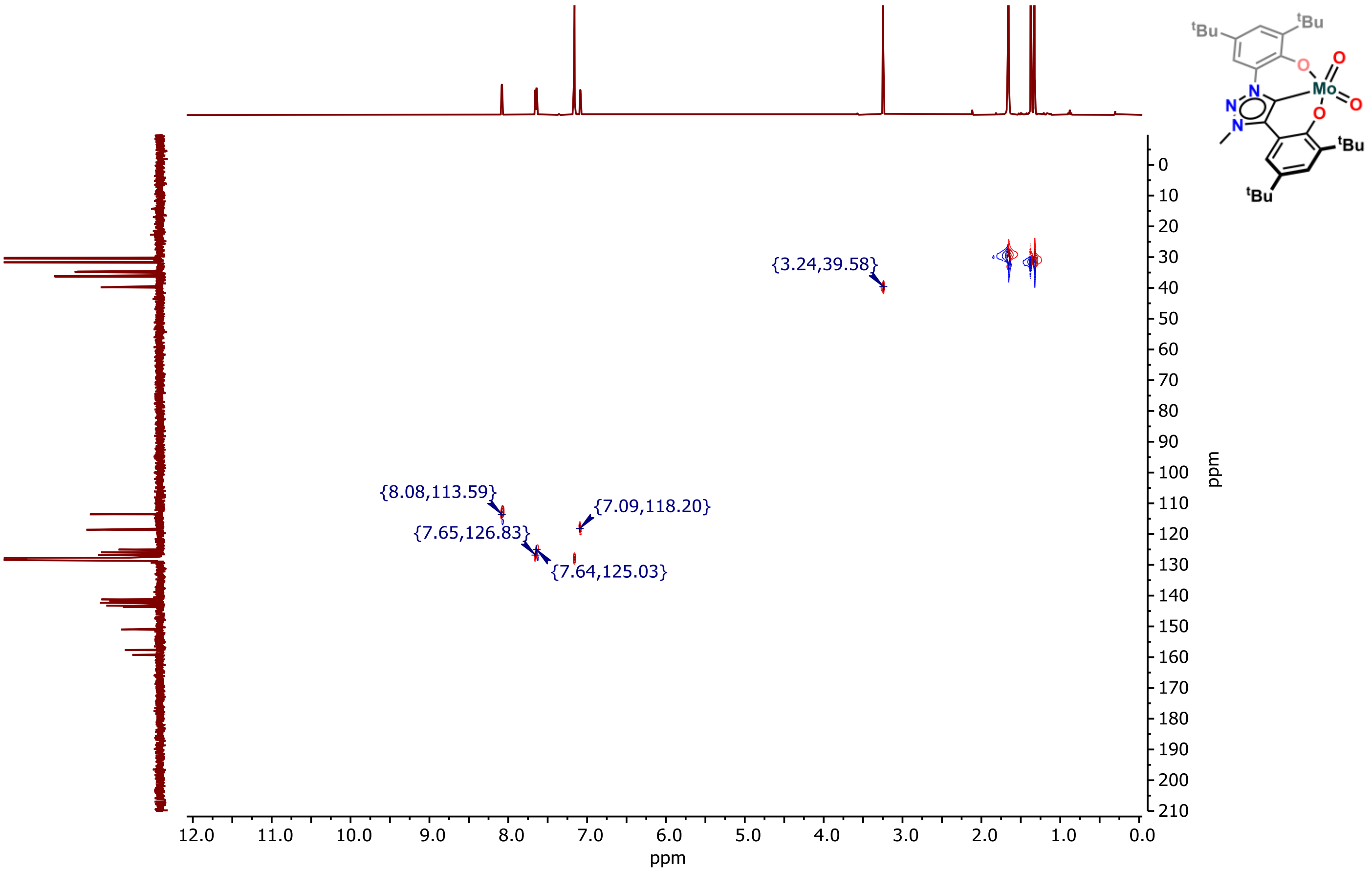

Figure S 4: ^{1}H-^{13}C HSQC spectrum of **2-Mo** in benzene-d_6 at 298 K.

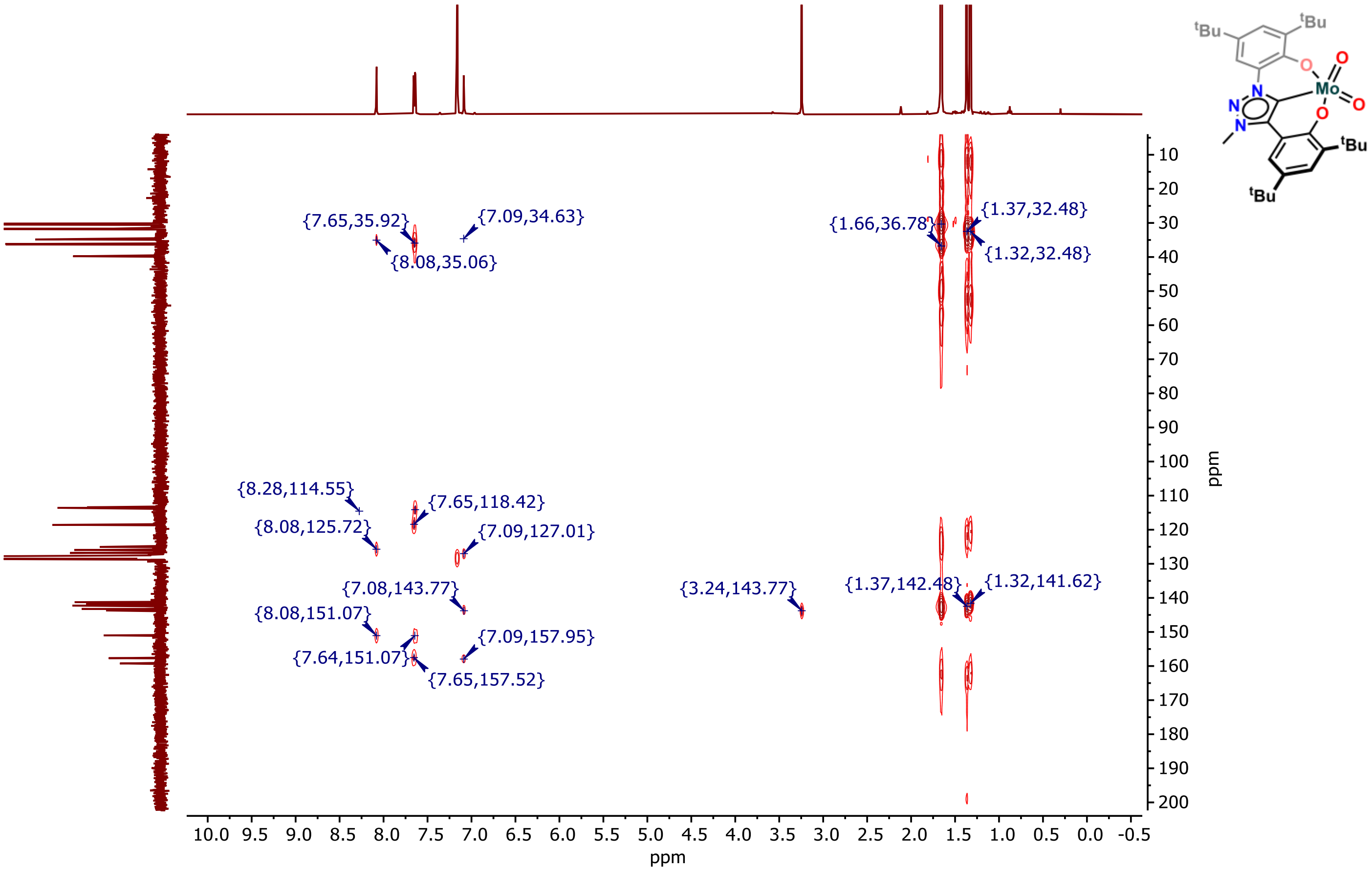

Figure S 5: ^{1}H-^{13}C HMBC spectrum of **2-Mo** in benzene-d_6 at 298 K.

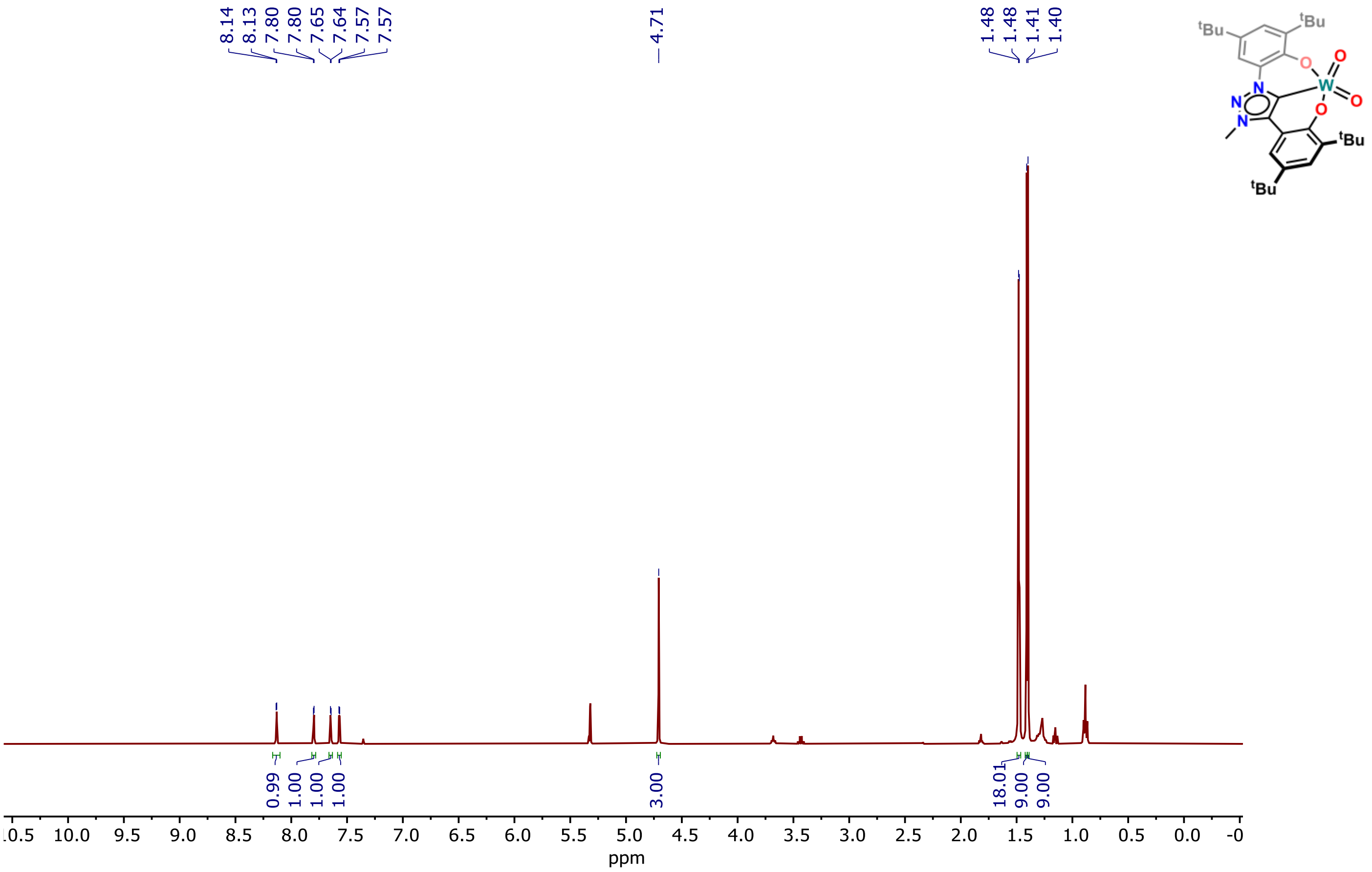

Figure S 6: ¹H-NMR spectrum of **2-W** in dichloromethane-d_2 at 298 K.

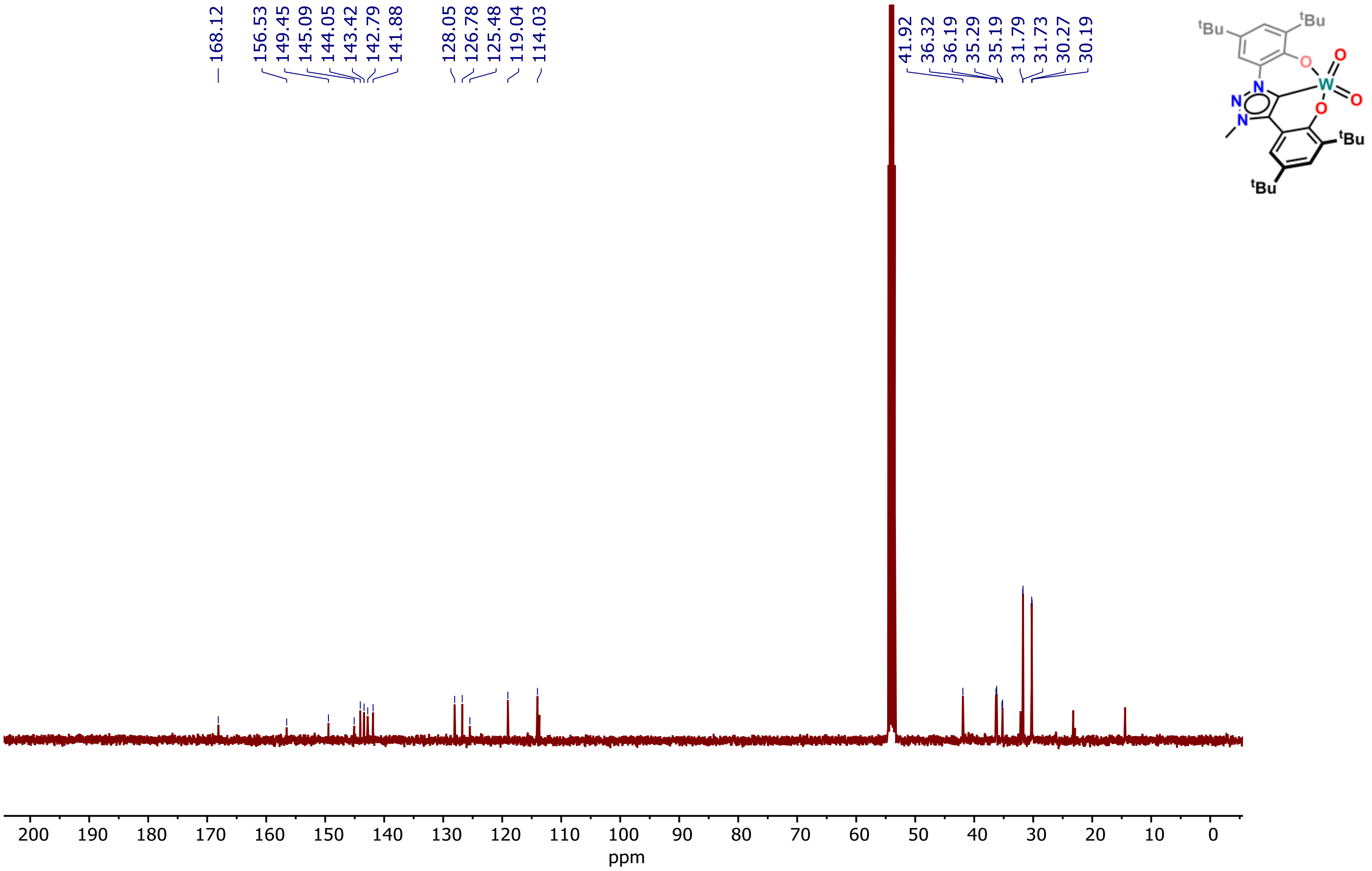

Figure S 7: $^{13}C\{^{1}H\}$-NMR spectrum of **2-W** in dichloromethane-d_2 at 298 K.

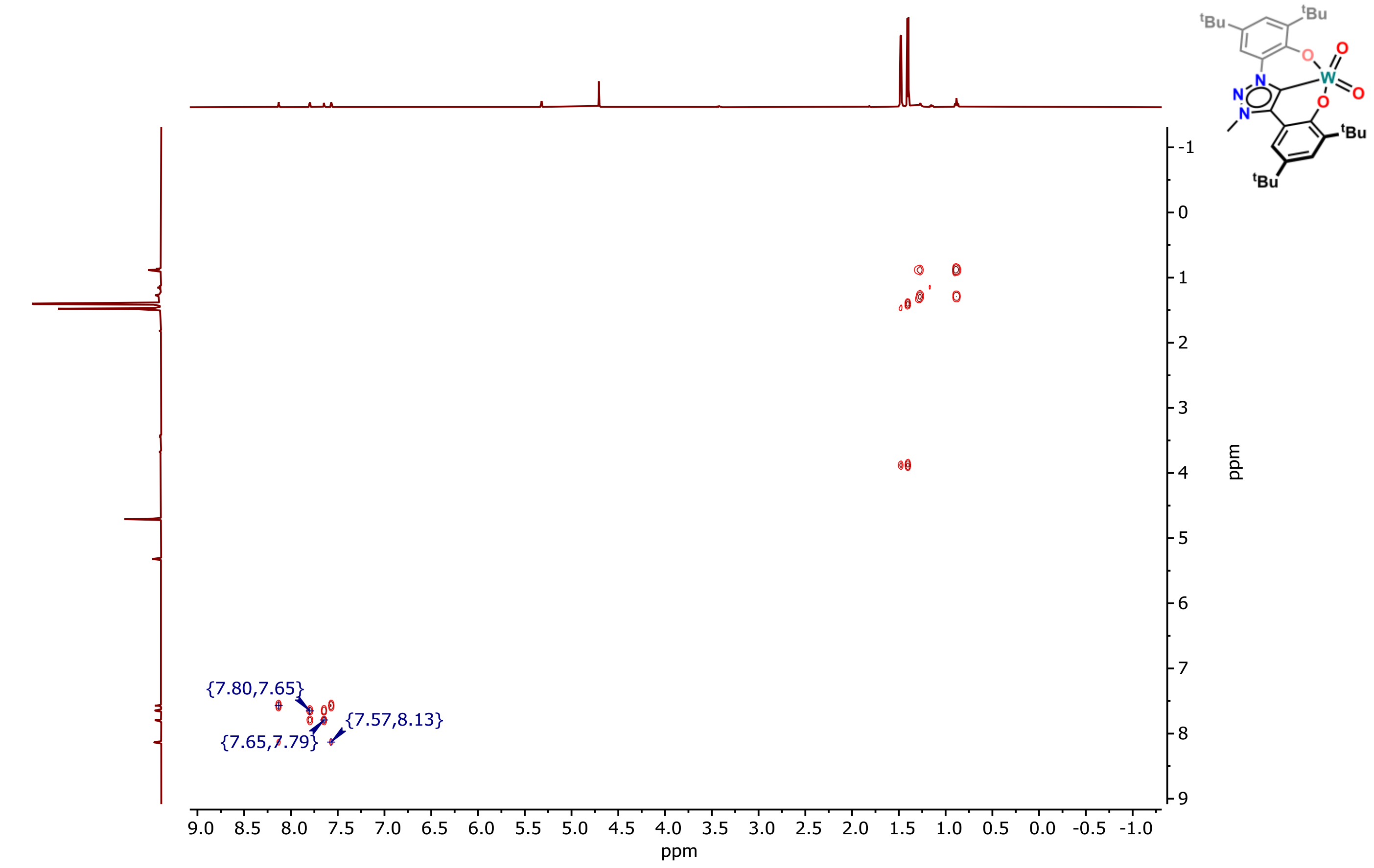

Figure S 8: ^{1}H-^{1}H-COSY spectrum of **2-W** in dichloromethane-d_2 at 298 K.

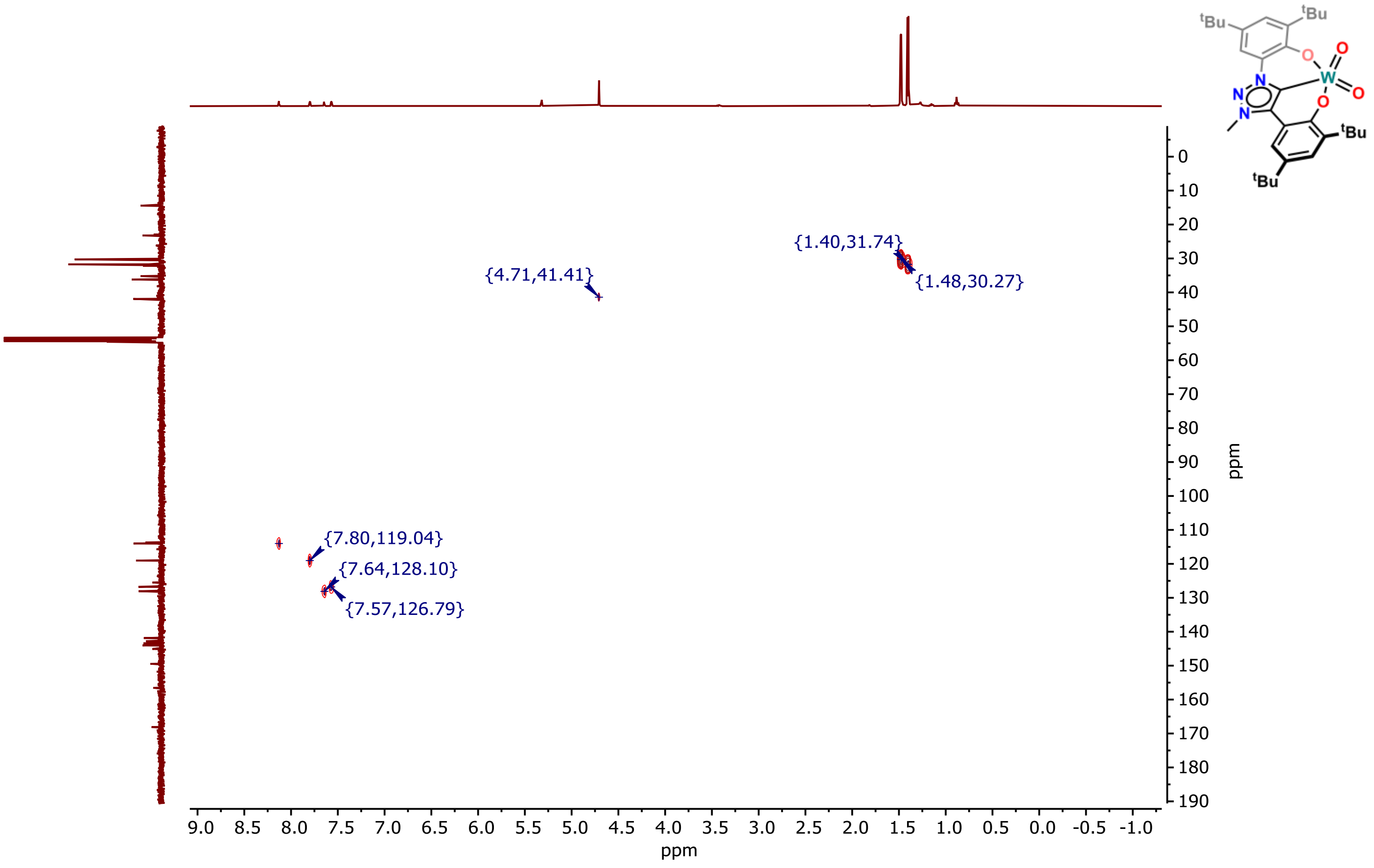

Figure S 9: ^{1}H-^{13}C HSQC spectrum of **2-W** in dichloromethane-d_2 at 298 K.

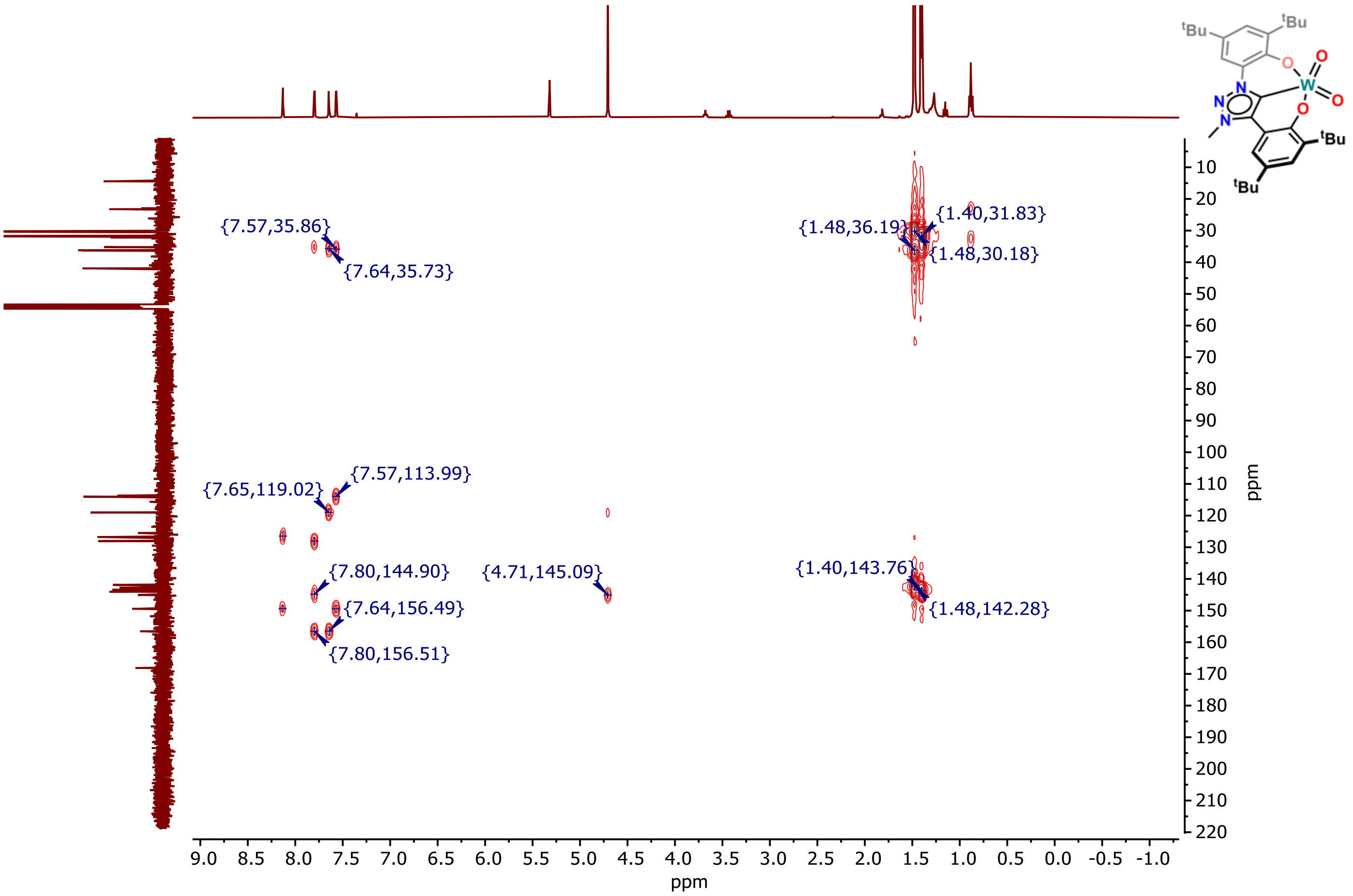

Figure S 10: ^{1}H-^{13}C HMBC spectrum of **2-W** in dichloromethane-d_2 at 298 K.

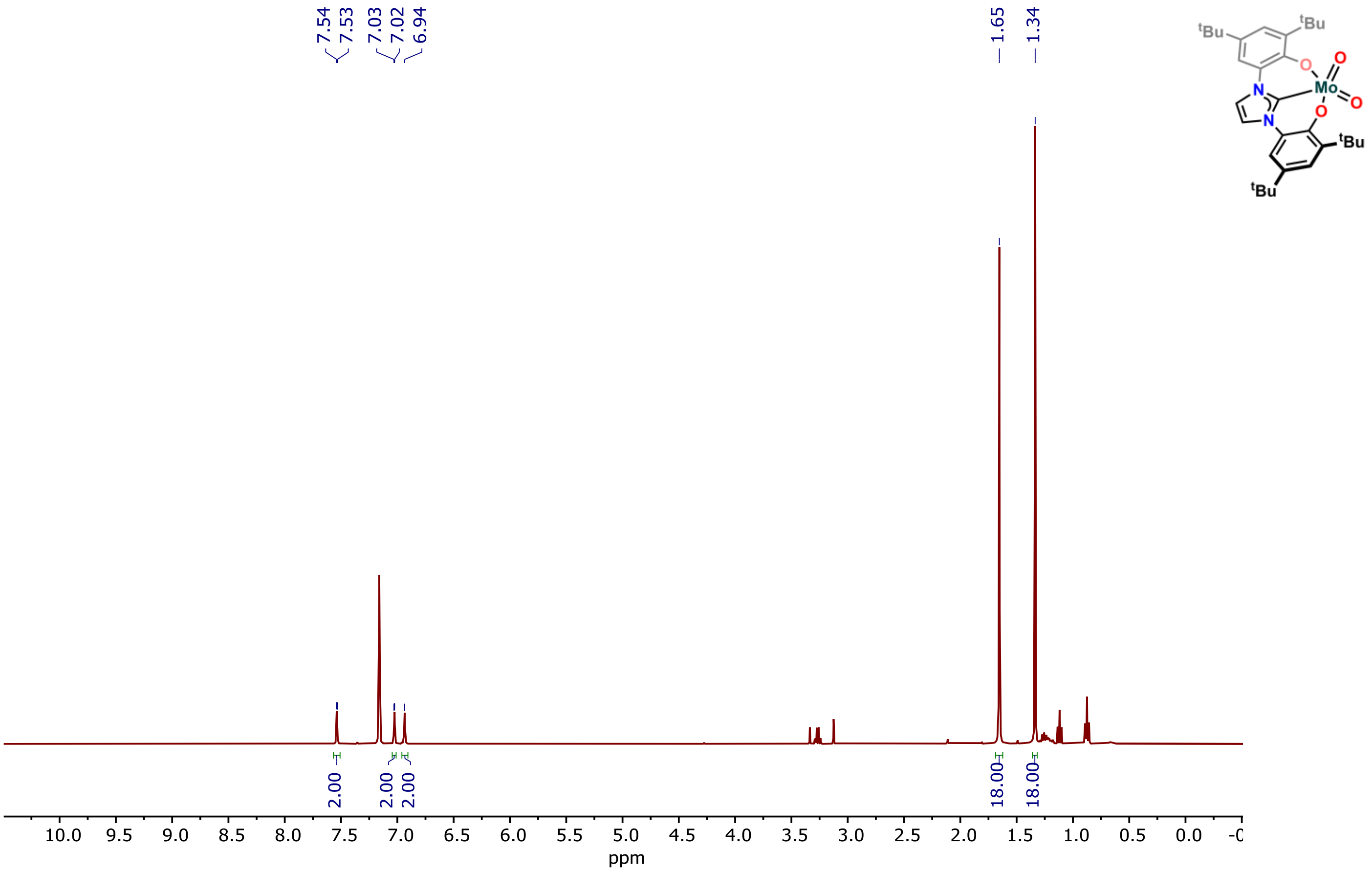

Figure S 11: ^{1}H-NMR spectrum of **3-Mo** in benzene-d_6 at 298 K.

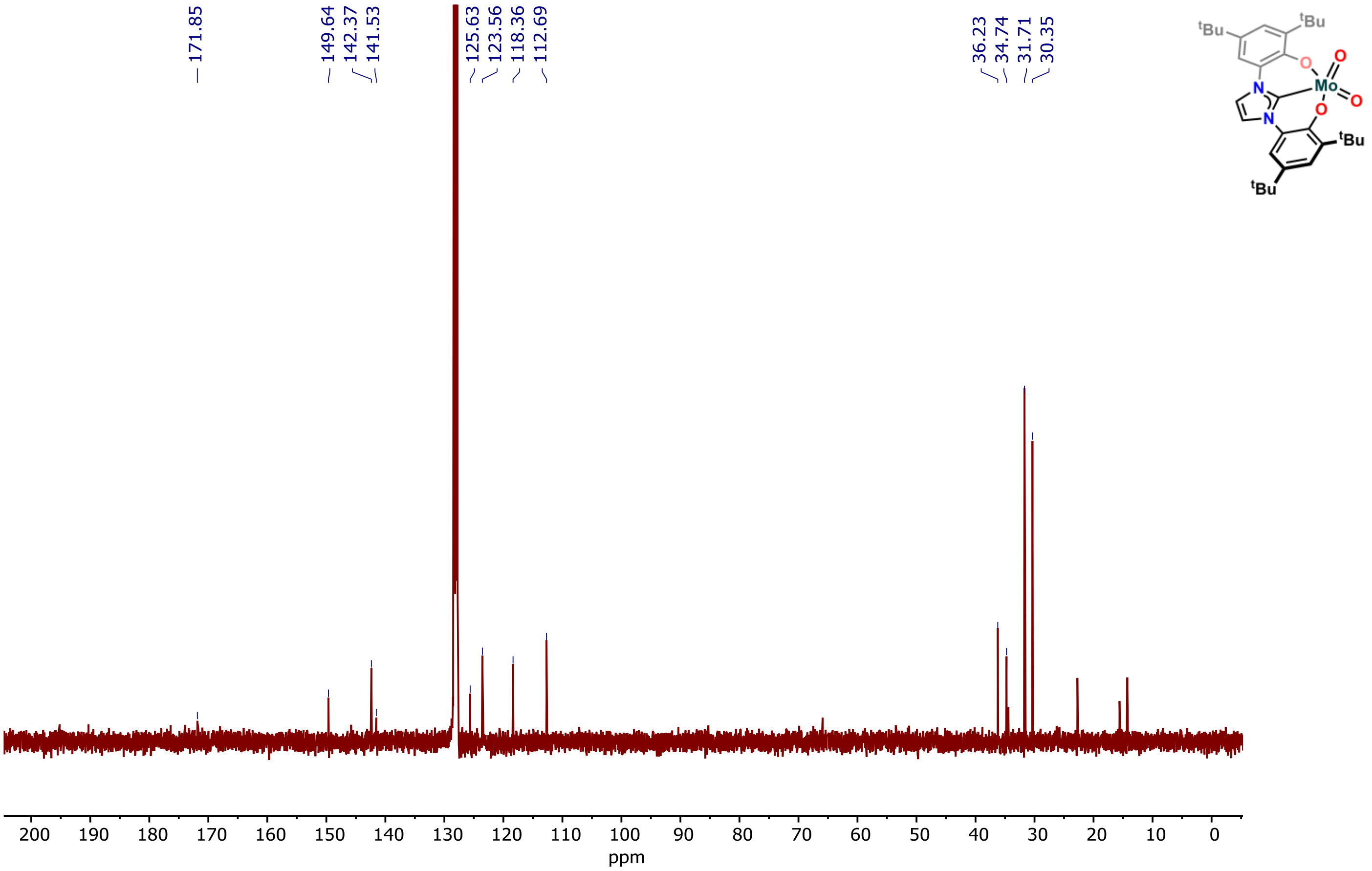

Figure S 12: ^{13}C{^{1}H}-NMR spectrum of **3-Mo** in benzene-d_6 at 298 K.

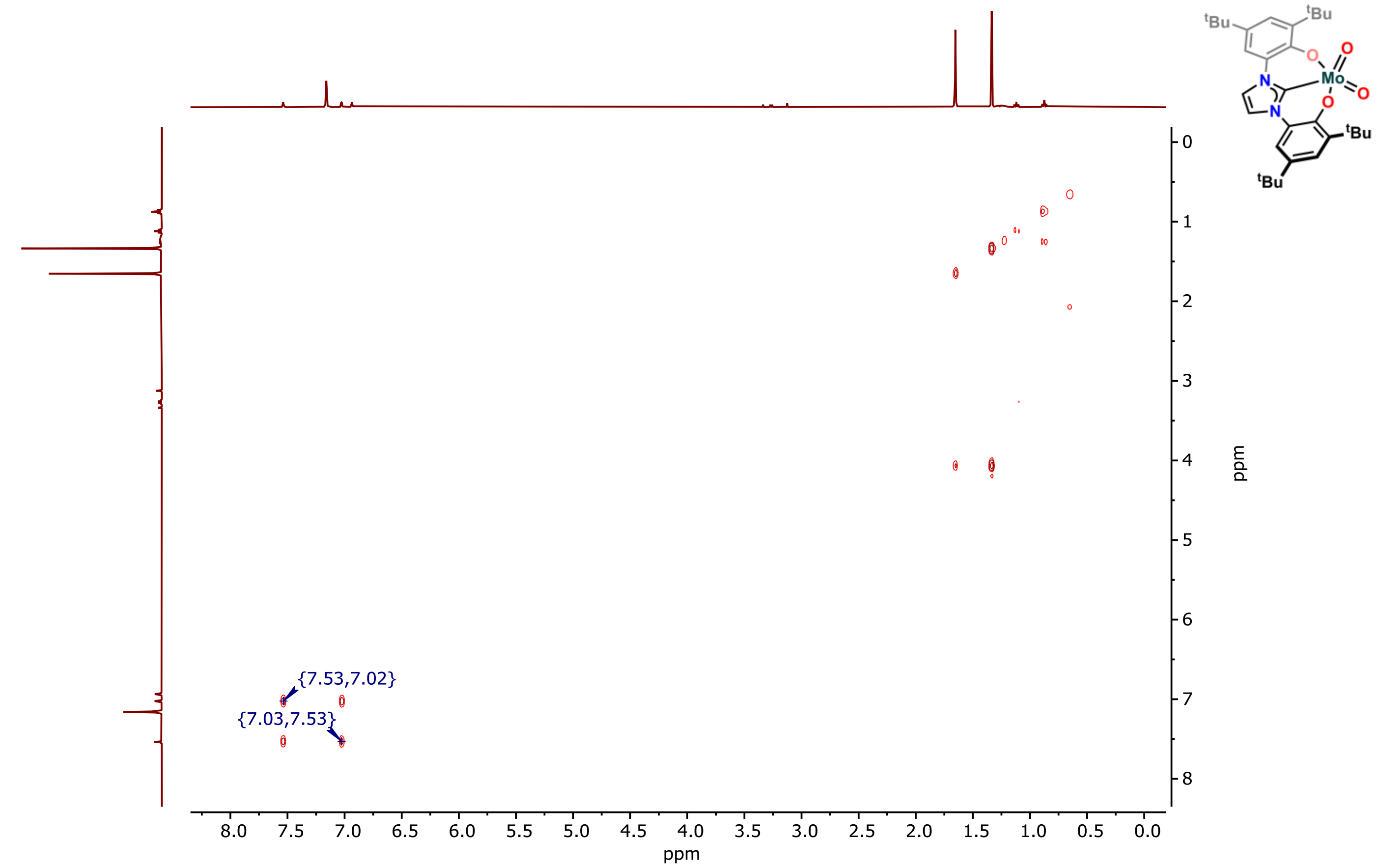

Figure S 13: ^{1}H-^{1}H COSY of **3-Mo** in benzene-d_6 at 298 K.

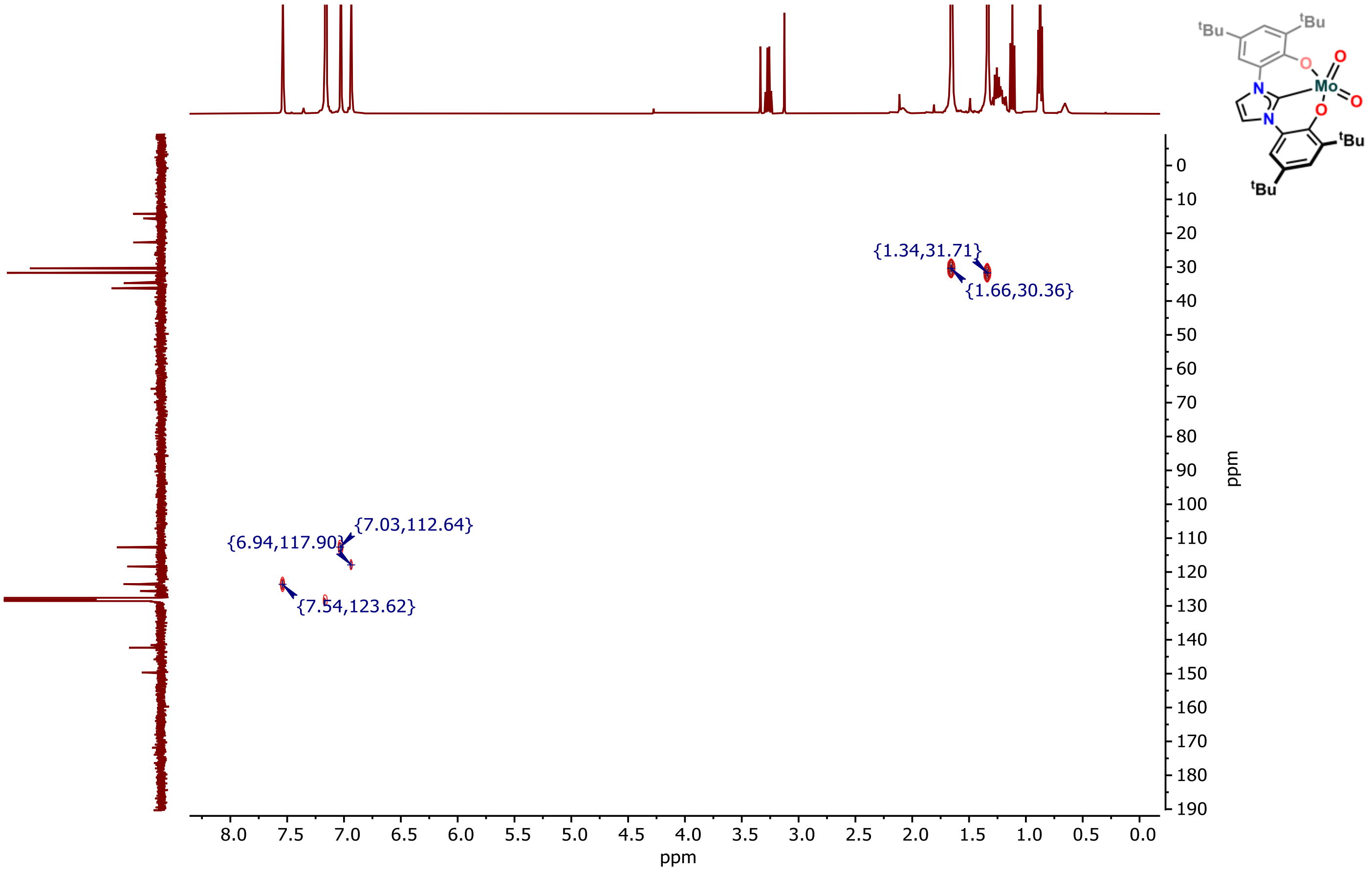

Figure S 14: ^{1}H-^{13}C HSQC of **3-Mo** in benzene-d_6 at 298 K.

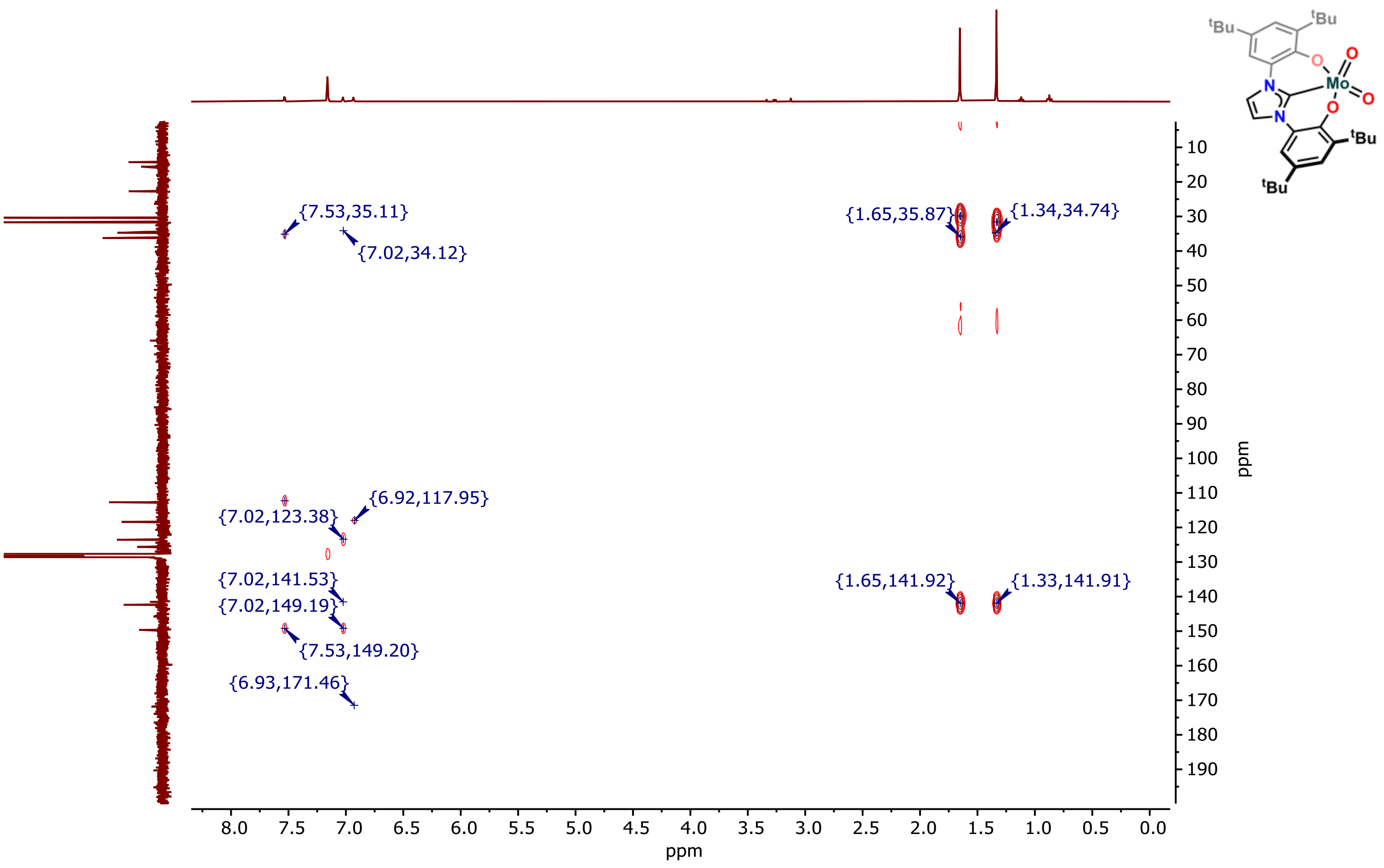

Figure S 15: ^{1}H-^{13}C HMBC spectrum of **3-Mo** in benzene-d_6 at 298 K.

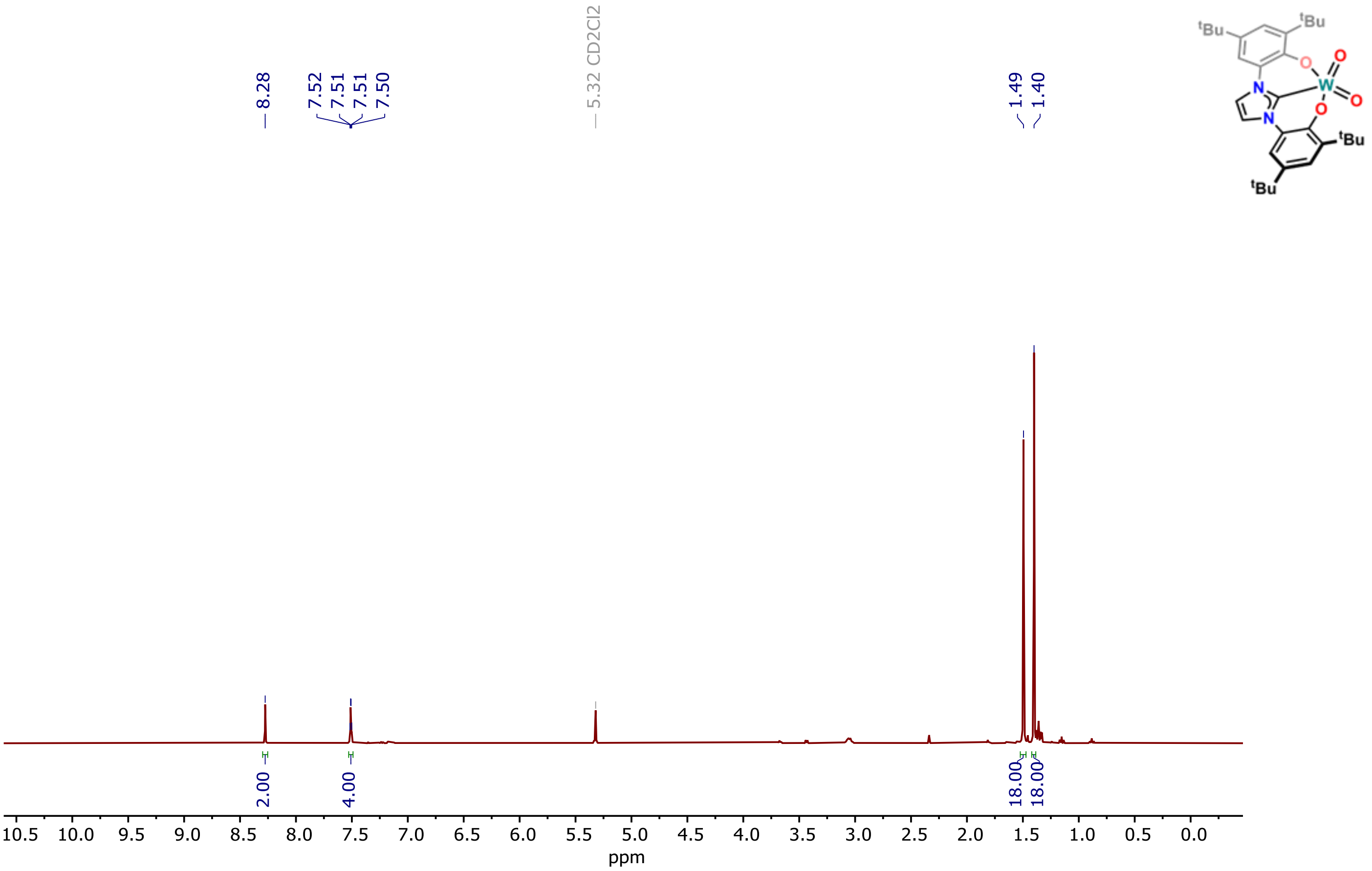

Figure S 16: ^{1}H-NMR spectrum of **3-W** in dichloromethane-d_2 at 298 K.

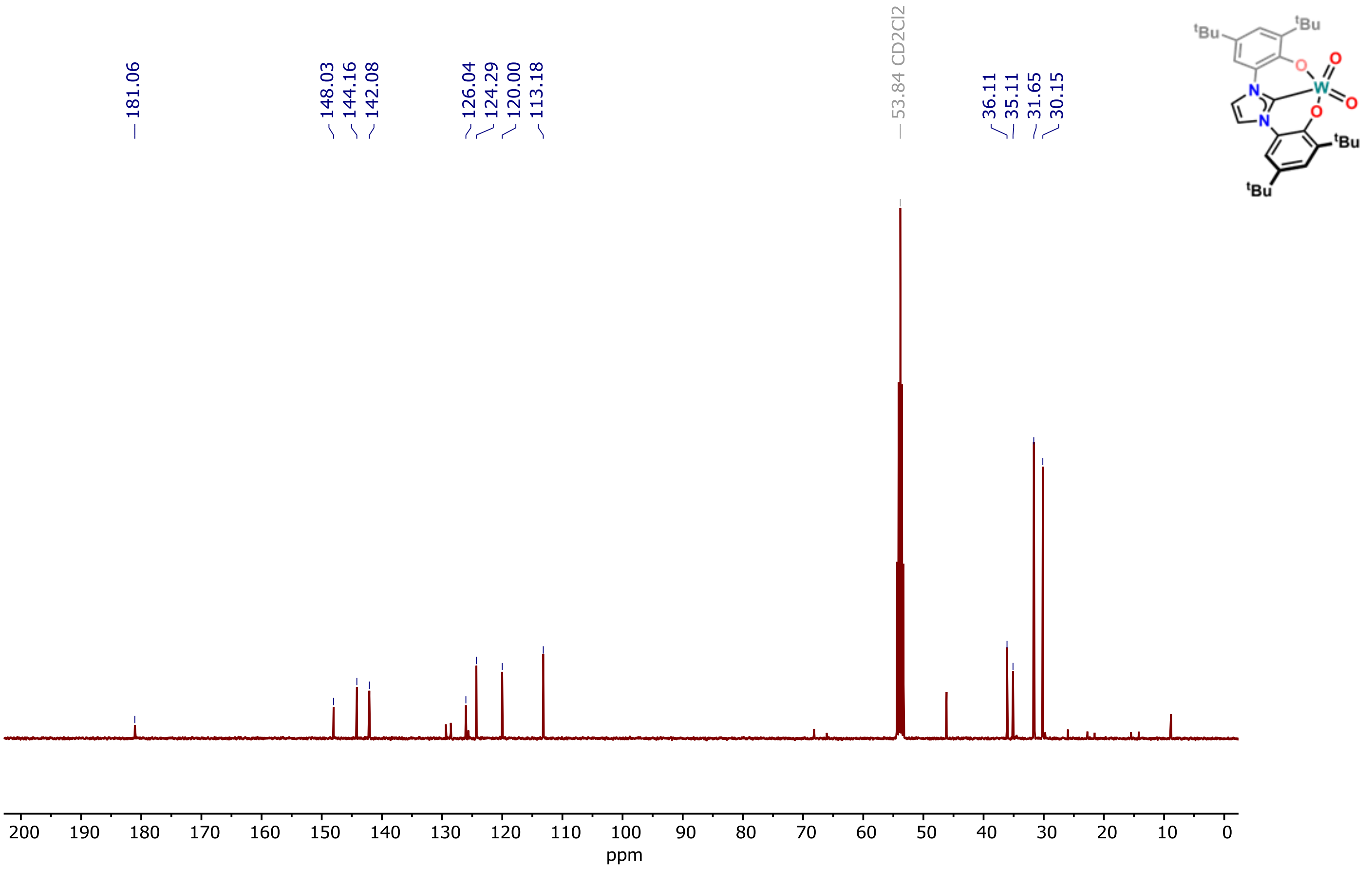

Figure S 17: $^{13}C\{^{1}H\}$-NMR spectrum of **3-W** in dichloromethane-d_2 at 298 K.

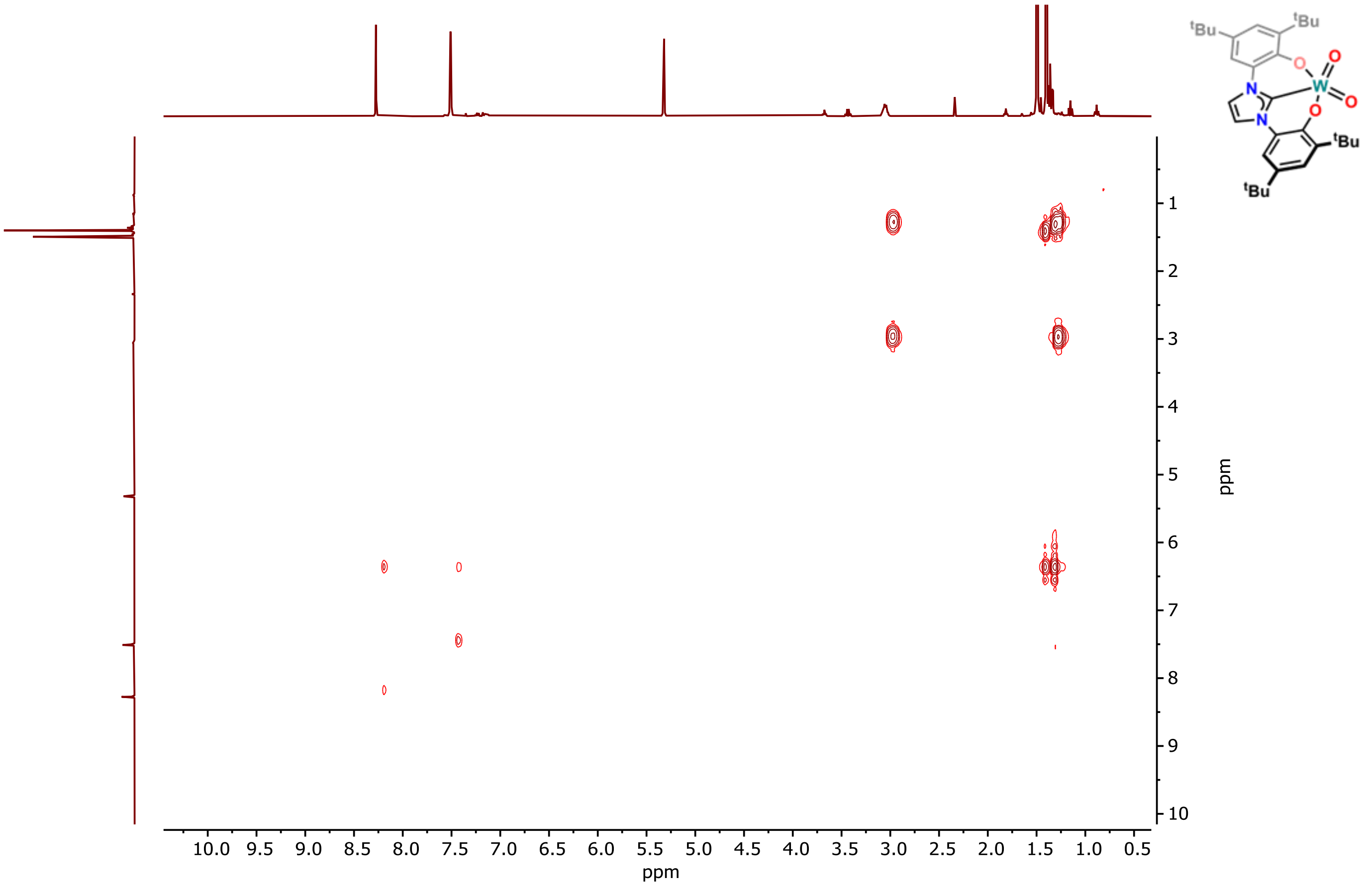

Figure S 18: ^{1}H-^{1}H COSY spectrum of **3-W** in dichloromethane-d_2 at 298 K.

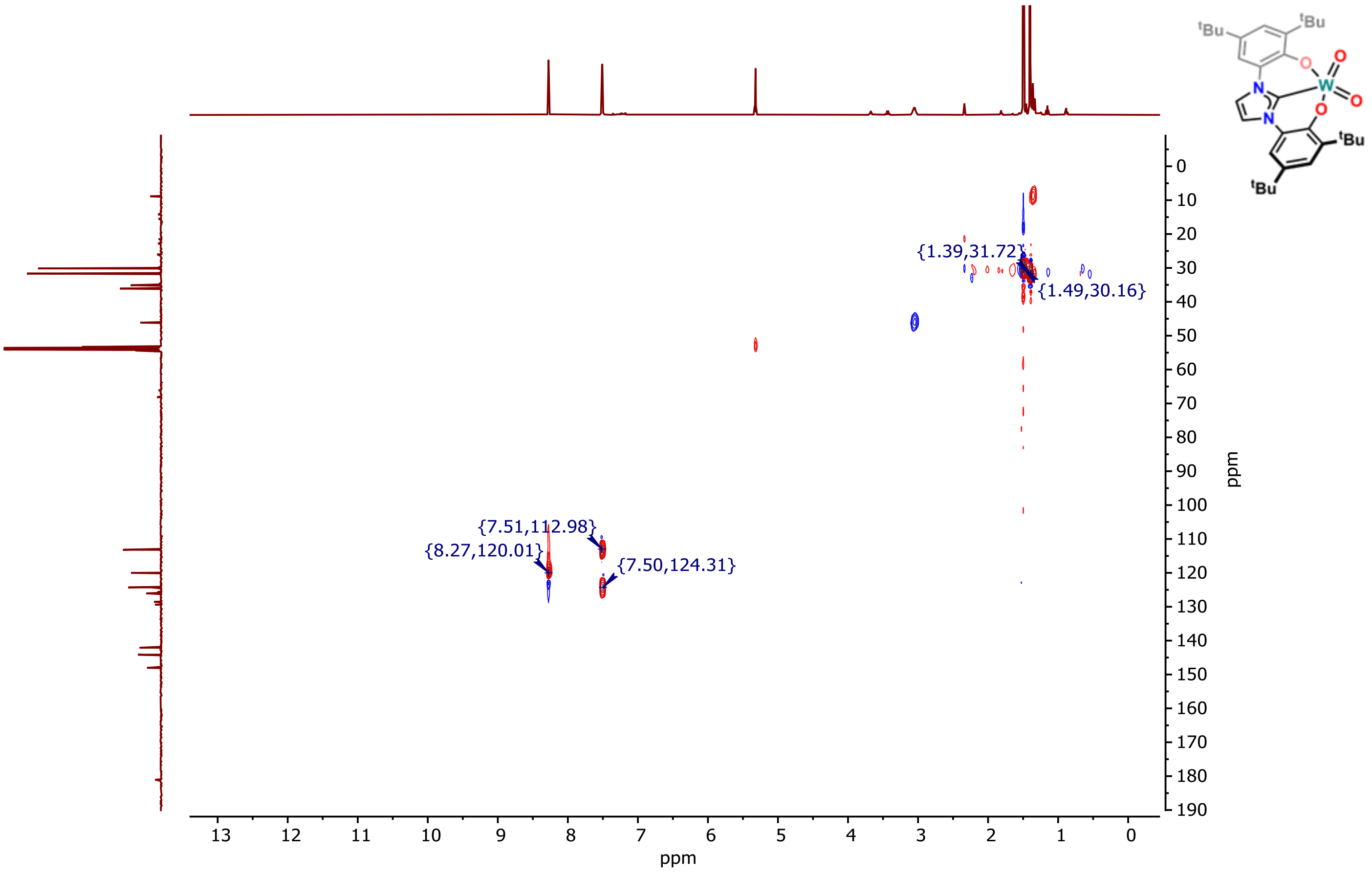

Figure S 19: ^{1}H-^{13}C HSQC spectrum of **3-W** in dichloromethane-d_2 at 298 K.

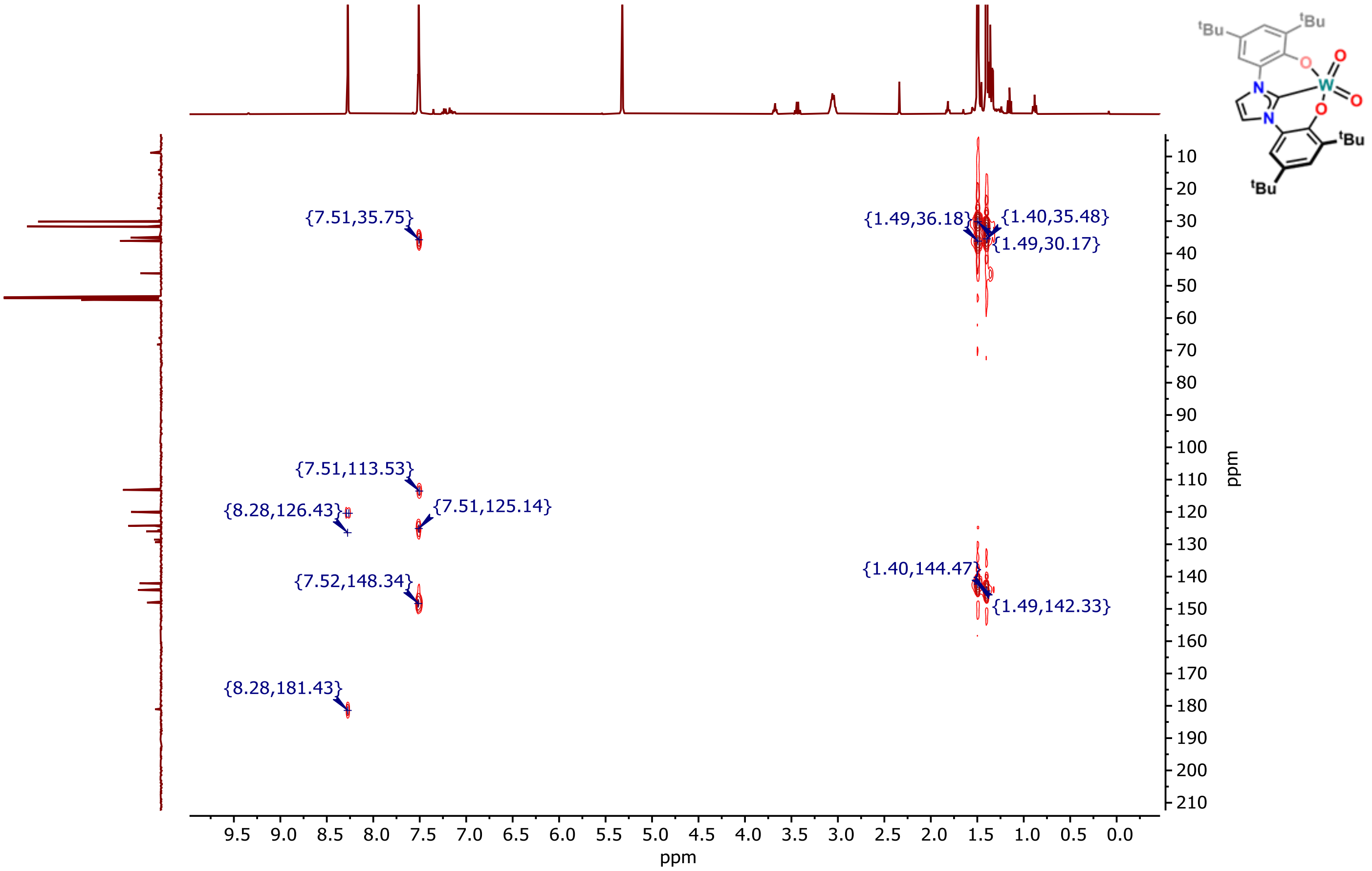

Figure S 20: ^{1}H-^{13}C HMBC spectrum of **3-W** in dichloromethane-d_2 at 298 K.

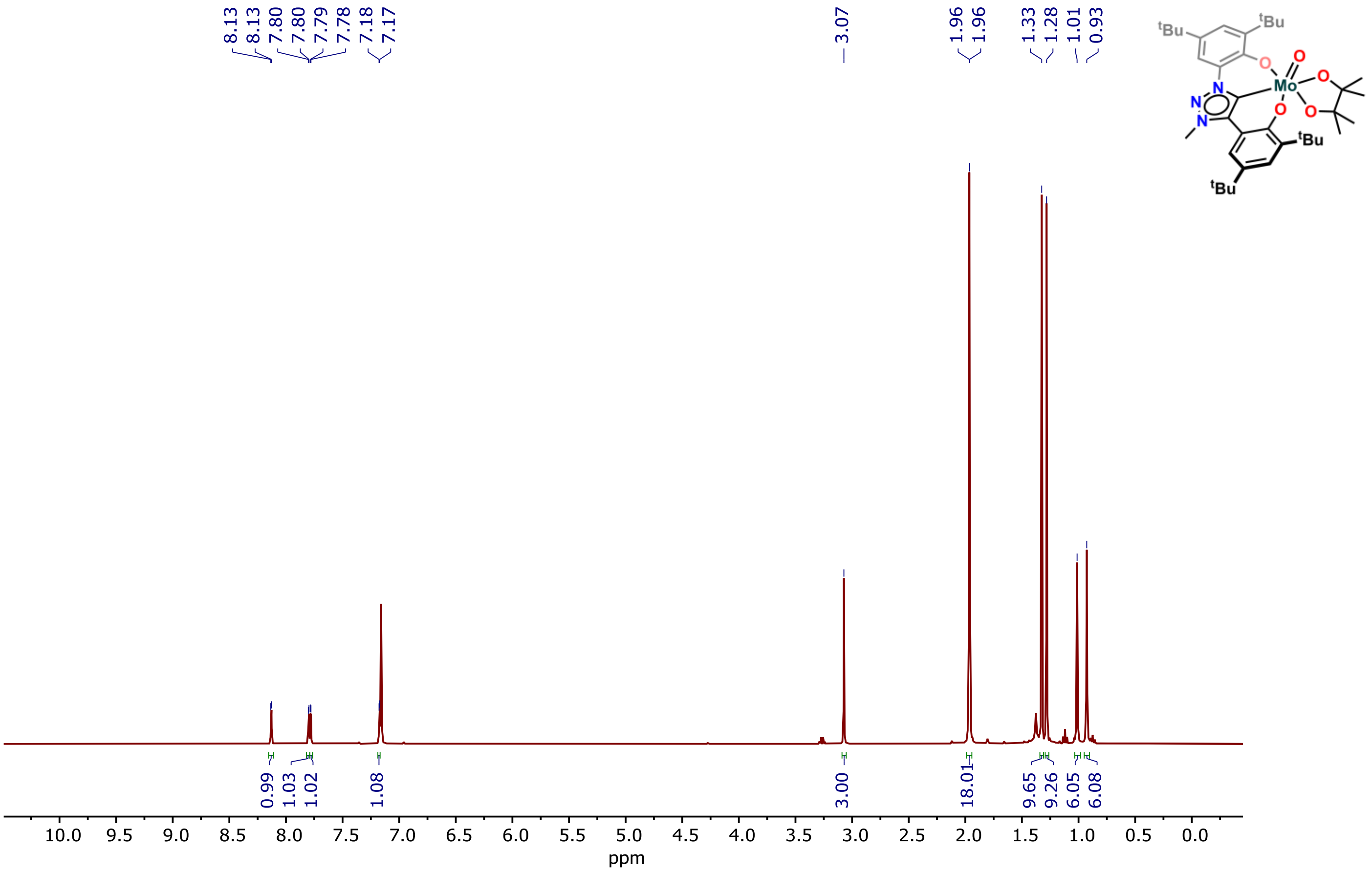

Figure S 21:^{1}H-NMR spectrum of **5-Mo** in benzene-d_6 at 298 K.

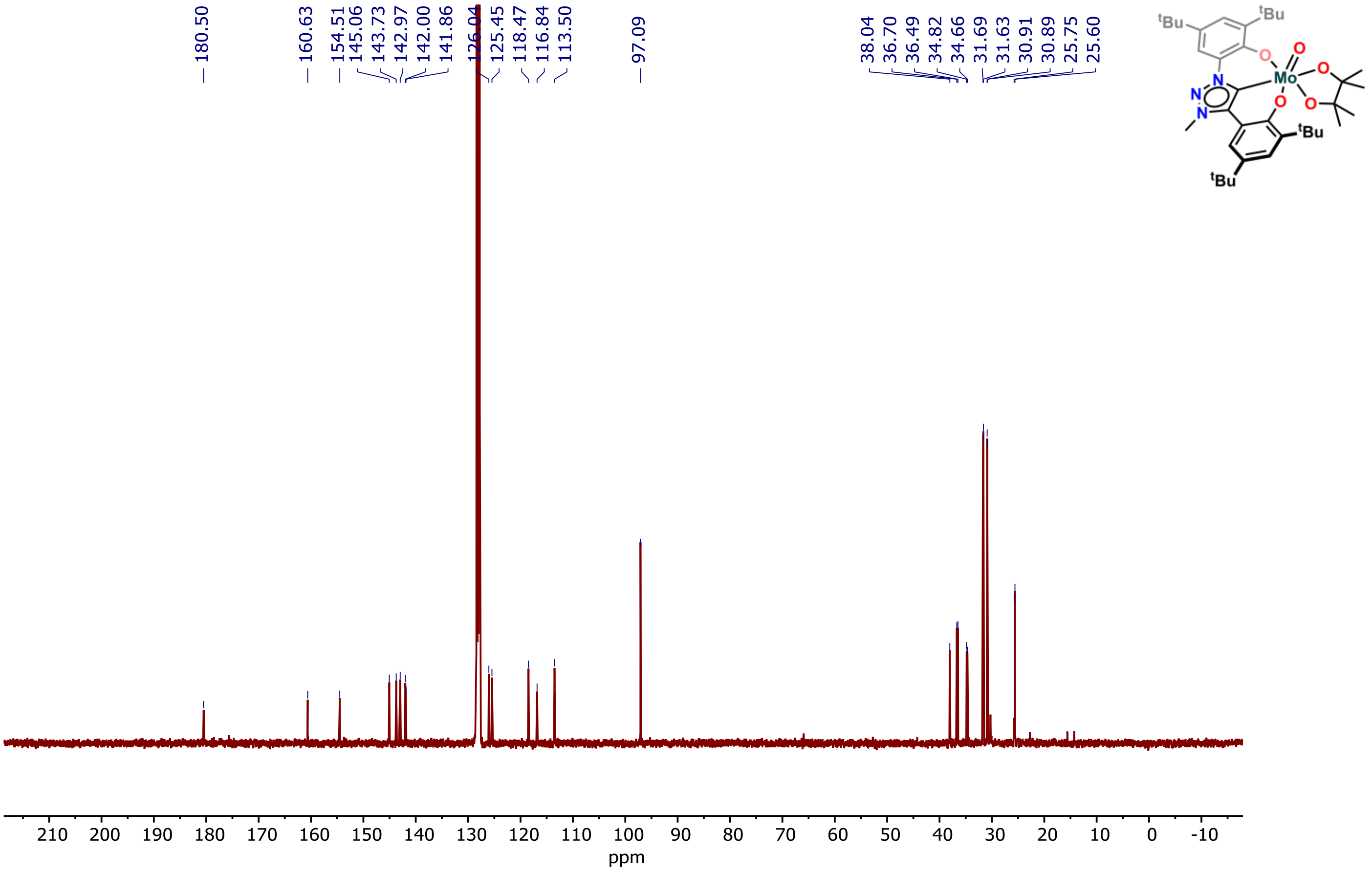

Figure S 22: ^{13}C{^{1}H}-NMR spectrum of **5-Mo** in benzene-d_6 at 298 K.

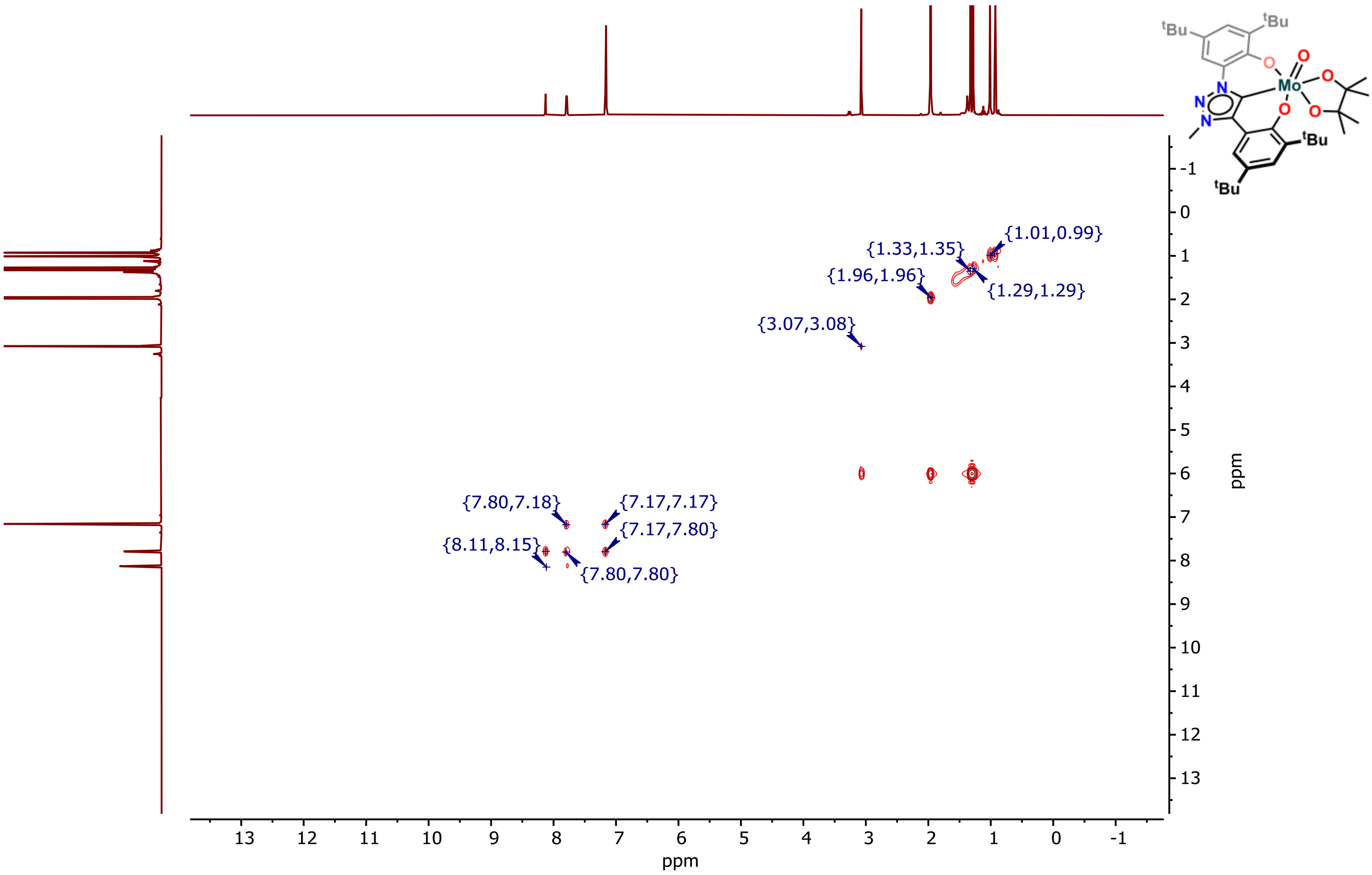

Figure S 23: ^1H-^1H COSY spectrum of **5-Mo** in benzene-d_6 at 298 K.

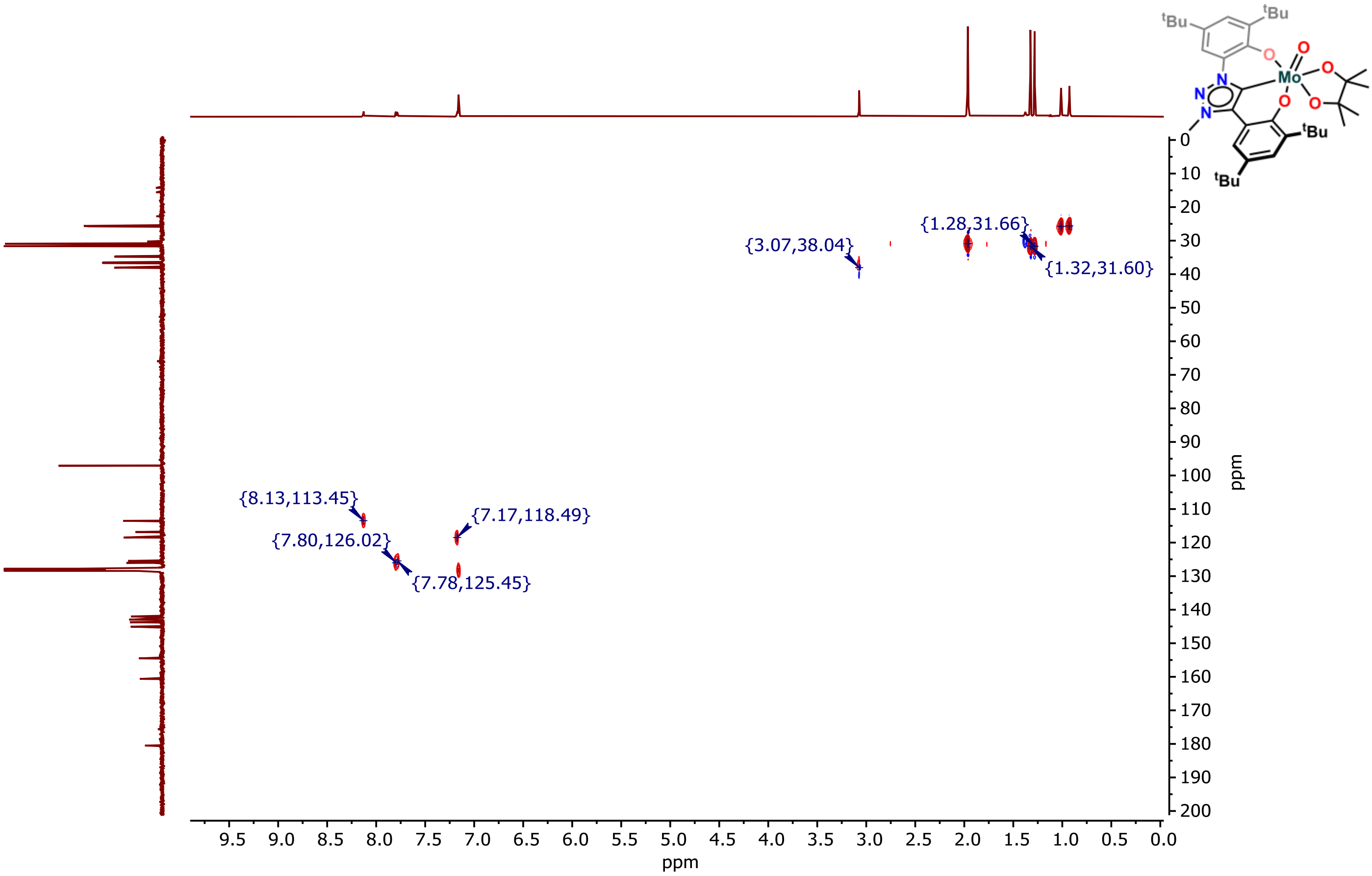

Figure S 24: ^1H-^{13}C HSQC spectrum of **5-Mo** in benzene-d_6 at 298 K.

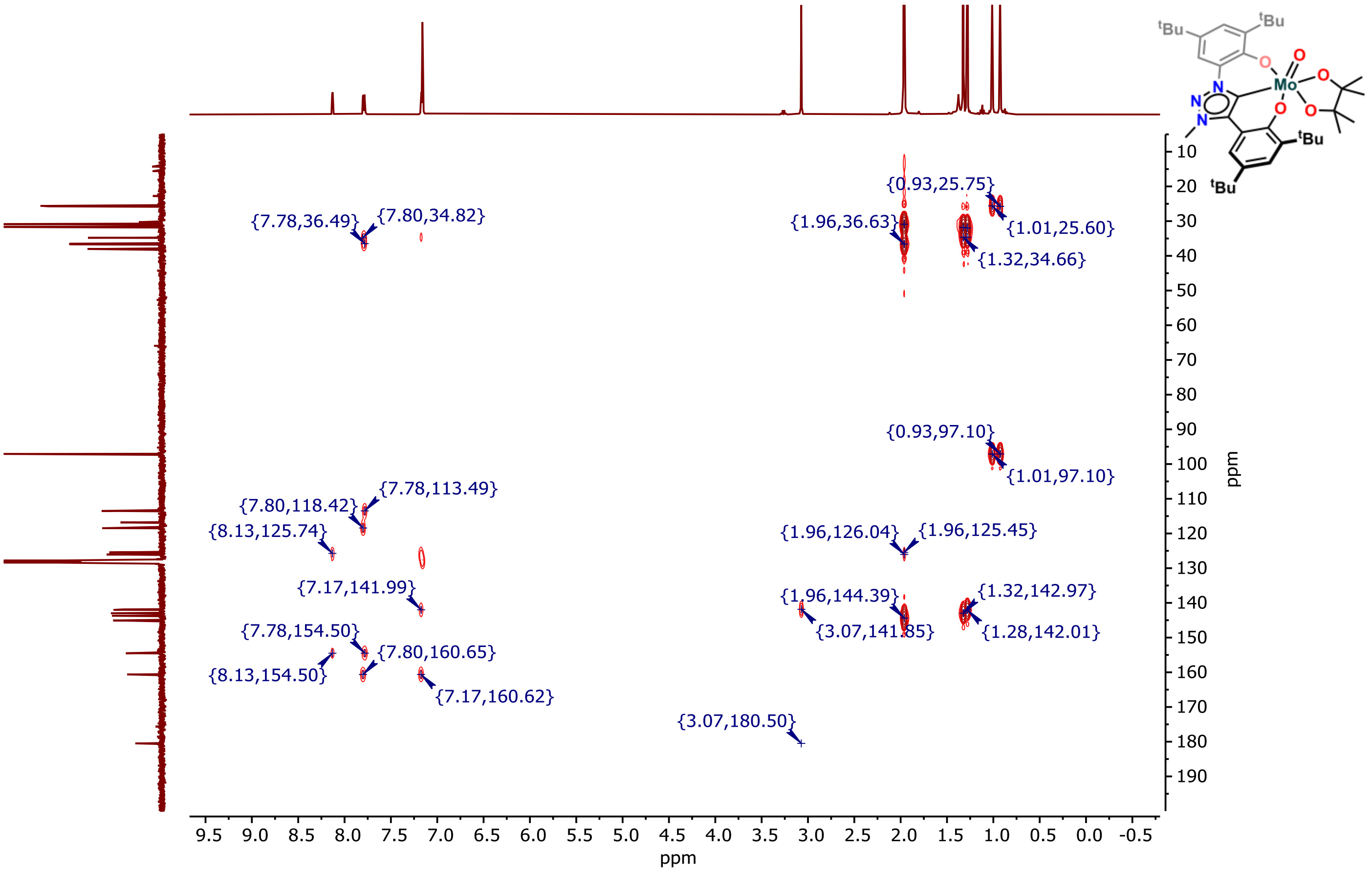

Figure S 25: ^{1}H-^{13}C HMBC spectrum of **5-Mo** in benzene-d_6 at 298 K.

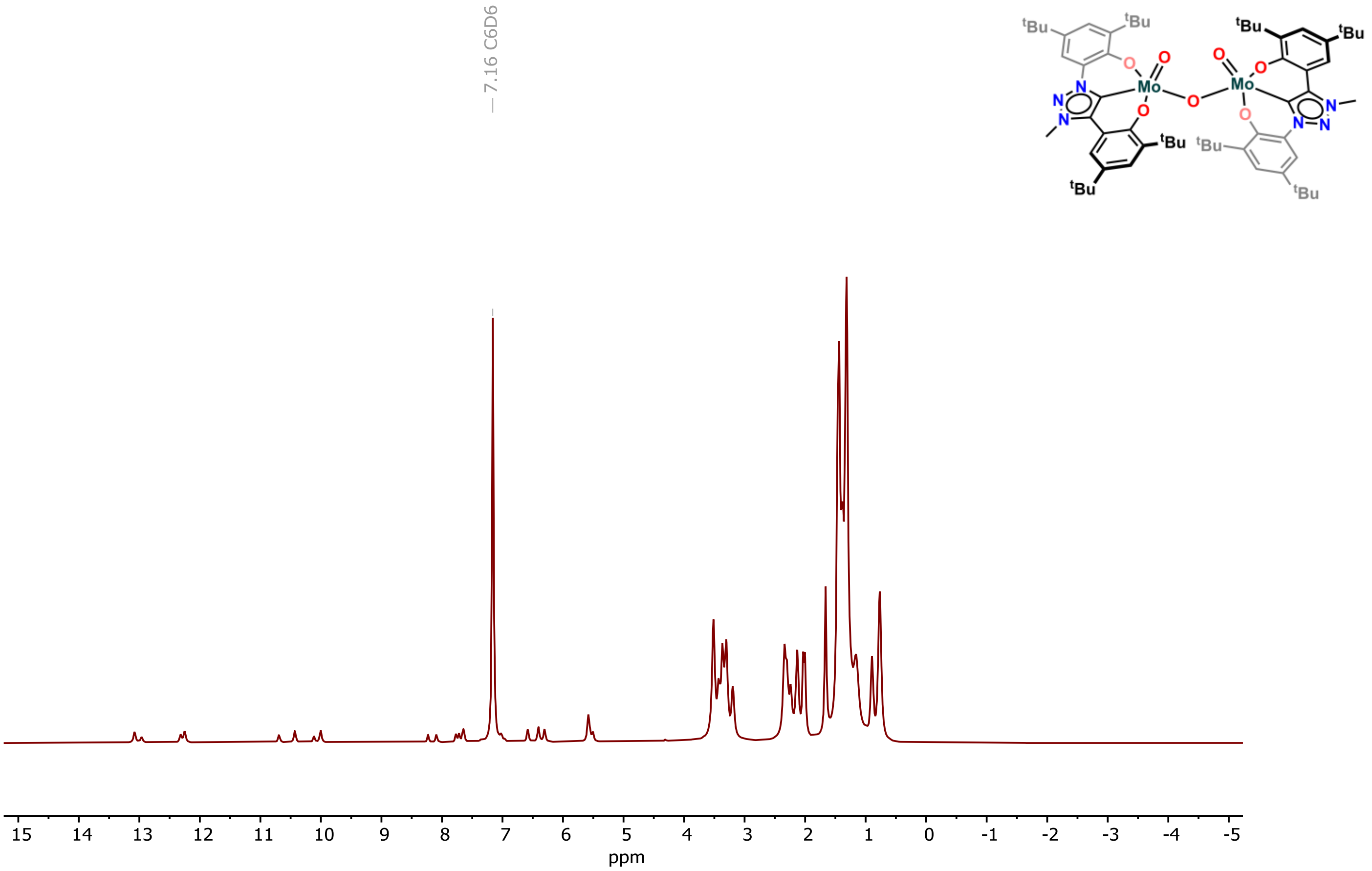

Figure S 26: ^{1}H-NMR spectrum of **7-Mo** in benzene-d_6 at 298K.

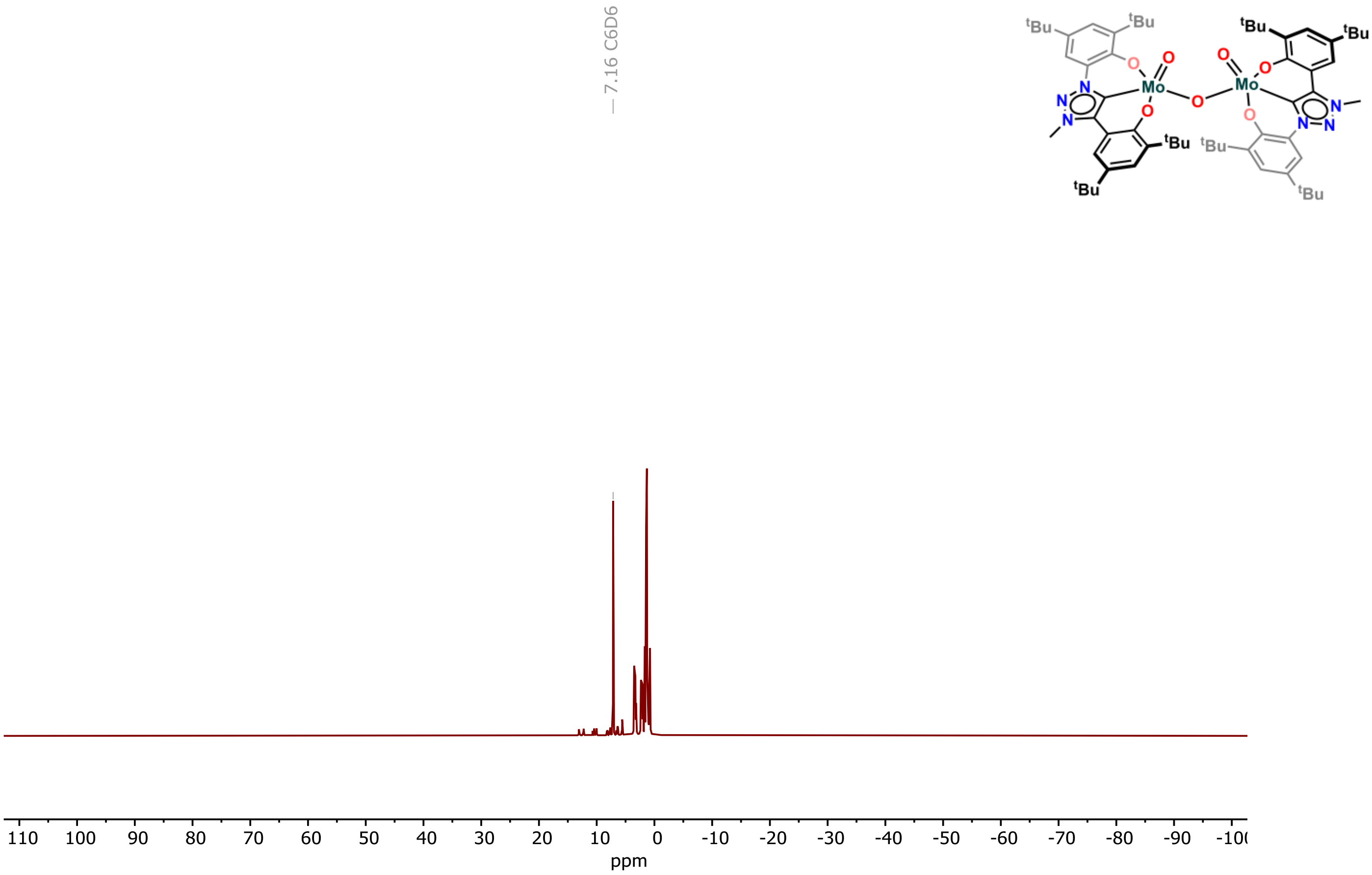

Figure S 27: ^{1}H-NMR spectrum of **7-Mo** in benzene-d_6 at 298K.

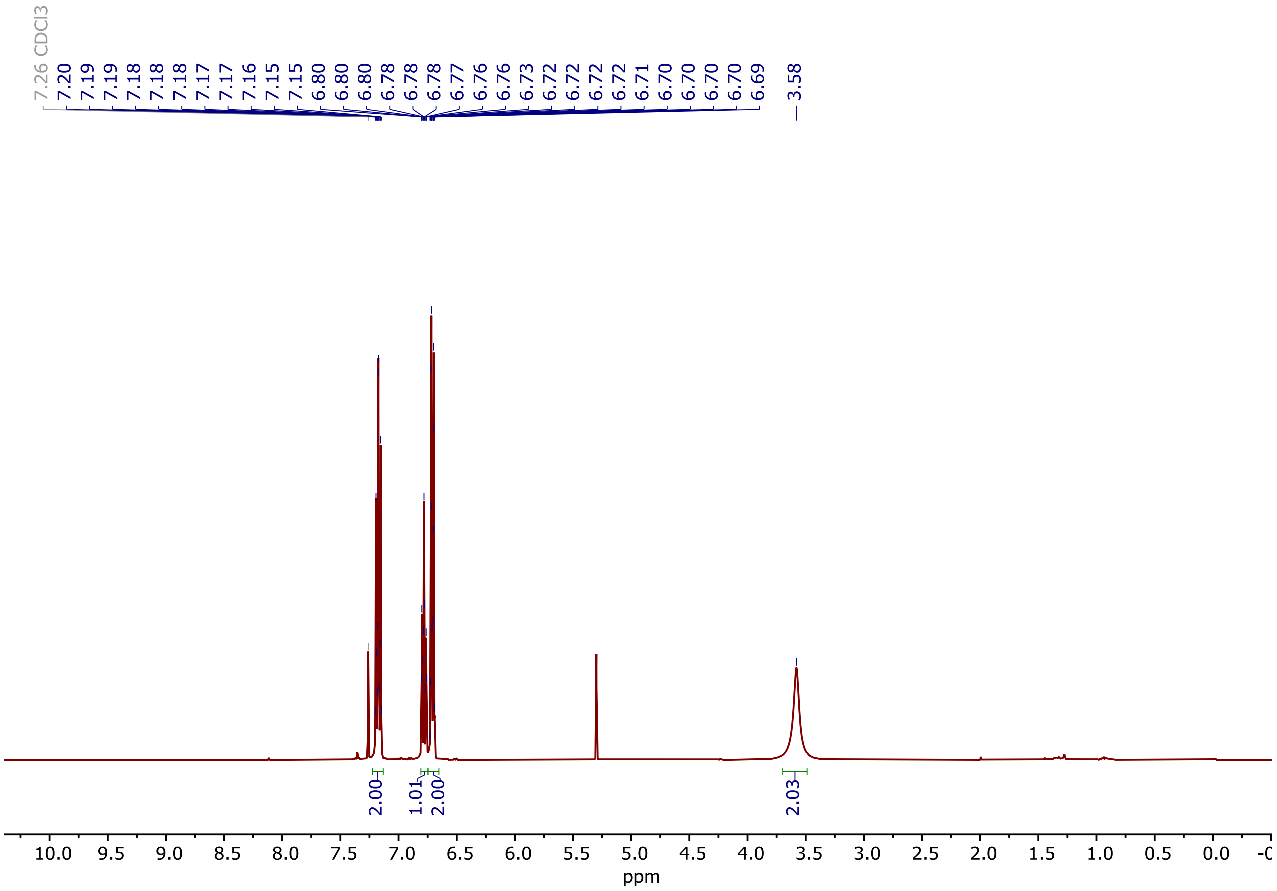

Figure S 28: ^{1}H-NMR spectrum of aniline in chloroform-d at 298 K.

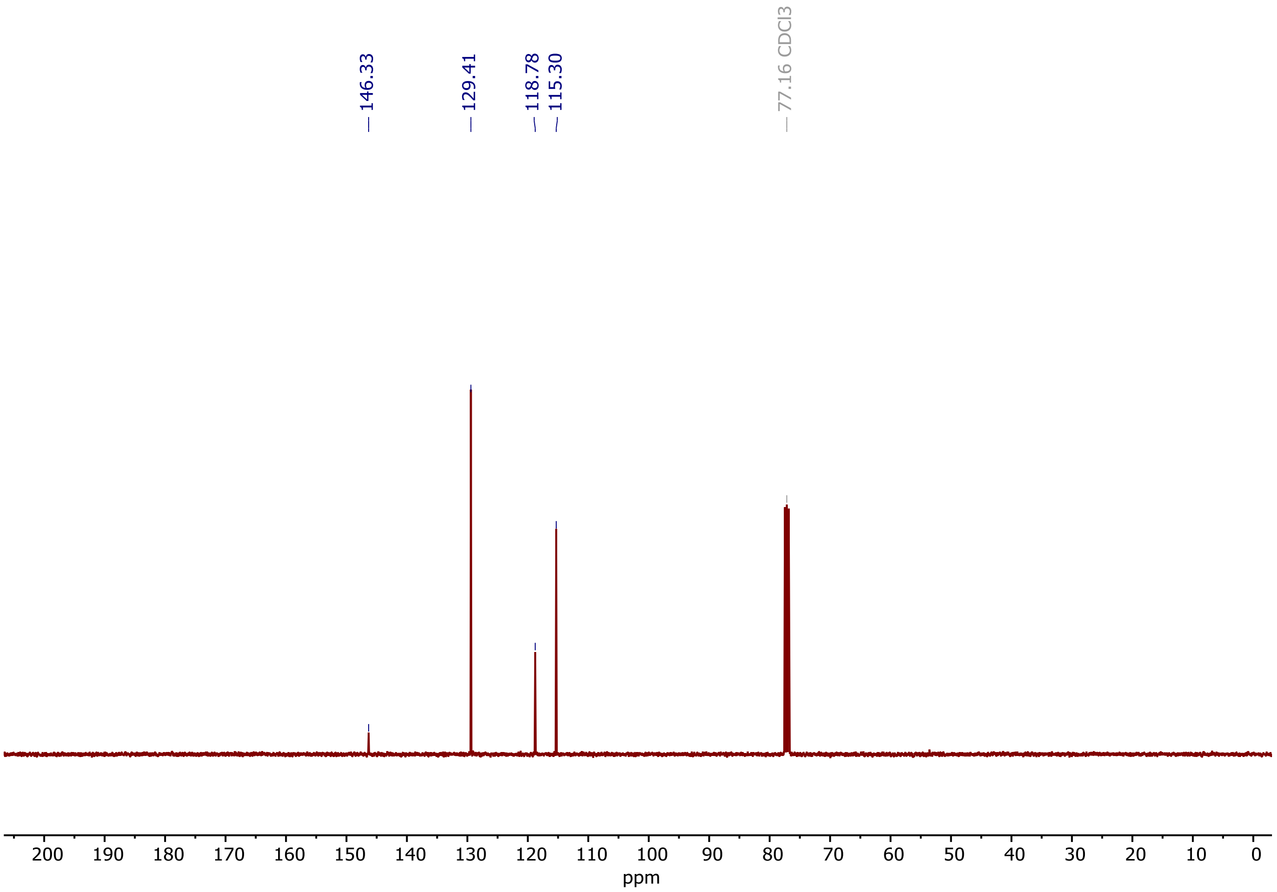

Figure S 29: $^{13}C\{^{1}H\}$-NMR spectrum of aniline in chloroform-*d* at 298 K.

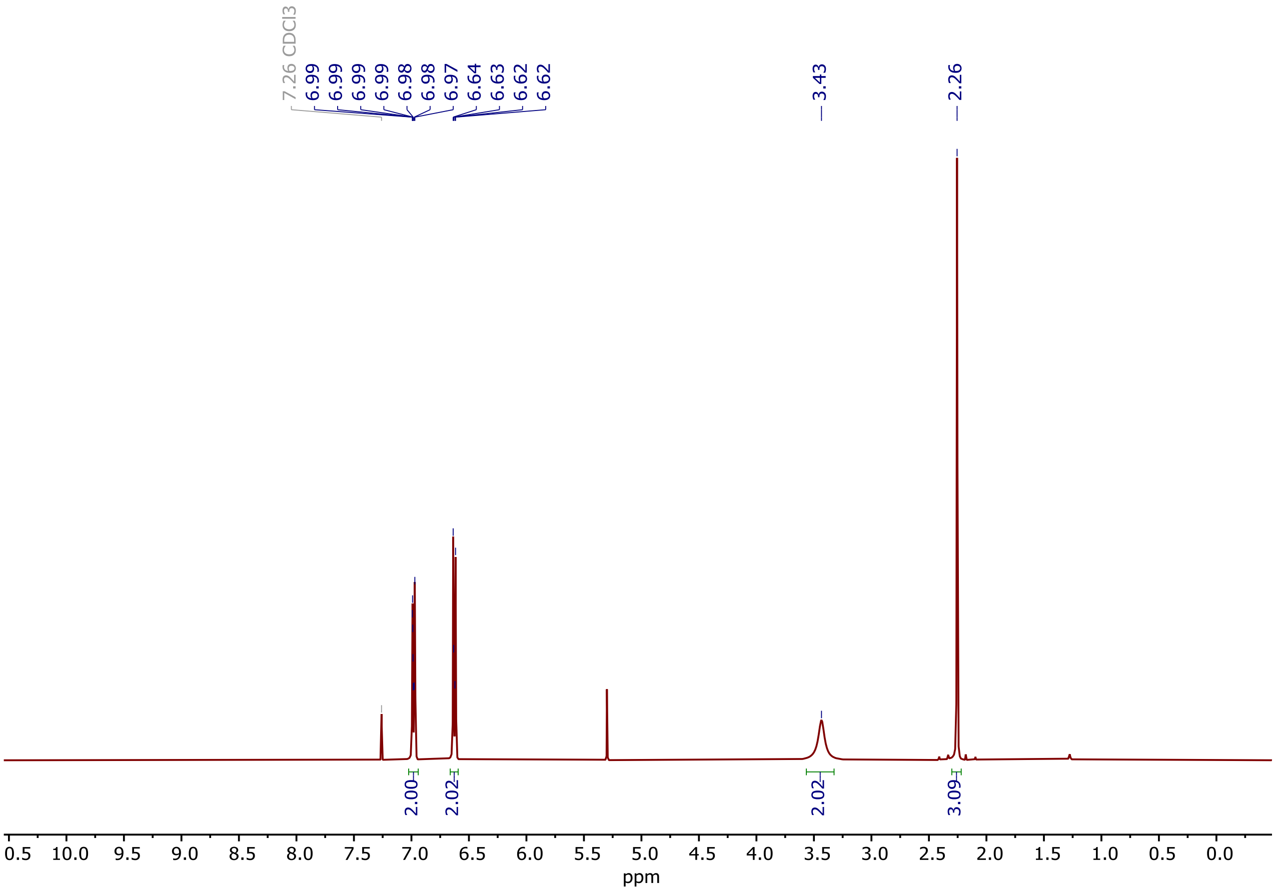

Figure S 30: ^{1}H-NMR spectrum of 4-methylaniline in chloroform-*d* at 298 K.

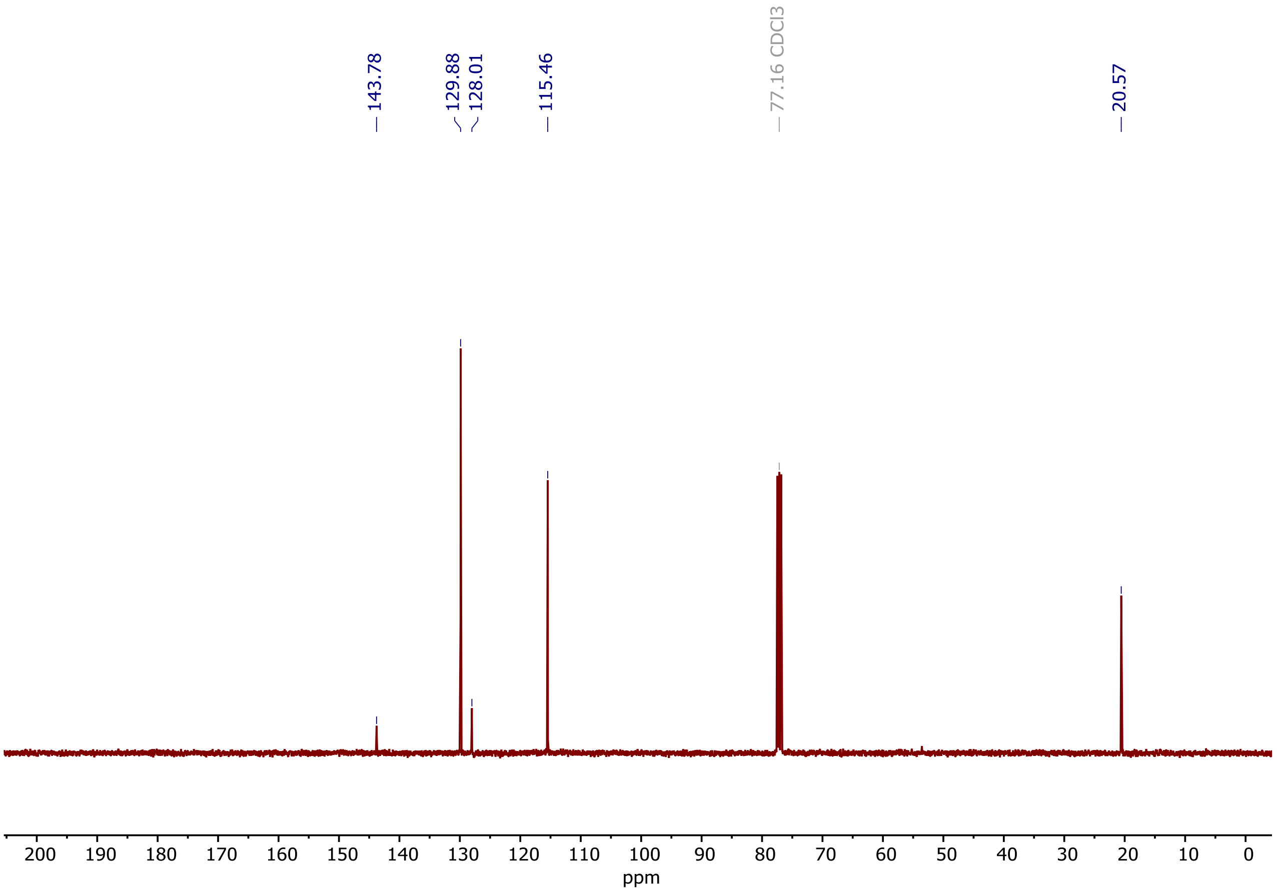

Figure S 31: $^{13}C\{^{1}H\}$-NMR spectrum of 4-methylaniline in chloroform-*d* at 298 K.

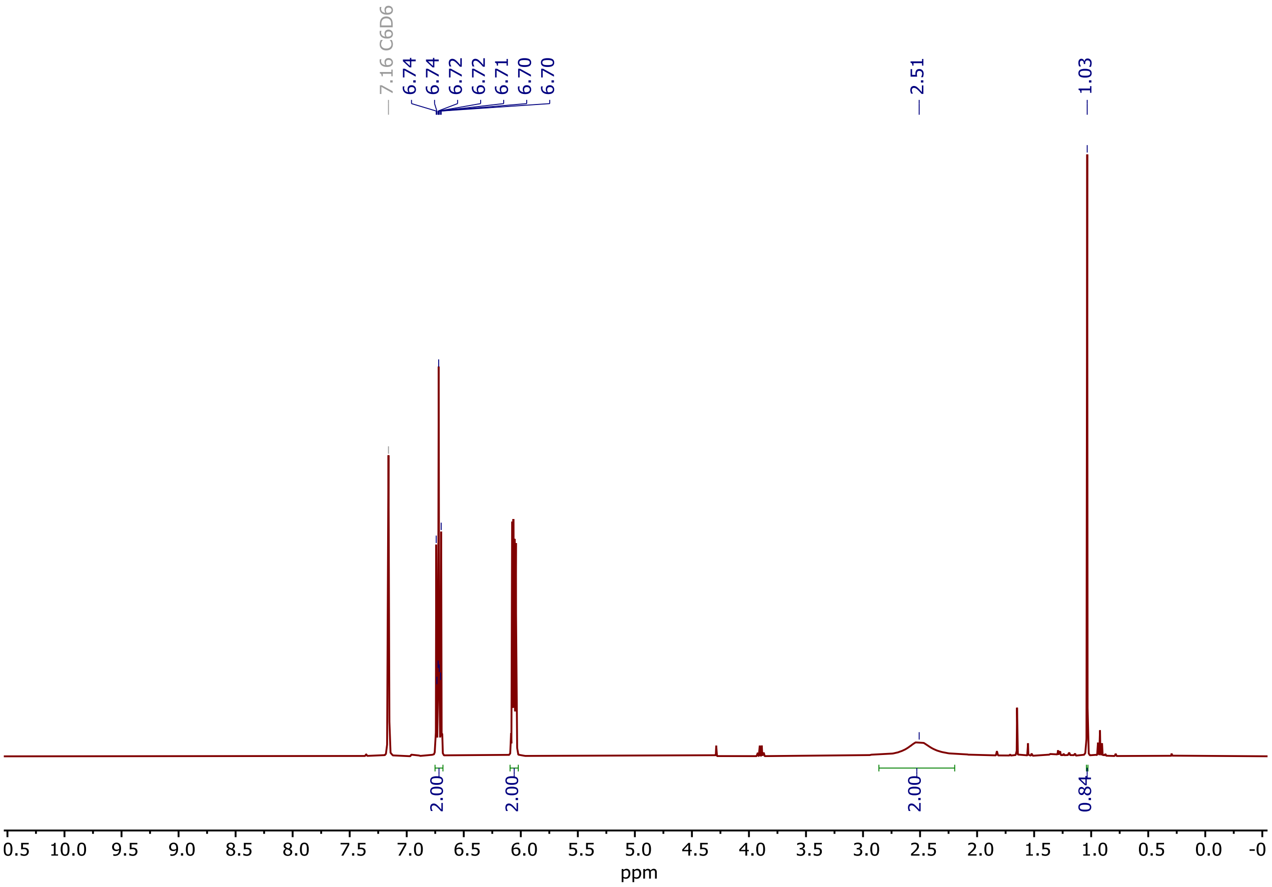

Figure S 32: ^{1}H-NMR spectrum of 4-fluoroaniline in benzene-d_6 at 298 K prior purification *via* column chromatography, containing ~7% of unreacted pinacol.

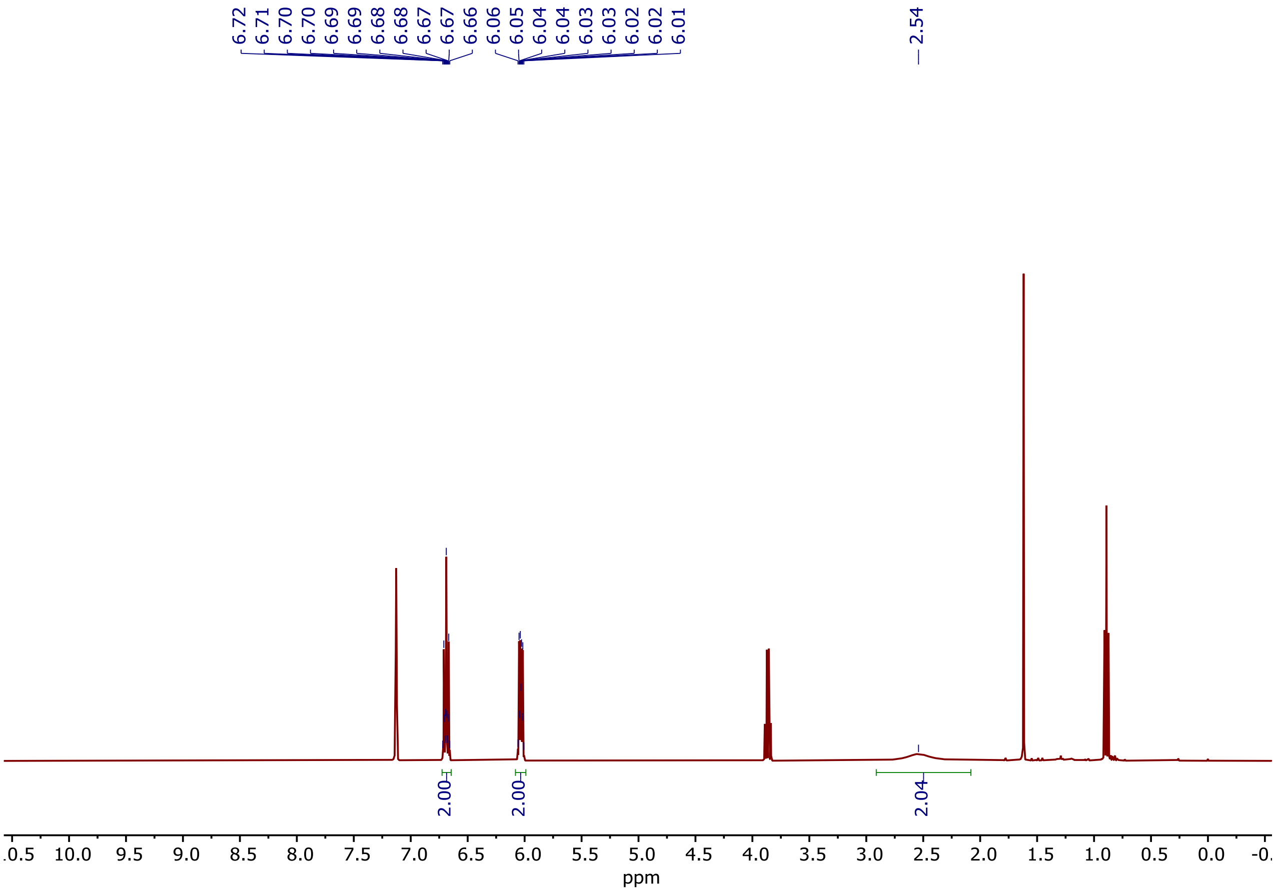

Figure S 33: ^{1}H-NMR spectrum of 4-fluoroaniline in benzene-d_6 at 298 K.

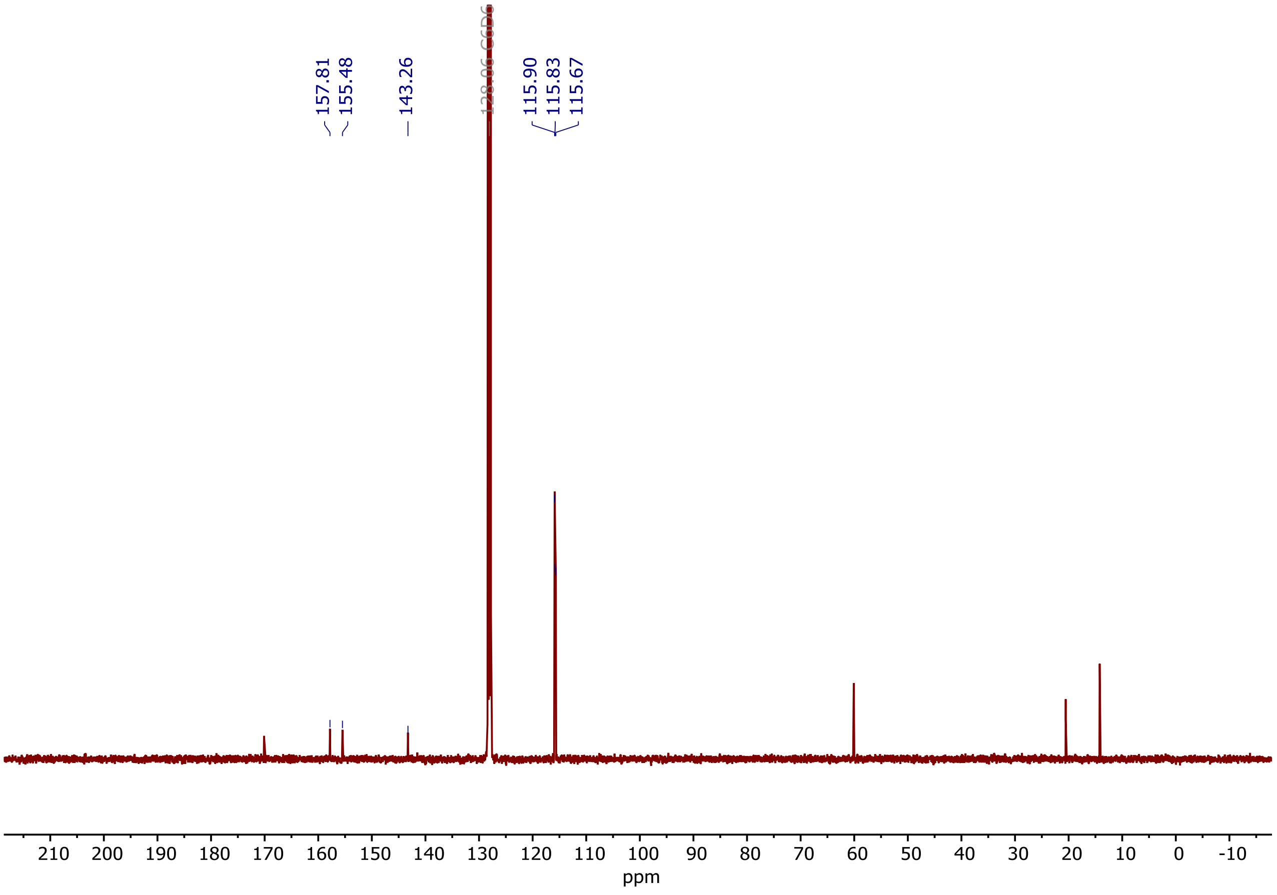

Figure S 34: $^{13}C\{^{1}H\}$-NMR spectrum of 4-fluoroaniline in benzene-d_6 at 298 K.

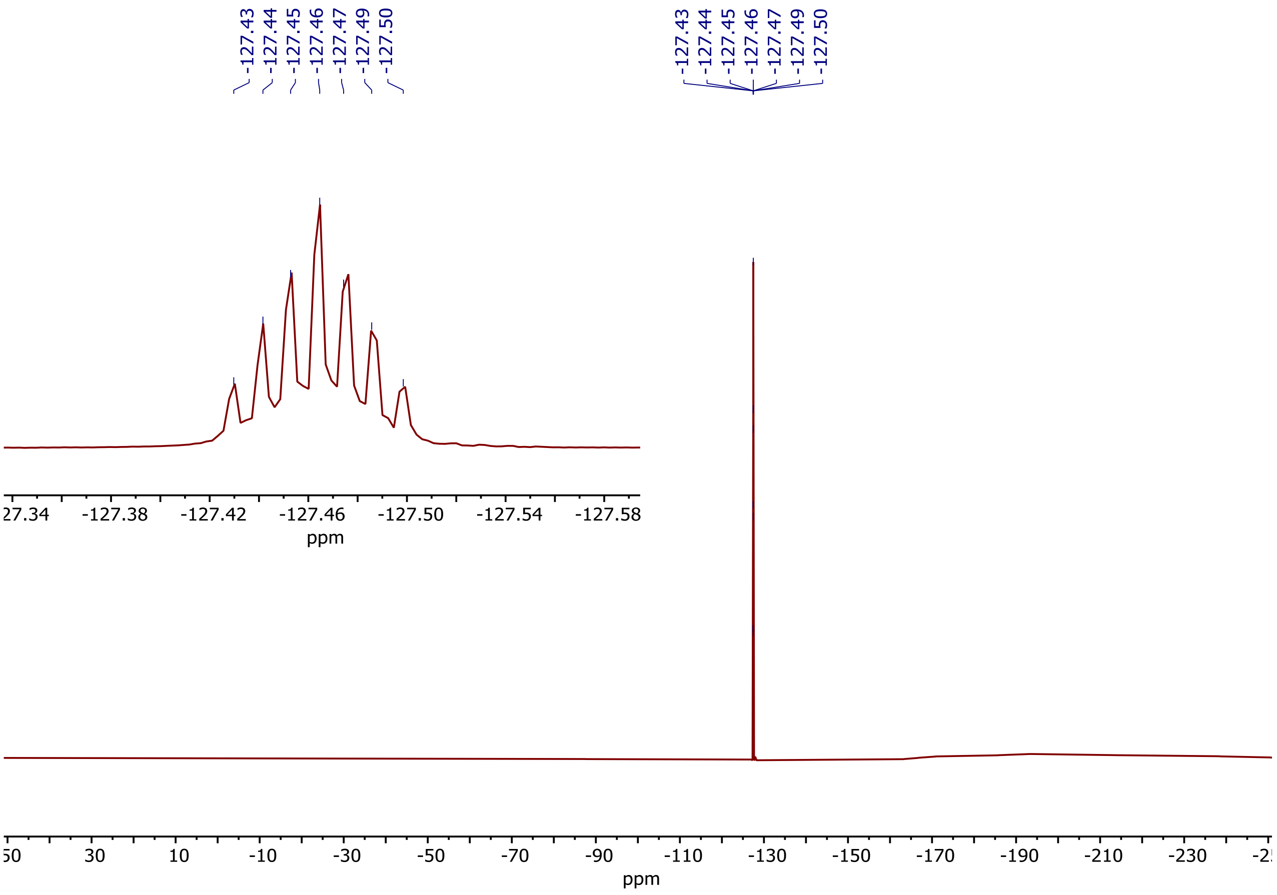

Figure S 35: ^{19}F-NMR spectrum of 4-fluoroanline in benzene-d_6 at 298 K.

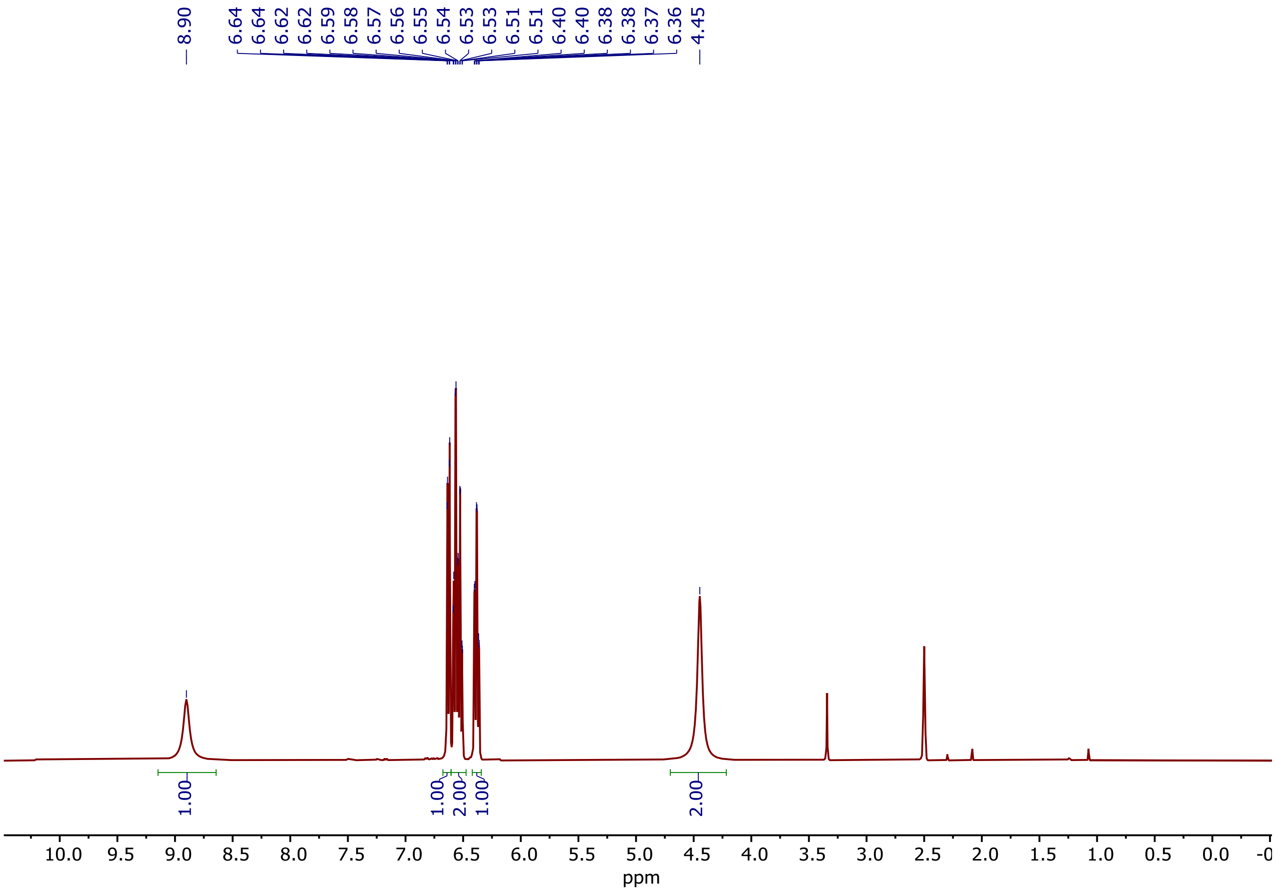

Figure S 36: ^{1}H-NMR spectrum of 2-aminophenol in dimethylsulfoxide-d_6 at 298 K.

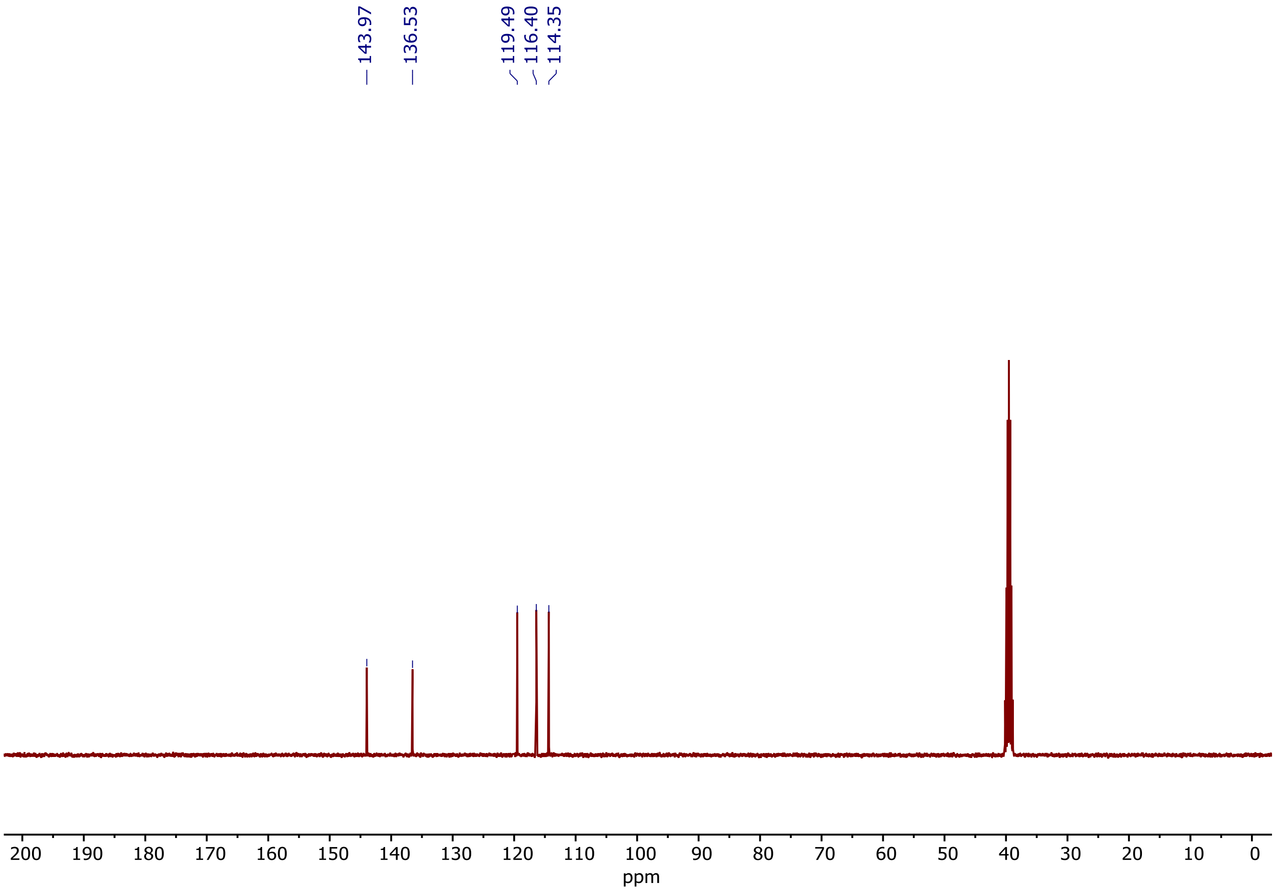

Figure S 37: $^{13}C\{^{1}H\}$-NMR spectrum of 2-aminophenol in dimethylsulfoxide-d_6 at 298 K.

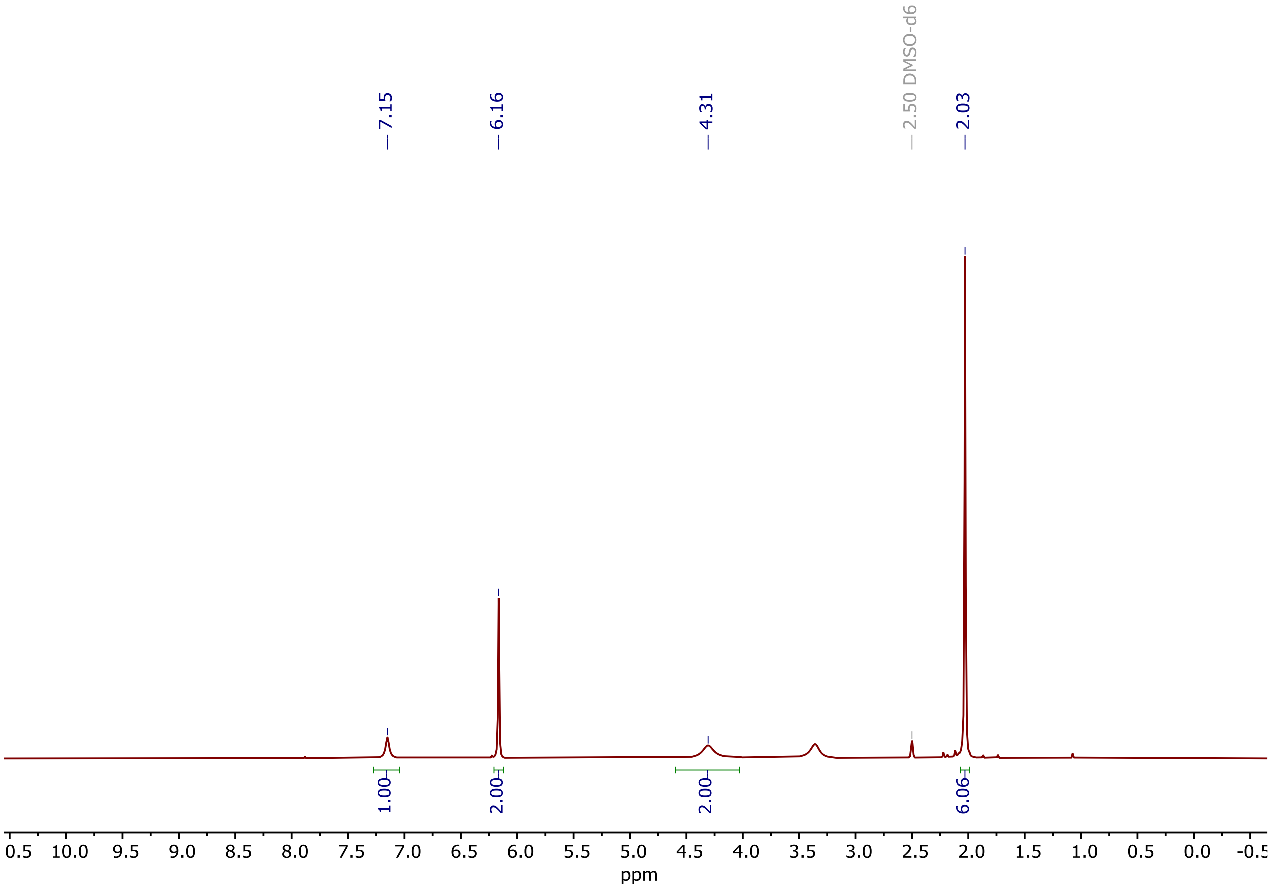

Figure S 38: ^{1}H-NMR spectrum of 4-amino-2,6-dimethylphenol in dimethylsulfoxide-d_6 at 298 K.

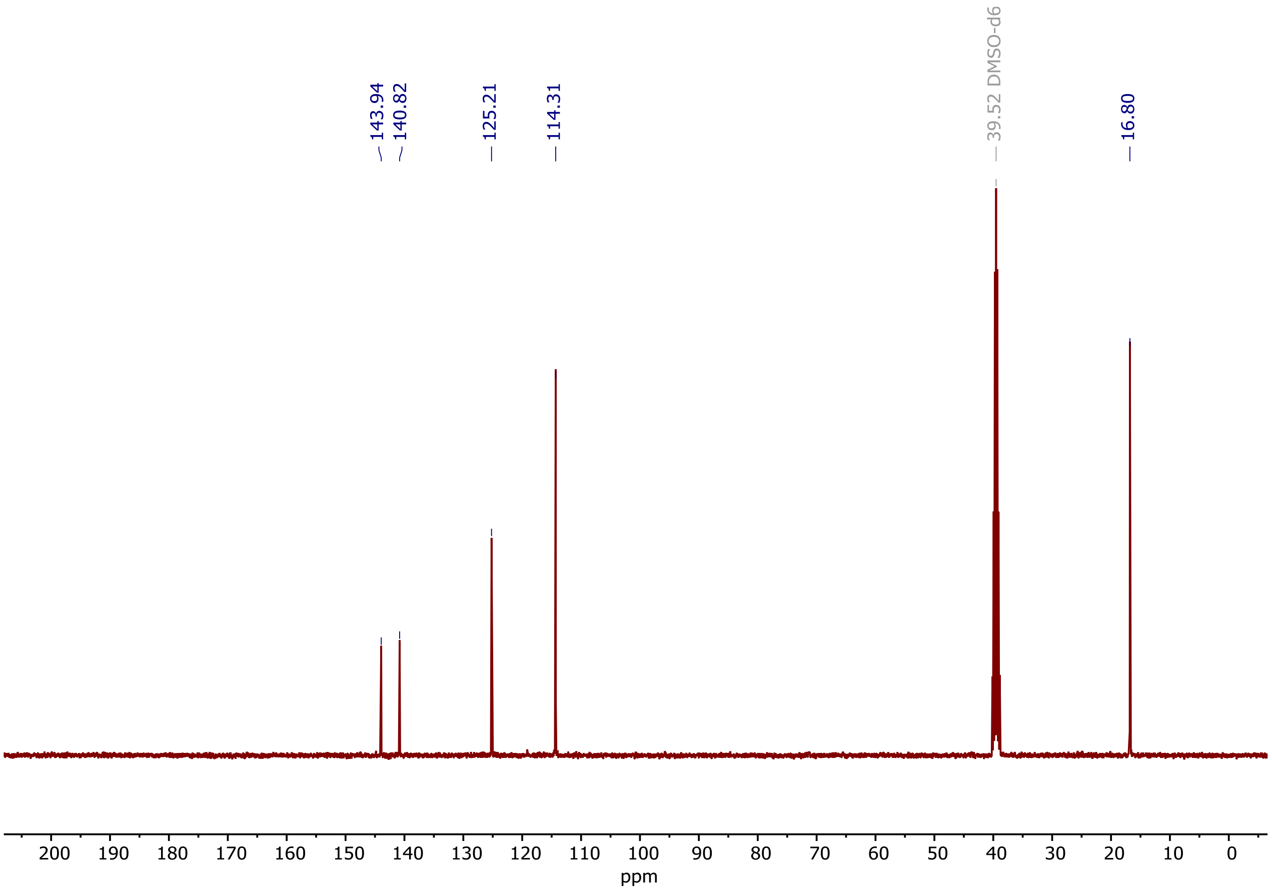

Figure S 39: ^{13}C{^{1}H}-NMR spectrum of 4-amino-2,6-dimethylphenol in dimethylsulfoxide-d_6 at 298 K.

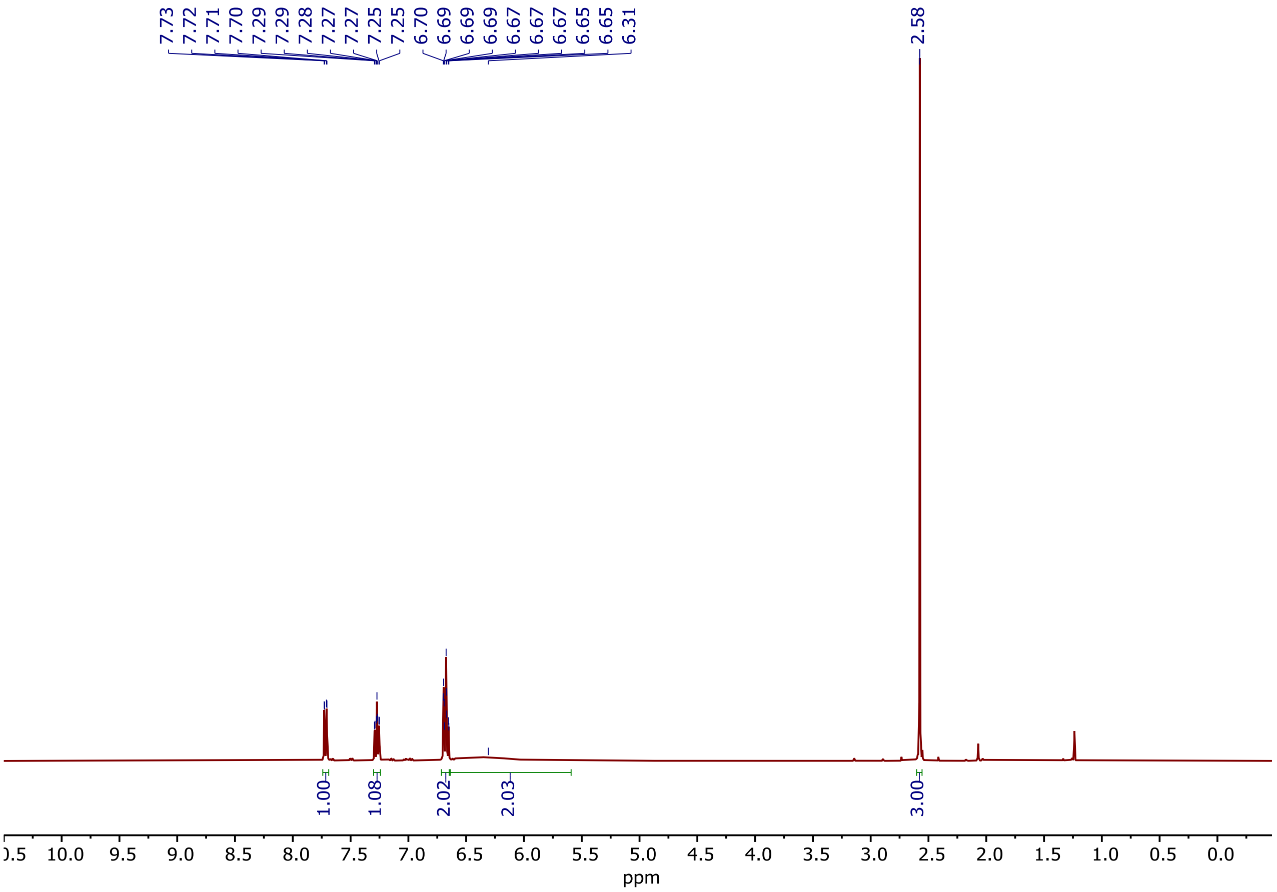

Figure S 40: ^{1}H-NMR spectrum of 2'-aminoacetophenone in chloroform-*d* at 298 K.

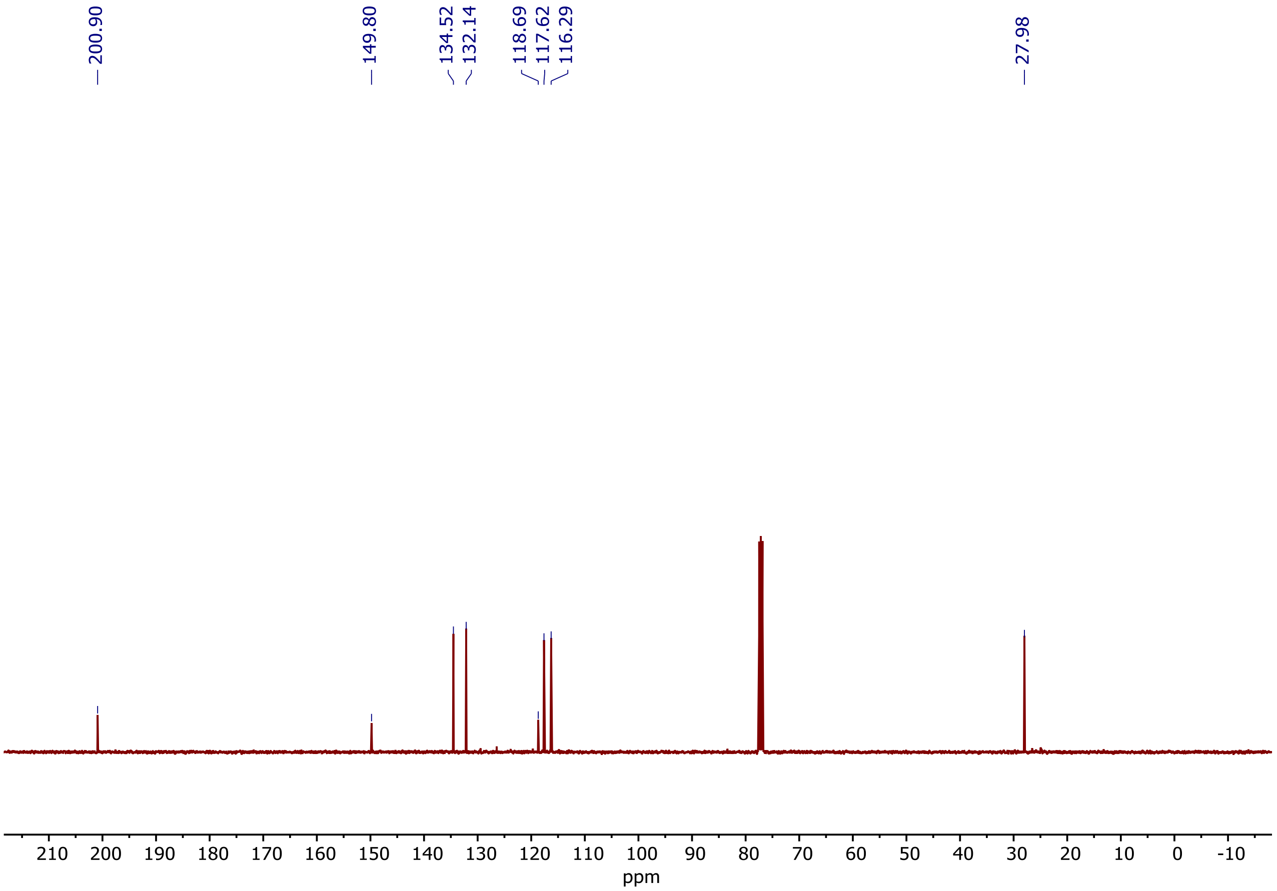

Figure S 41: ^{13}C{^{1}H}-NMR spectrum of 2'-aminoacetophenone in chloroform-*d* at 298 K.

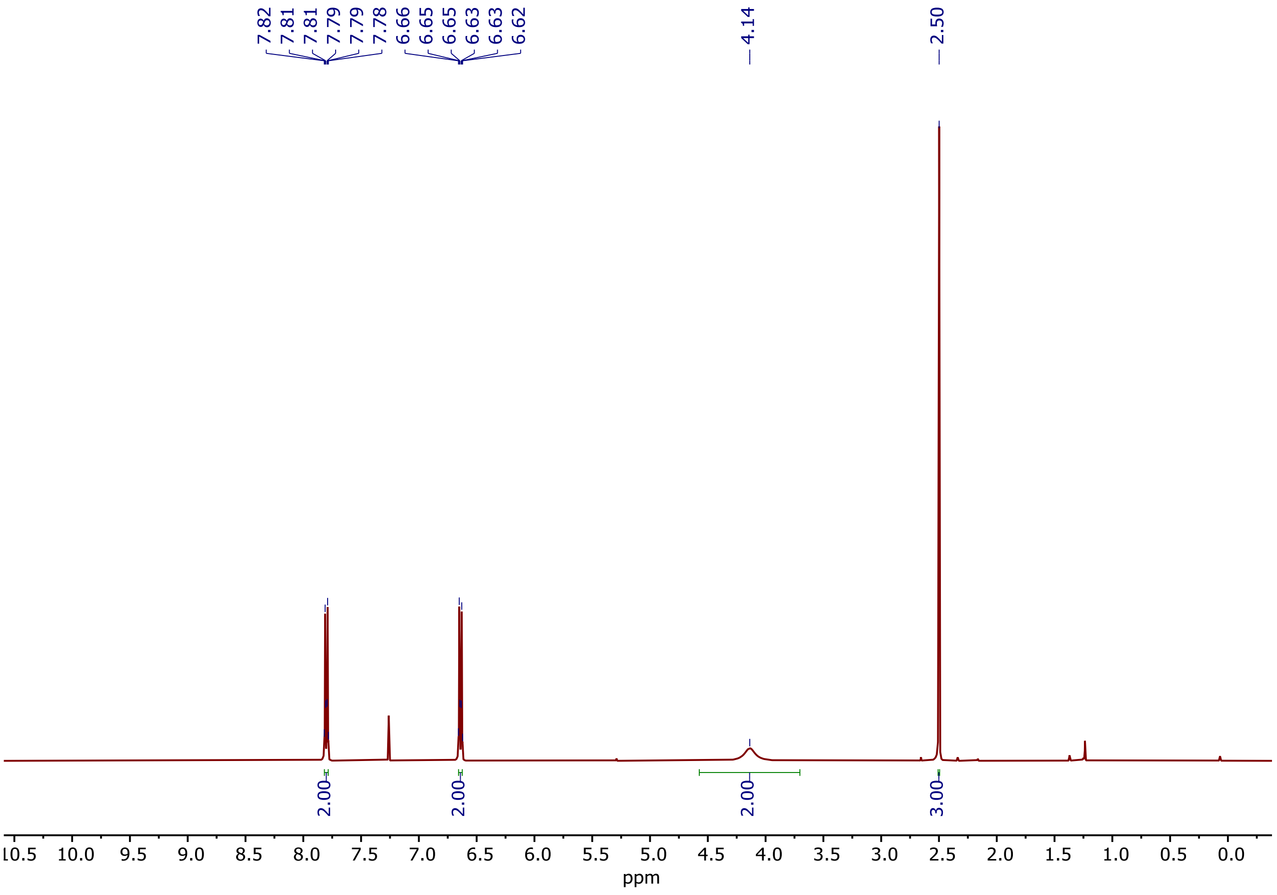

Figure S 42: ^{1}H-NMR spectrum of 4'-aminoacetophenone in chloroform-*d* at 298 K.

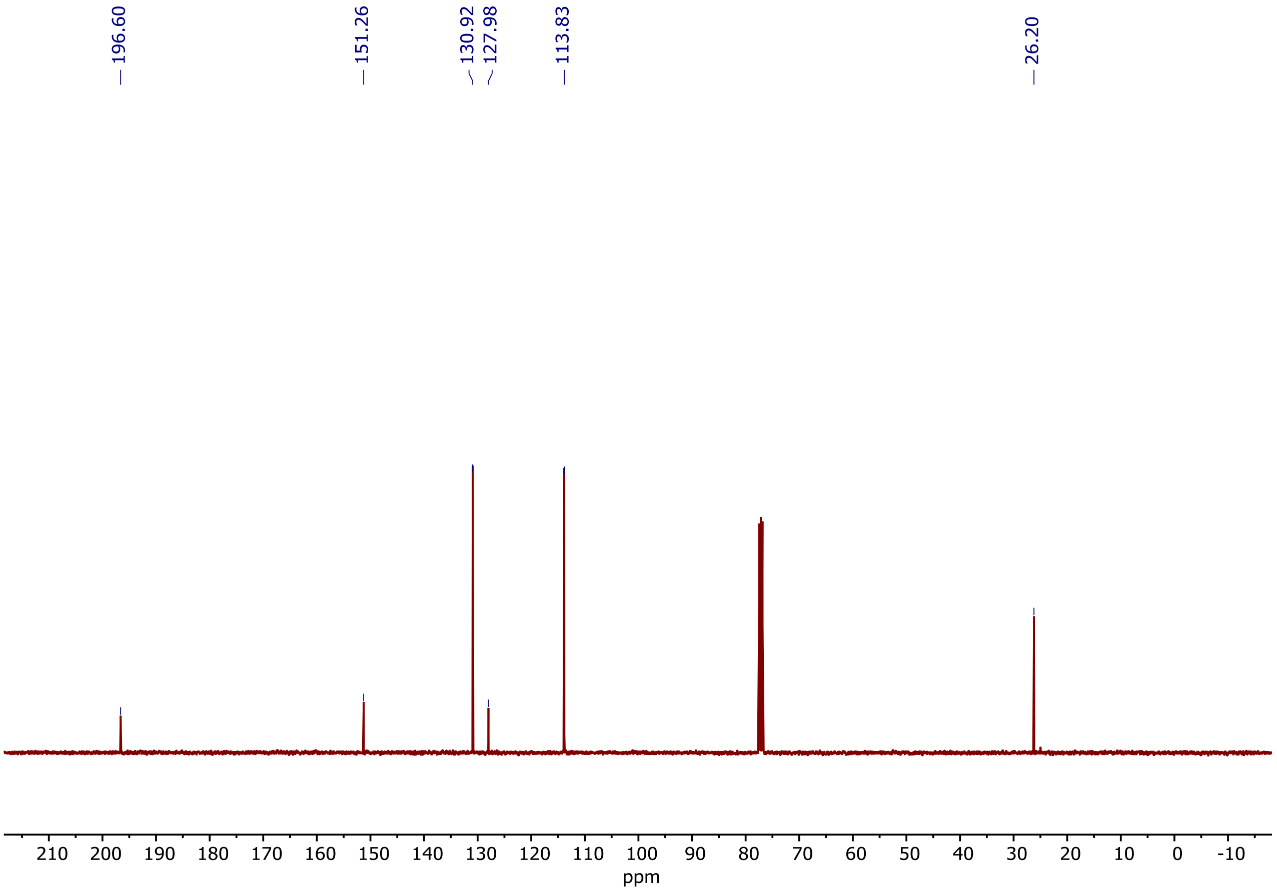

Figure S 43: ^{13}C{^{1}H}-NMR spectrum of 4'-aminoacetophenone in chloroform-*d* at 298 K.

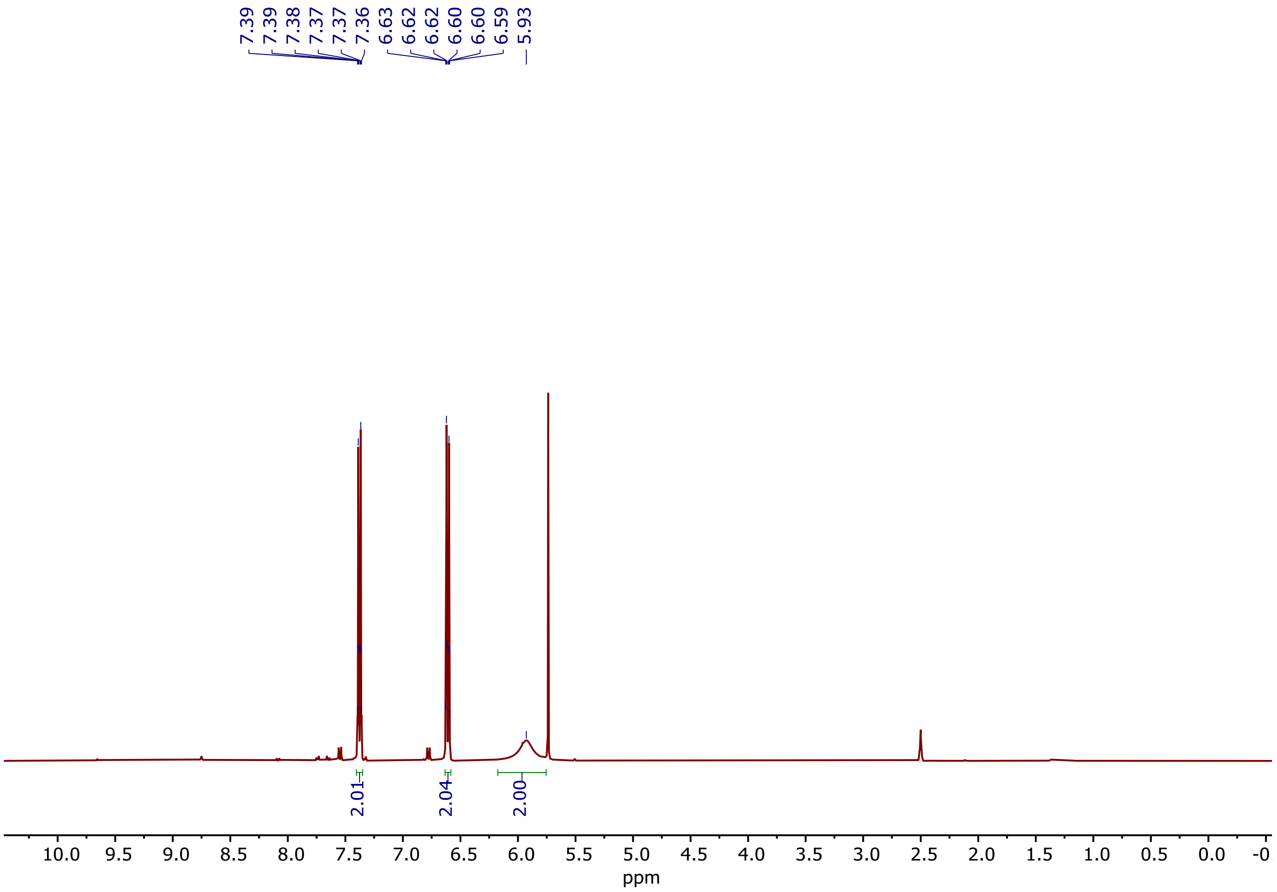

Figure S 44: ^{1}H-NMR spectrum of 4-aminobenzonitrile in dimethylsulfoxide-d_6 at 298 K.

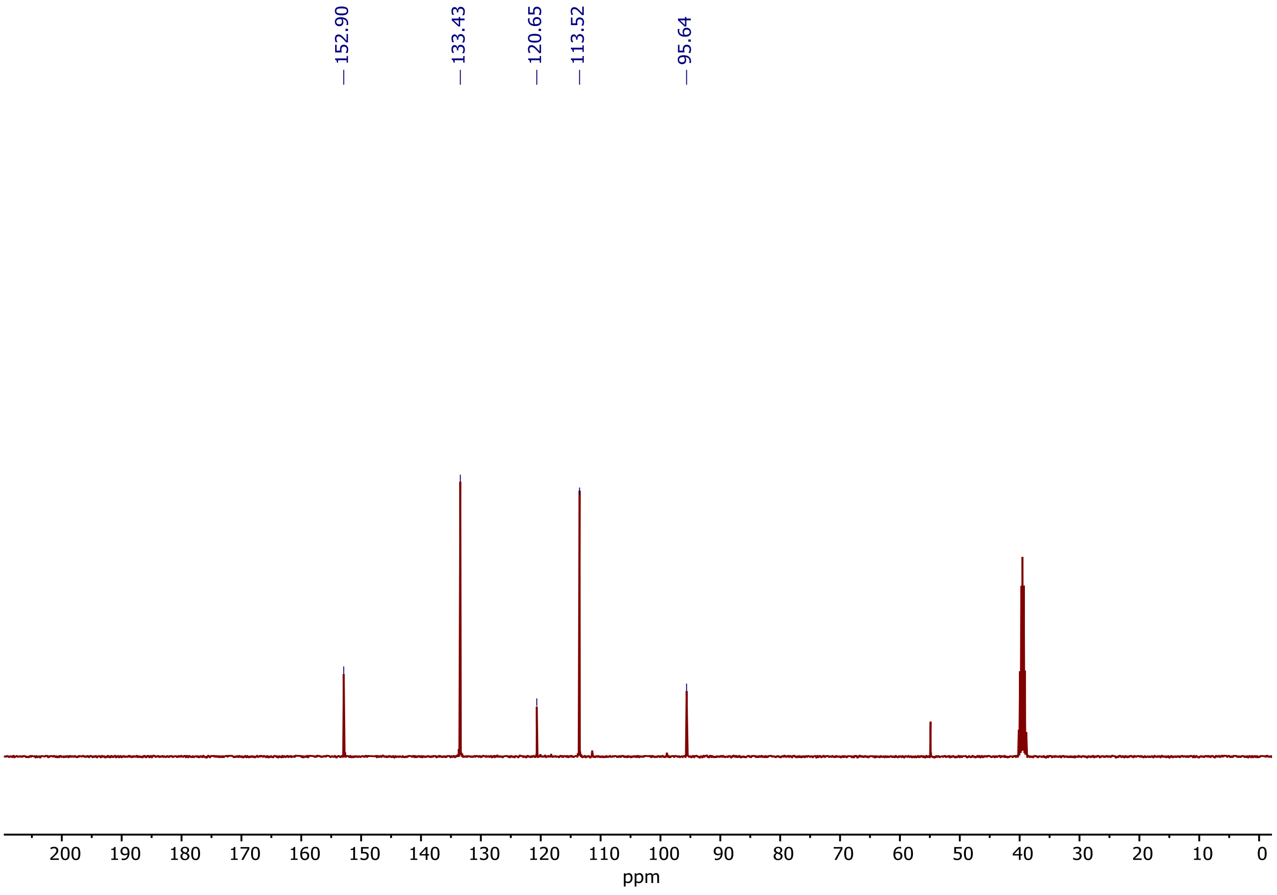

Figure S 45: ^{13}C{^{1}H}-NMR spectrum of 4-aminobenzonitrile in dimethylsulfoxide-d_6 at 298 K.

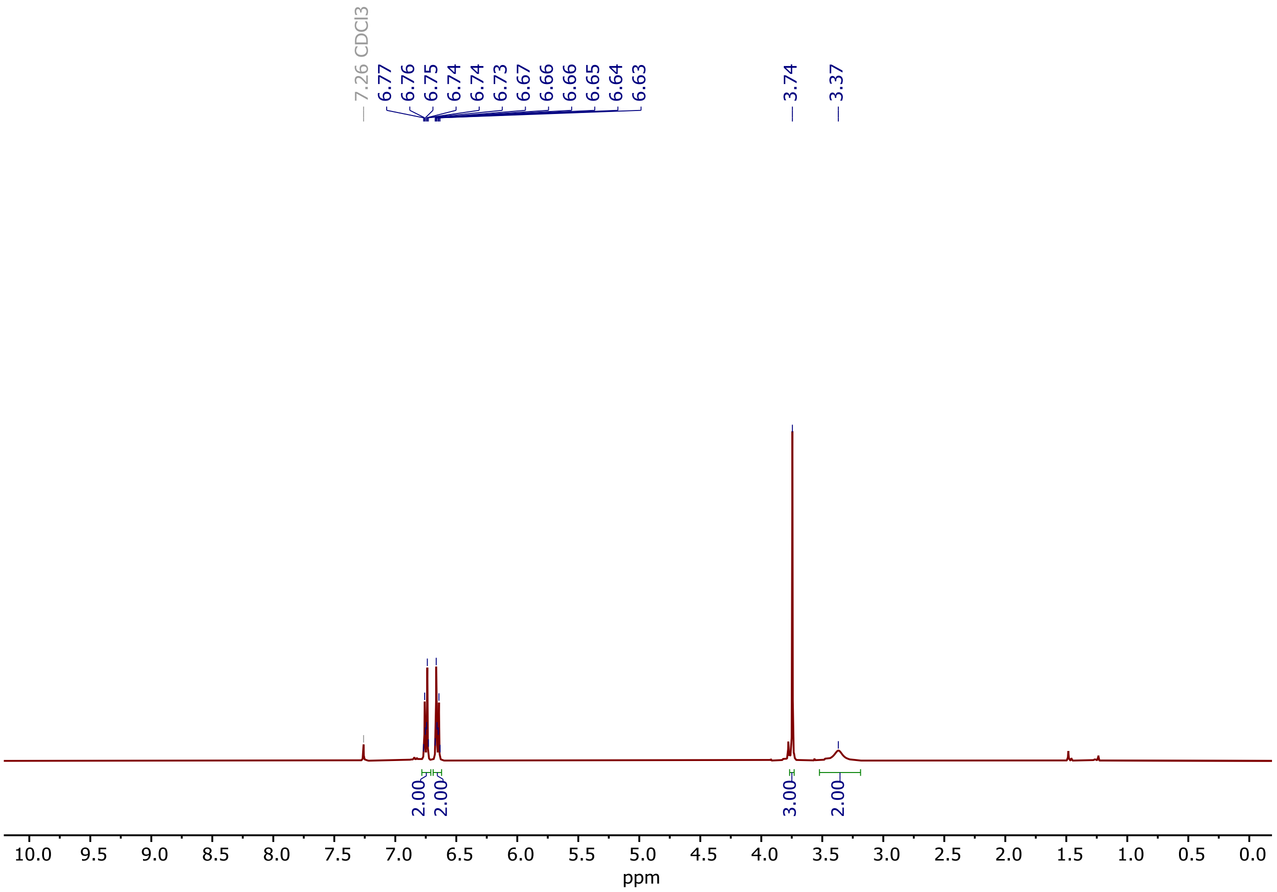

Figure S 46: ^{1}H-NMR spectrum of 4-aminoanisole in chloroform-*d* at 298 K.

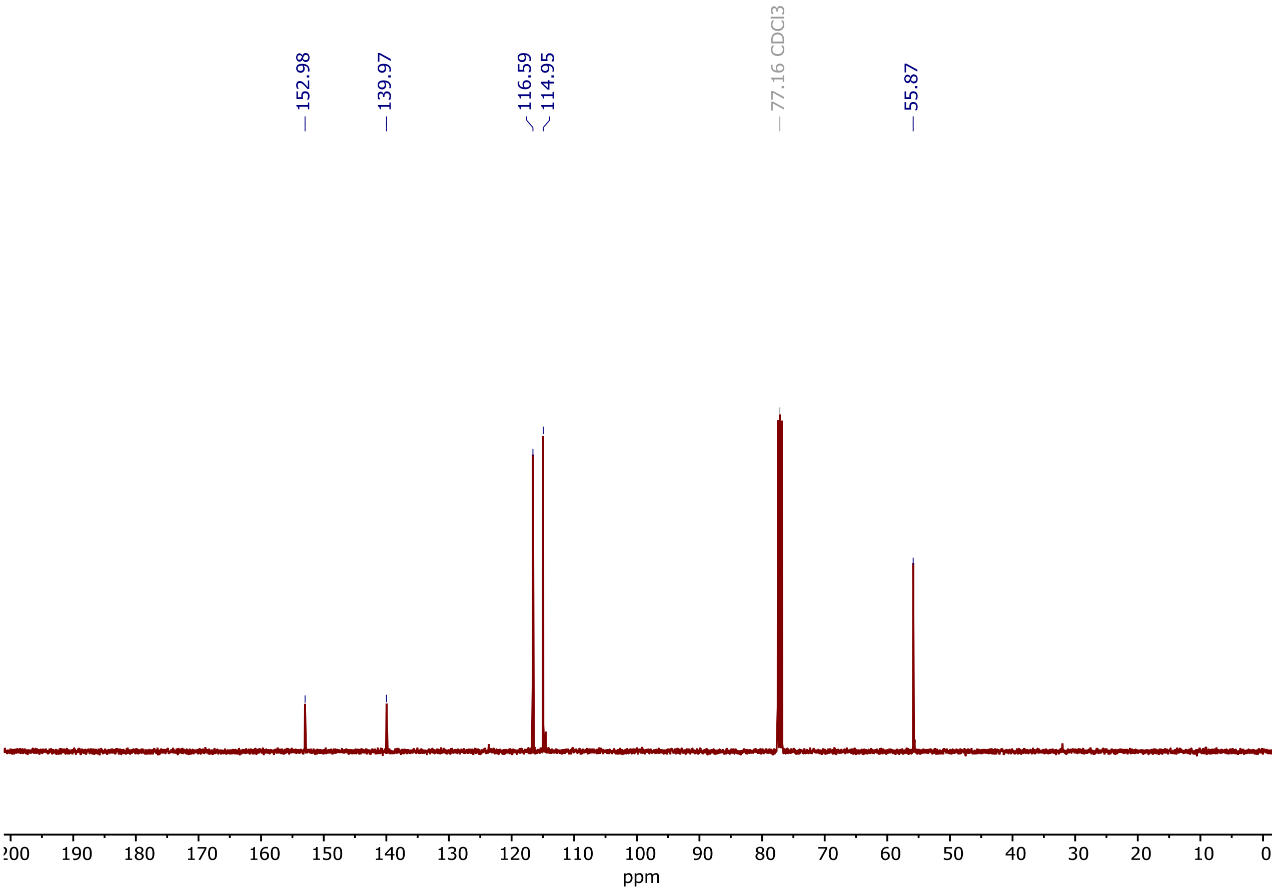

Figure S 47: ^{13}C{^{1}H}-NMR spectrum of 4-aminoanisole in chloroform-*d* at 298 K.

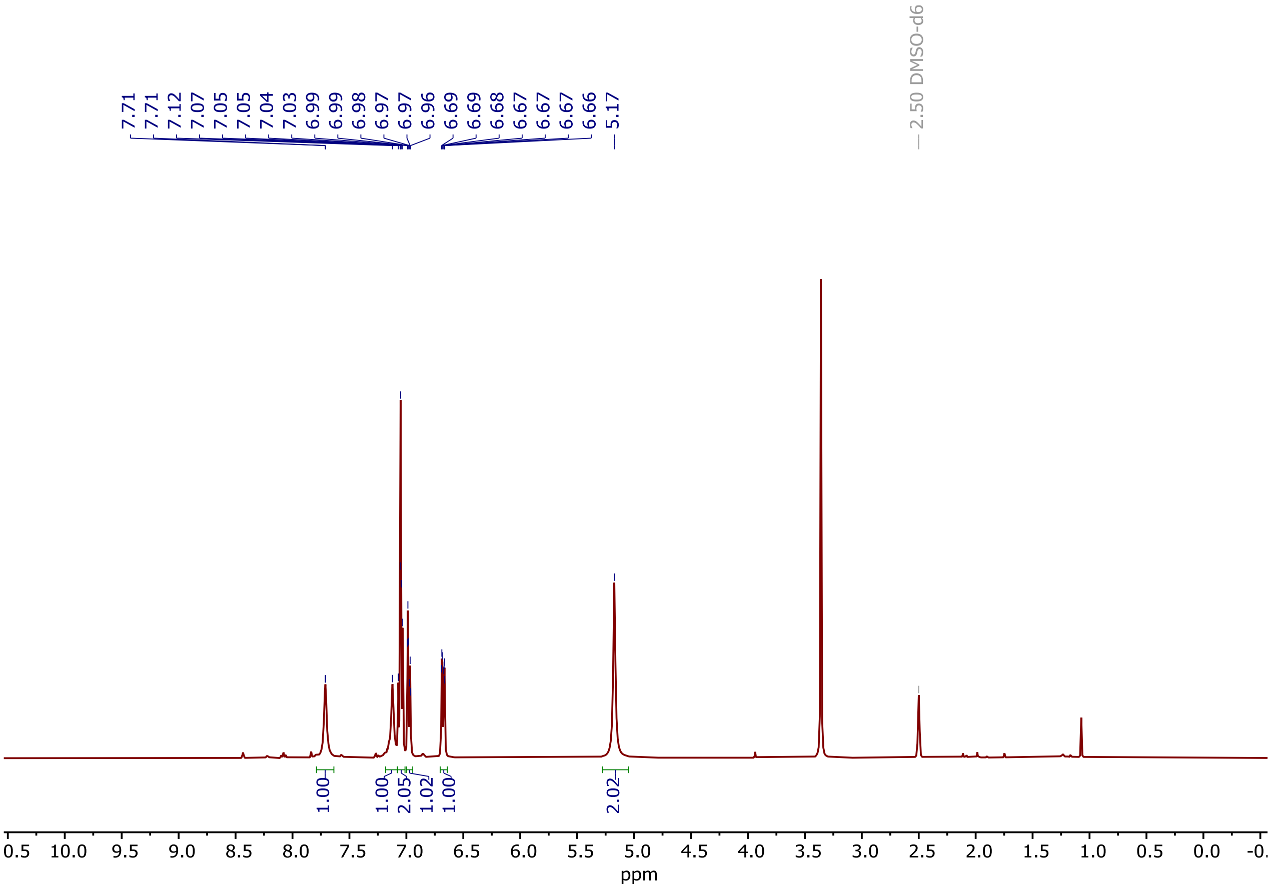

Figure S 48: ^{1}H-NMR spectrum of 3-aminobenzamide in dimethylsulfoxide-d_6 at 298 K.

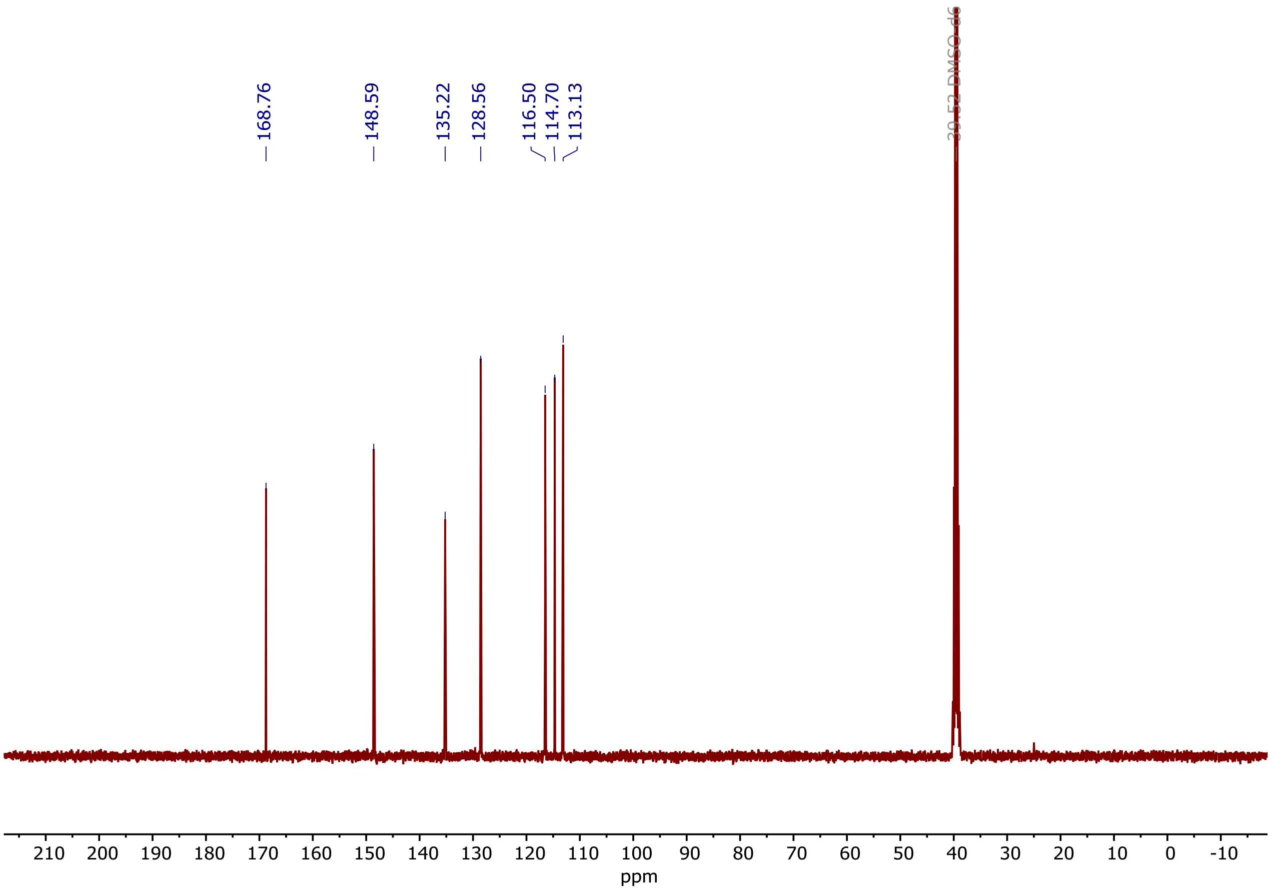

Figure S 49: ^{13}C{^{1}H}-NMR spectrum of 3-aminobenzamide in dimethylsulfoxide-d_6 at 298 K.

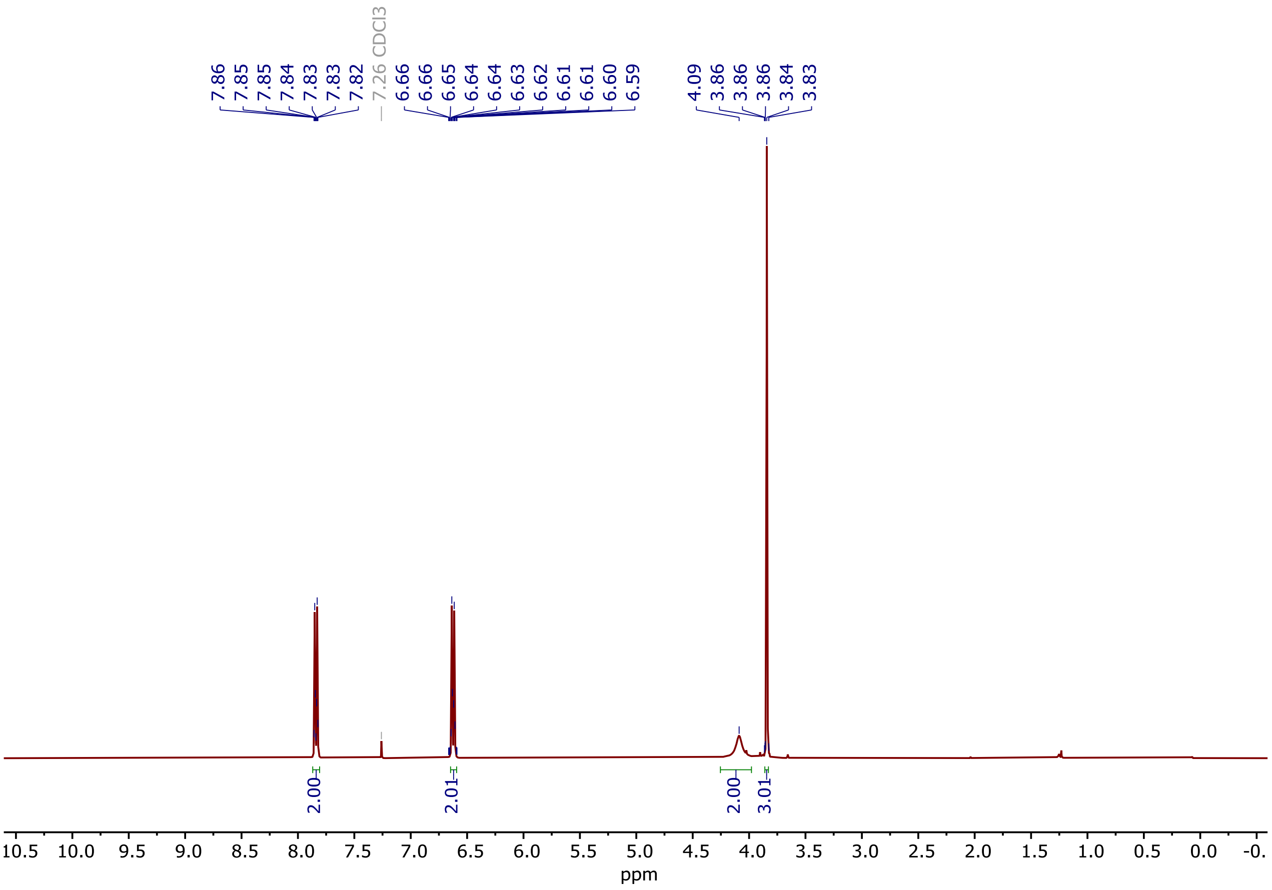

Figure S 50: ^{1}H-NMR spectrum of methyl 4-aminobenzoate in chloroform-*d* at 298 K.

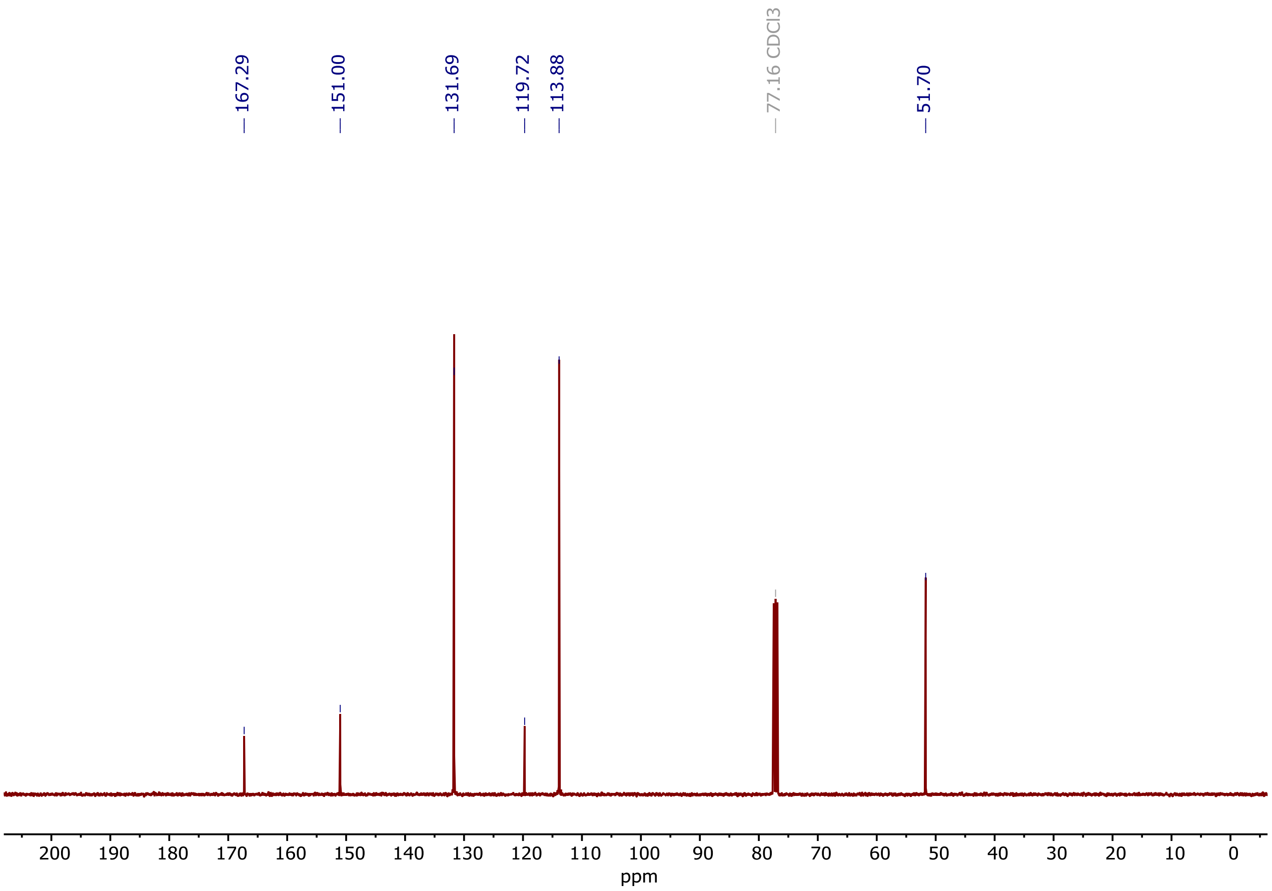

Figure S 51: ^{1}H{^{13}C}-NMR spectrum of methyl 4-aminobenzoate in chloroform-*d* at 298 K.

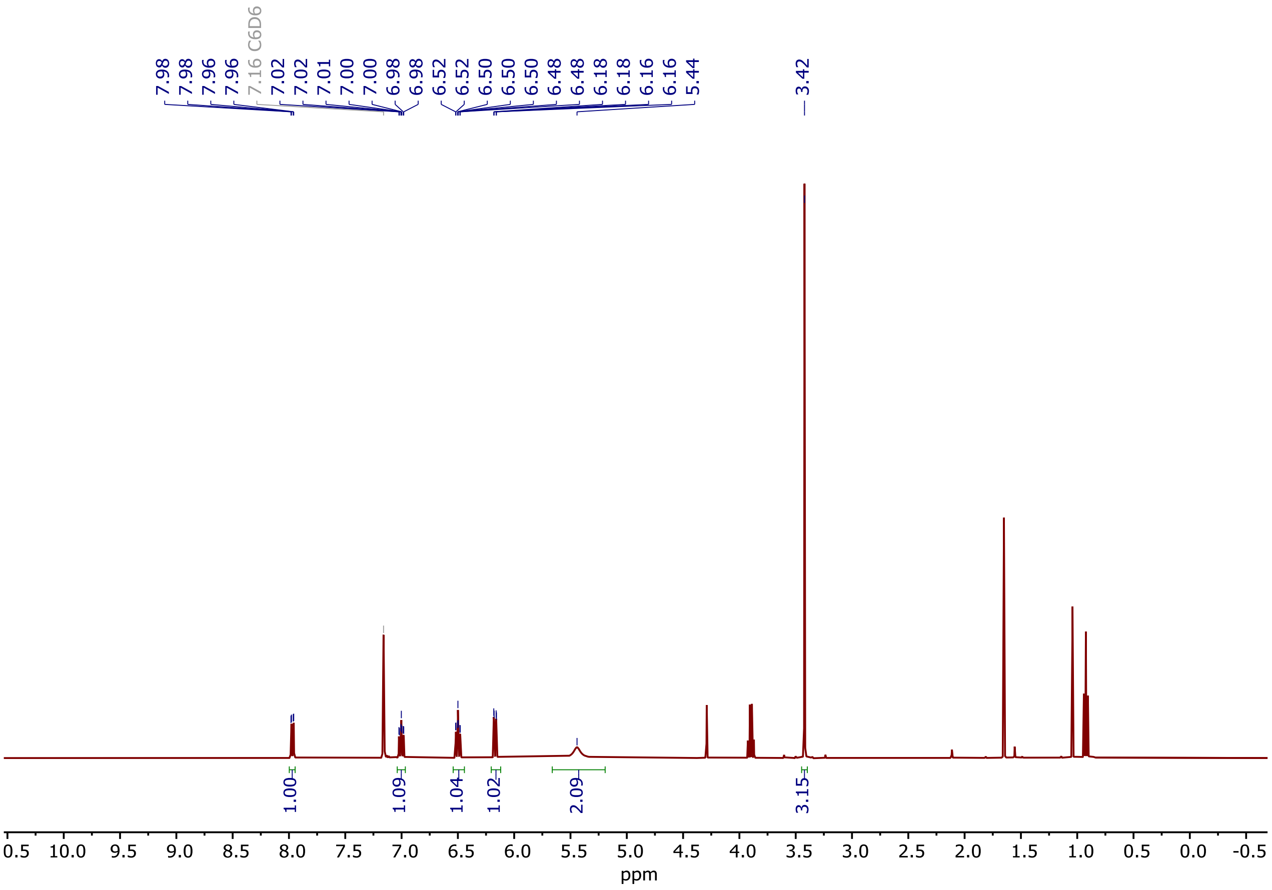

Figure S 52: ^{1}H-NMR spectrum of methyl 2-aminobenzoate in benzene-d_6 at 298 K.

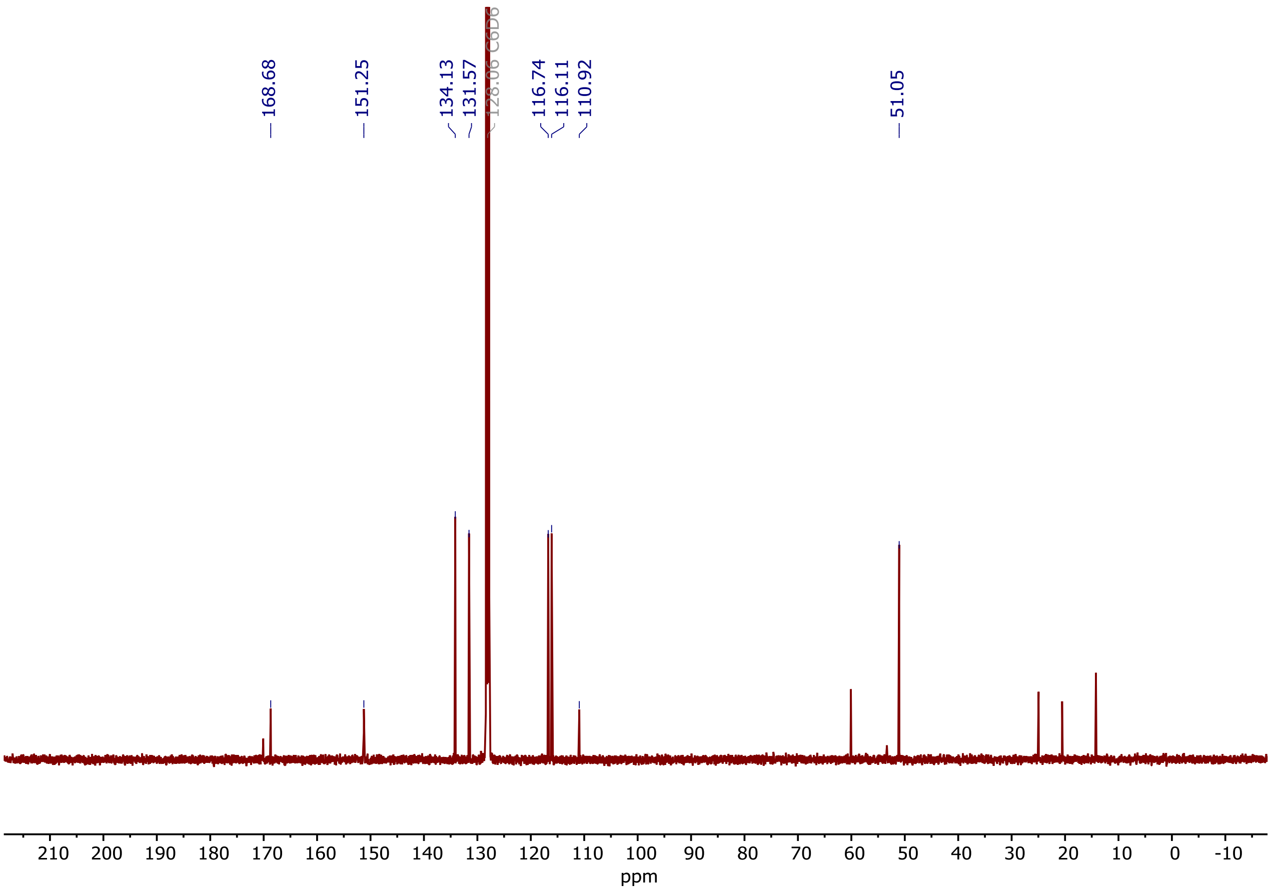

Figure S 53: ^{13}C{^{1}H}-NMR spectrum of methyl 2-aminobenzoate in benzene-d_6 at 298 K.

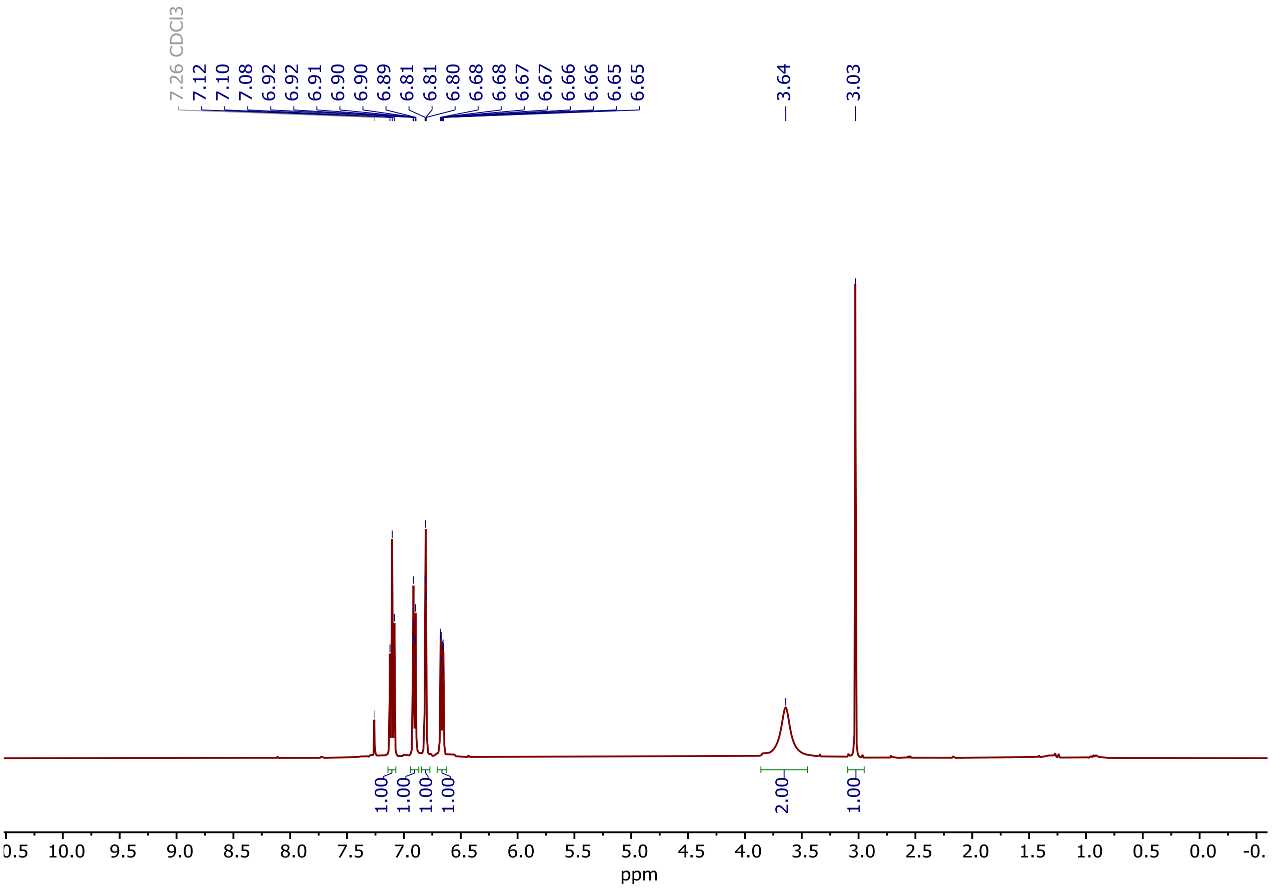

Figure S 54: ^{1}H-NMR spectrum of 3-ethynylaniline in chloroform-*d* at 298 K.

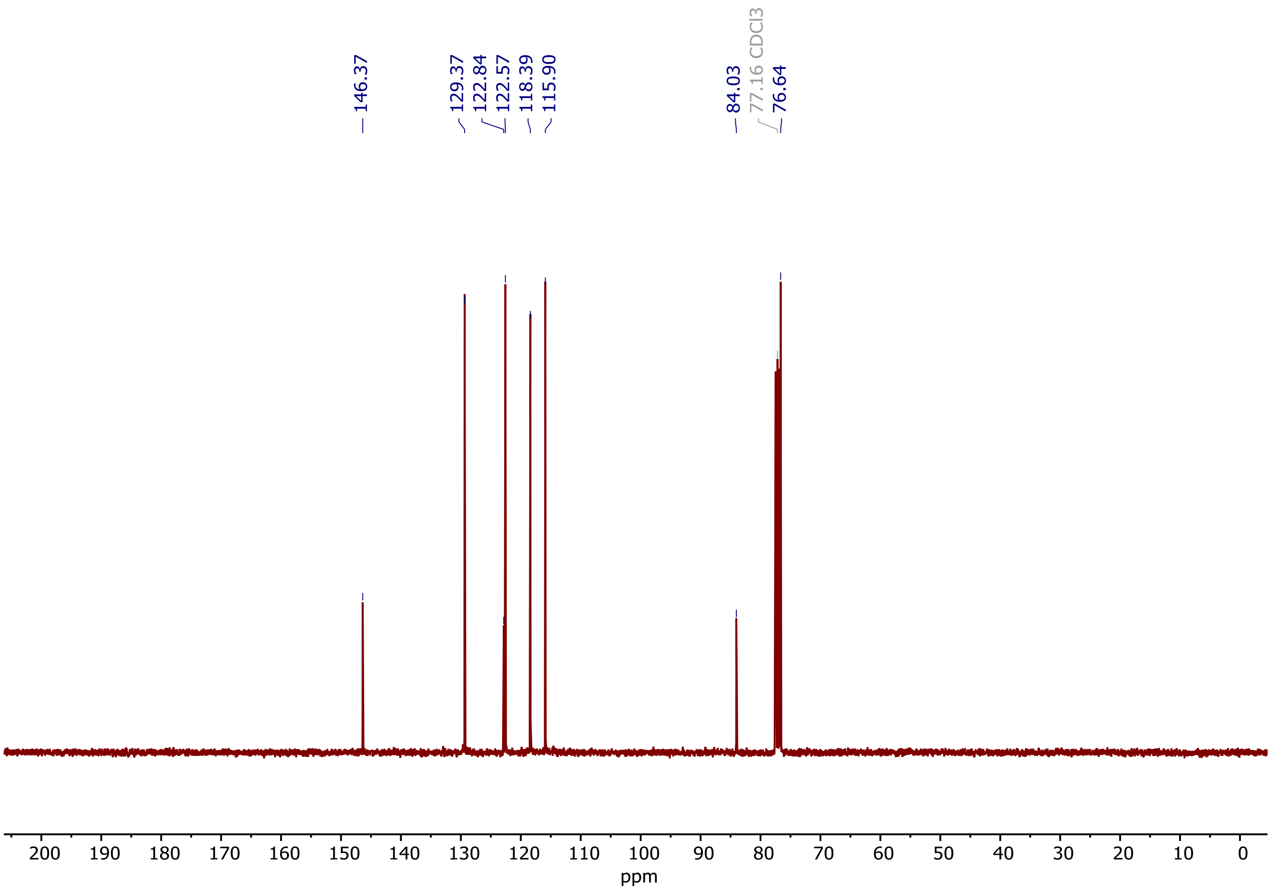

Figure S 55: $^{13}C\{^{1}H\}$-NMR spectrum of 3-ethynylaniline in chloroform-*d* at 298 K.

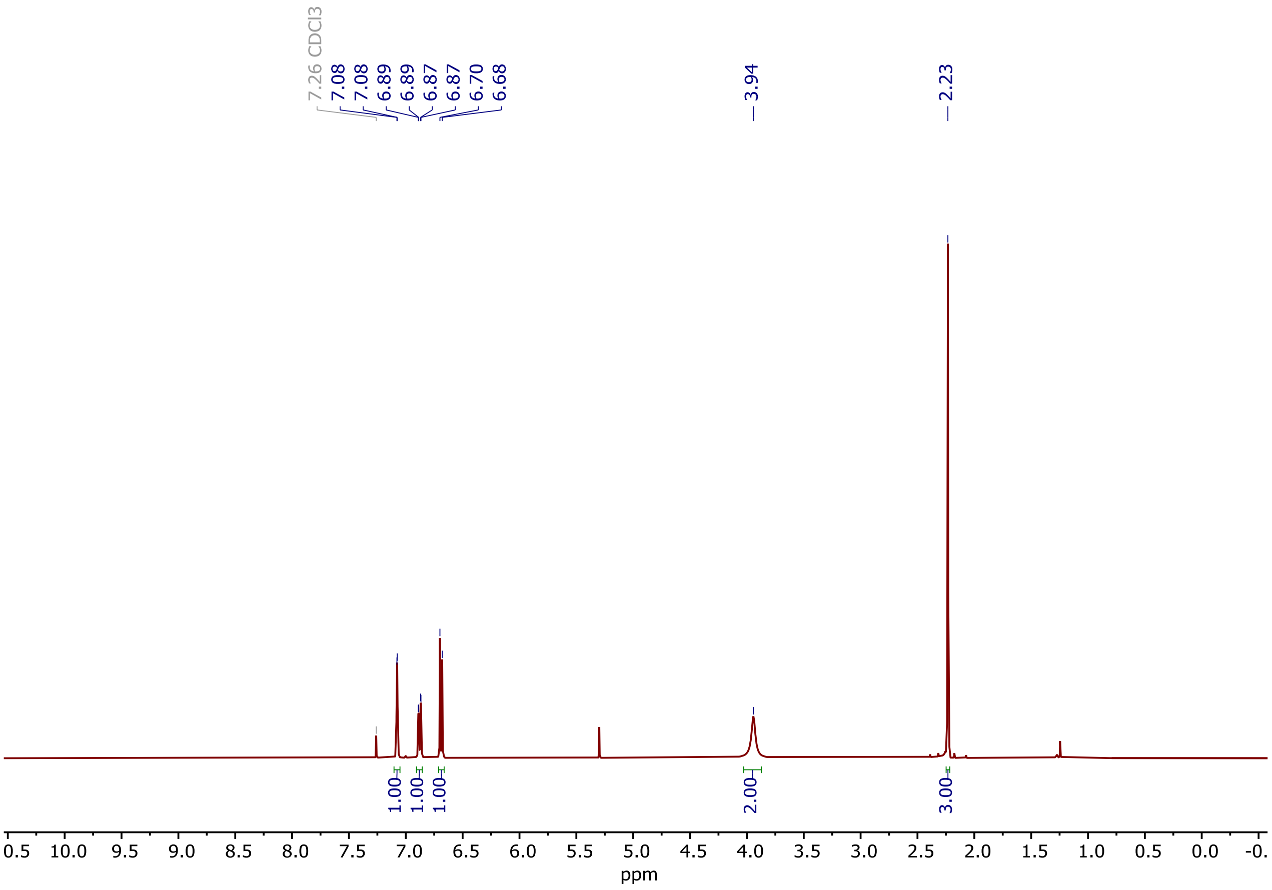

Figure S 56: ^{1}H-NMR spectrum of 2-chloro-p-toluidine in chloroform-*d* at 298 K.

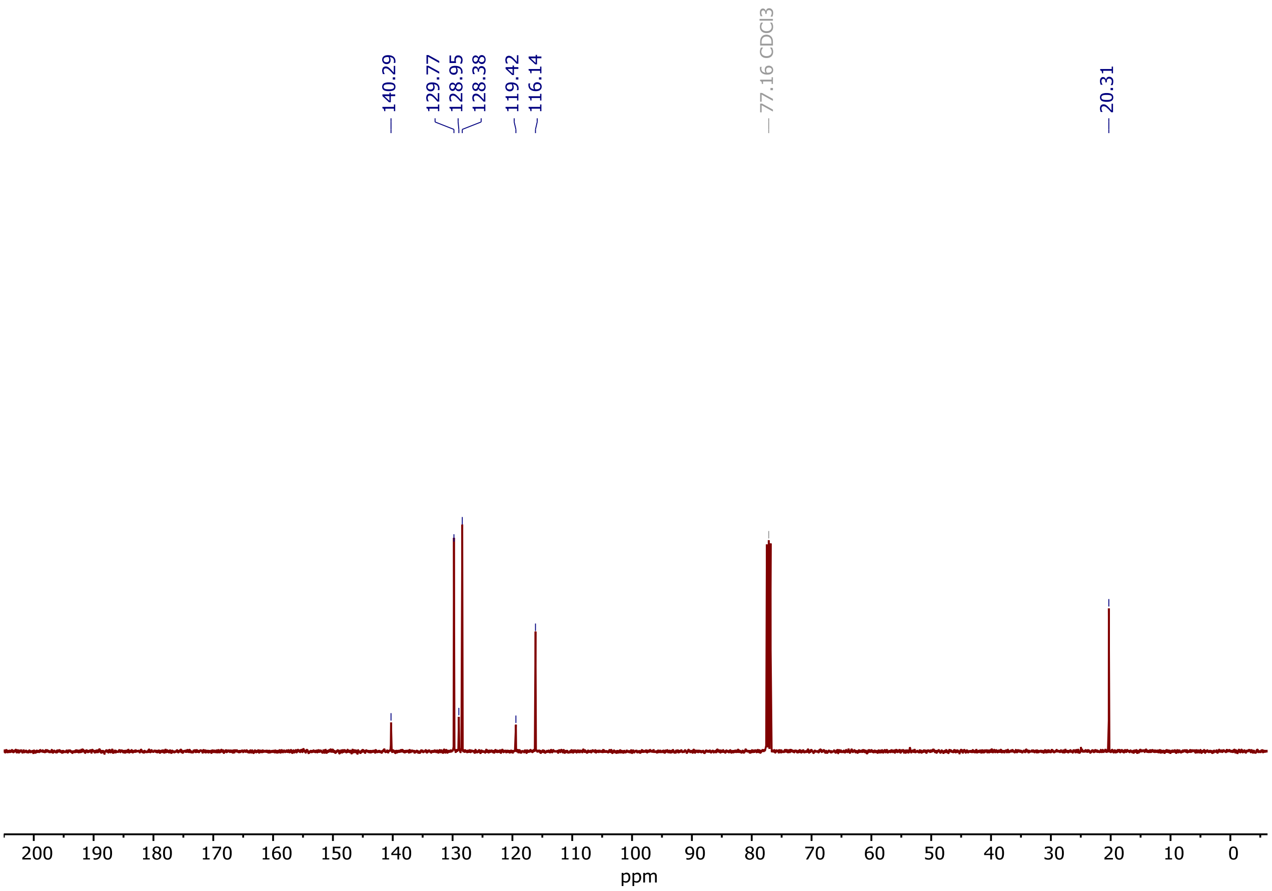

Figure S 57: $^{13}C\{^{1}H\}$-NMR spectrum of 2-chloro-p-toluidine in chloroform-*d* at 298 K.

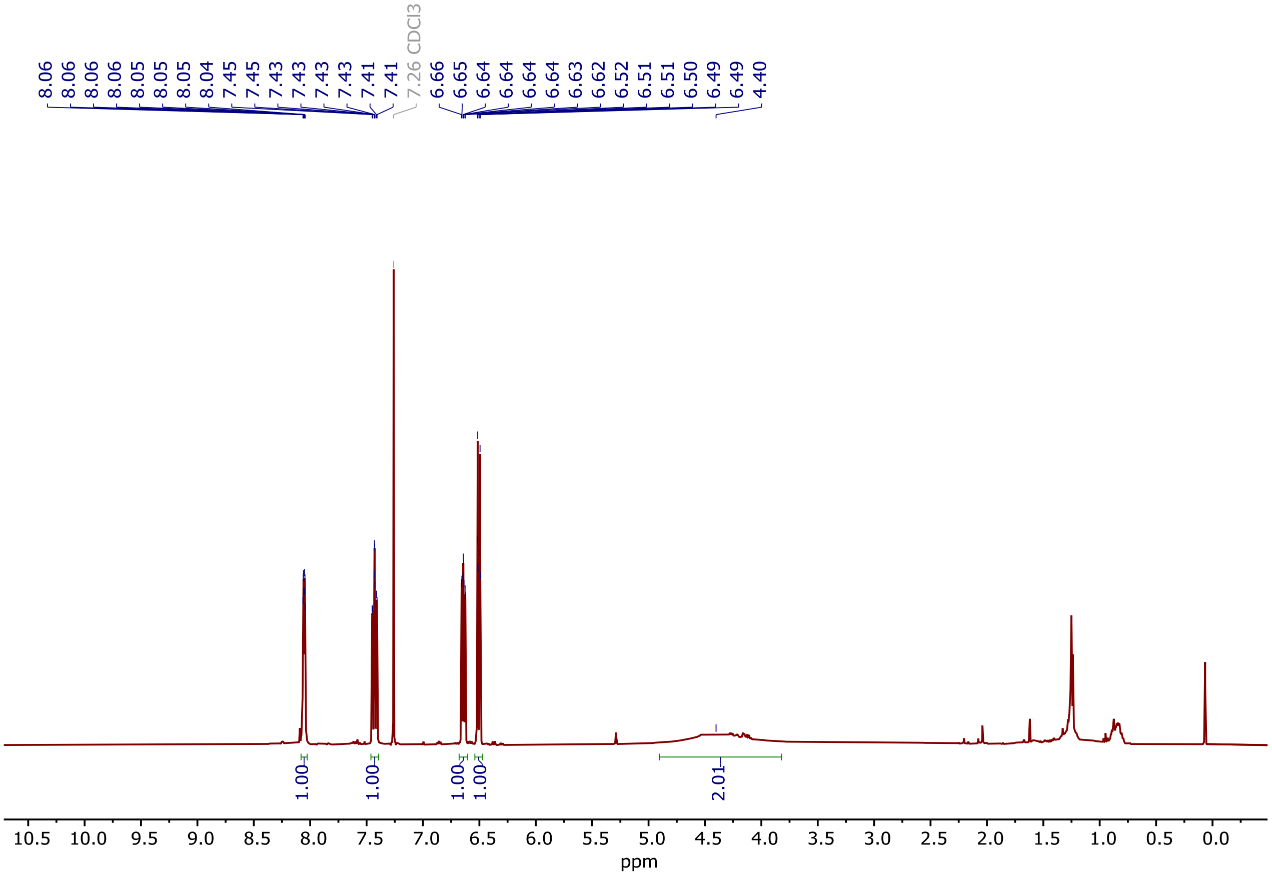

Figure S 58: ^{1}H-NMR spectrum of 2-aminopyridine in chloroform-*d* at 298 K.

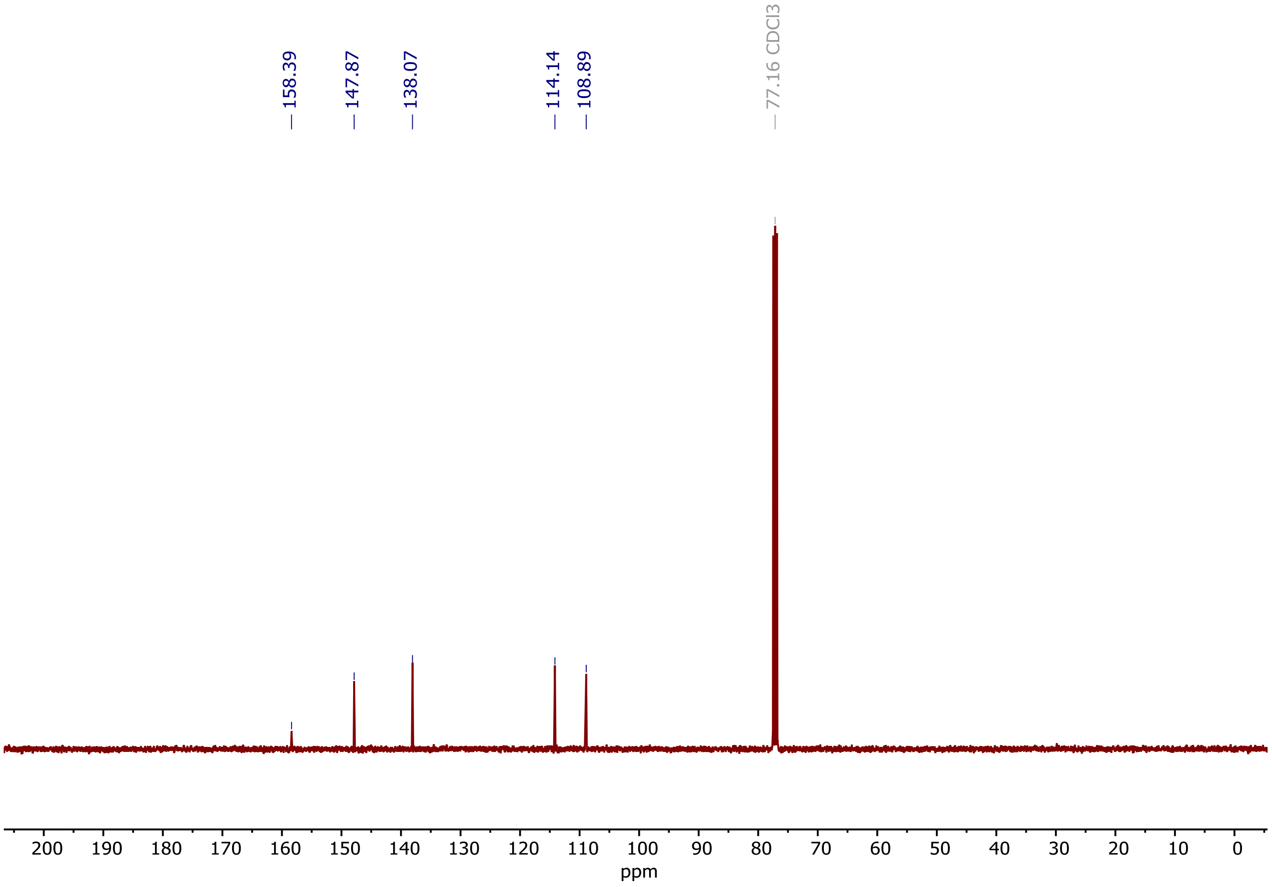

Figure S 59: ^{13}C{^{1}H}-NMR spectrum of 2-aminopyridine in chloroform-*d* at 298 K.

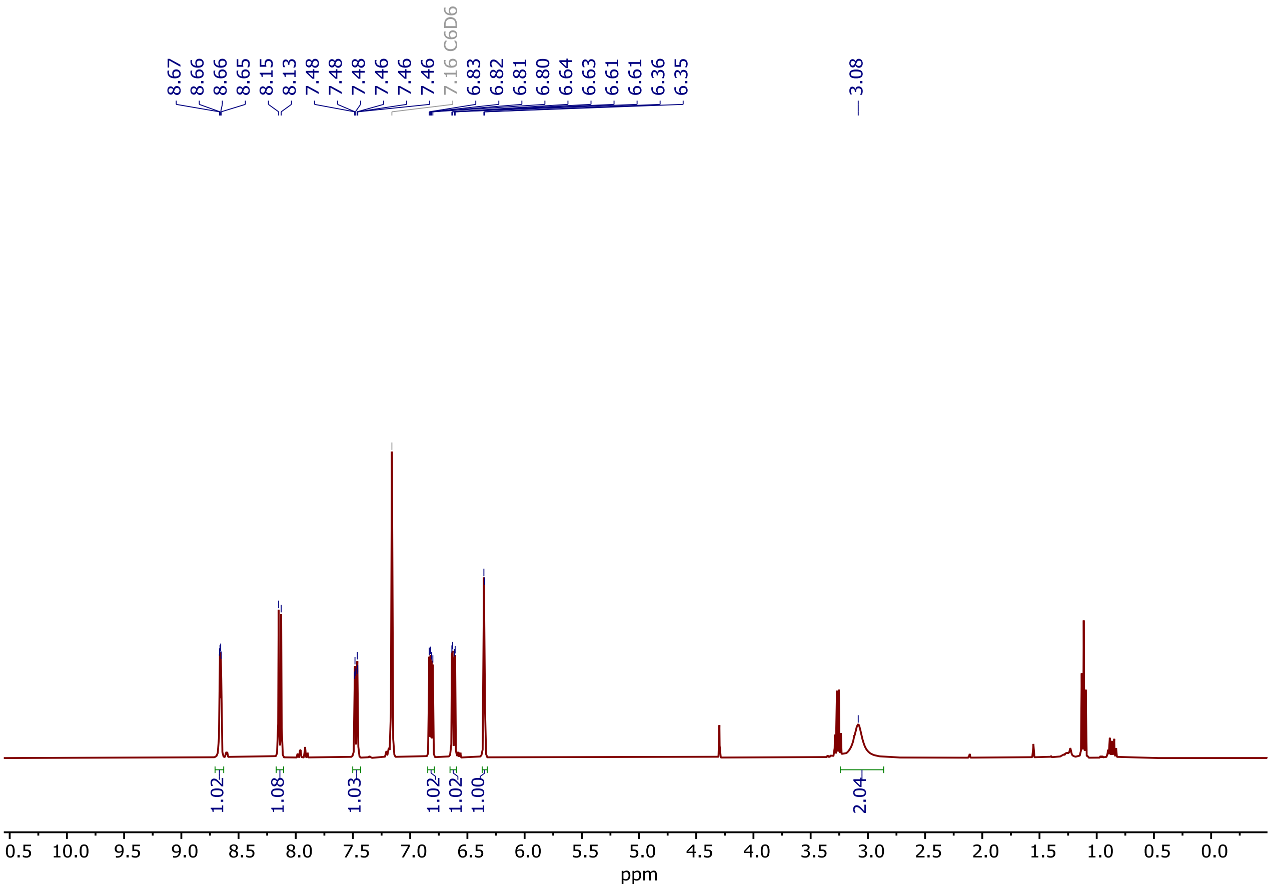

Figure S 60: ^{1}H-NMR spectrum of 6-aminoquinoline in benzene-d_6 at 298 K.

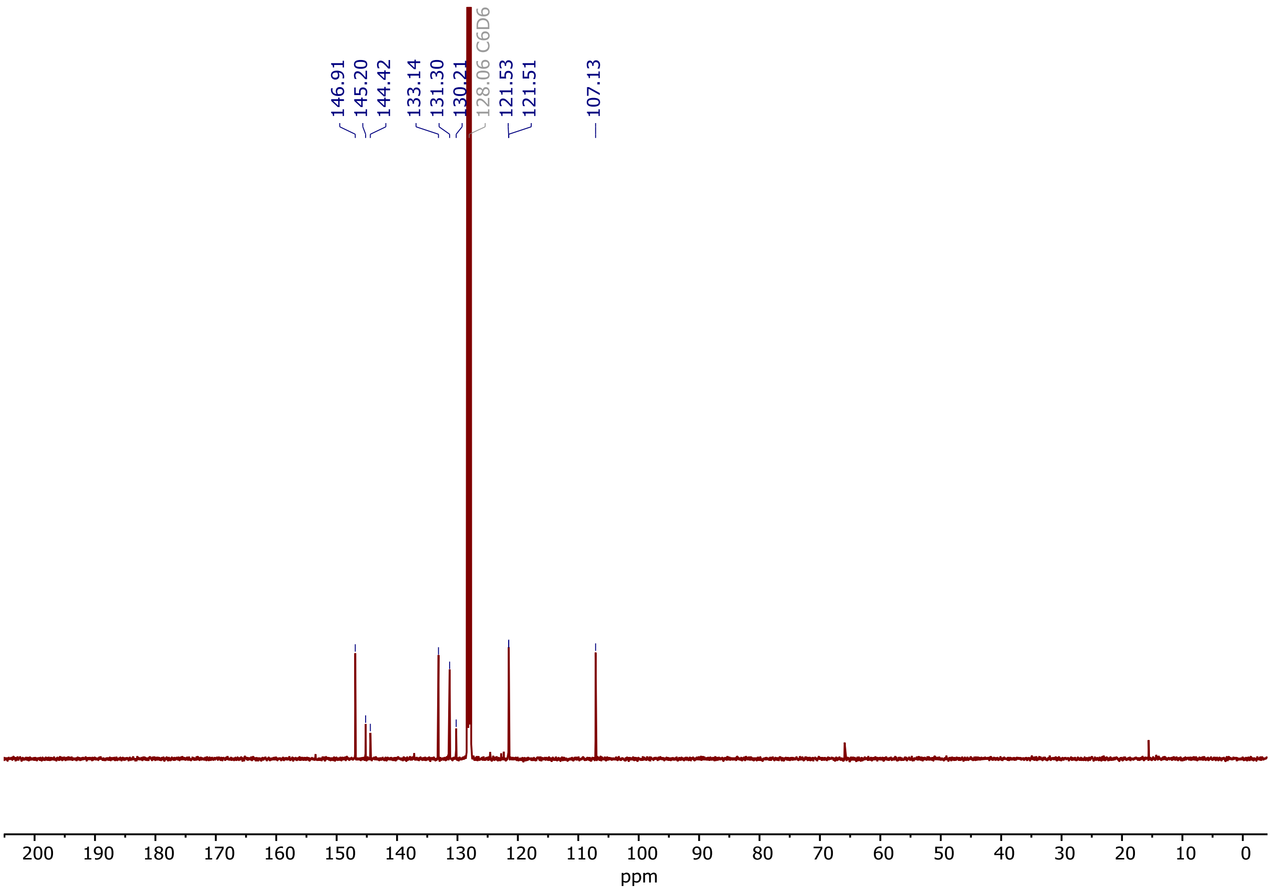

Figure S 61: ^{13}C{^{1}H}-spectrum of 6-aminoquinoline in benzene-d_6 at 298 K.

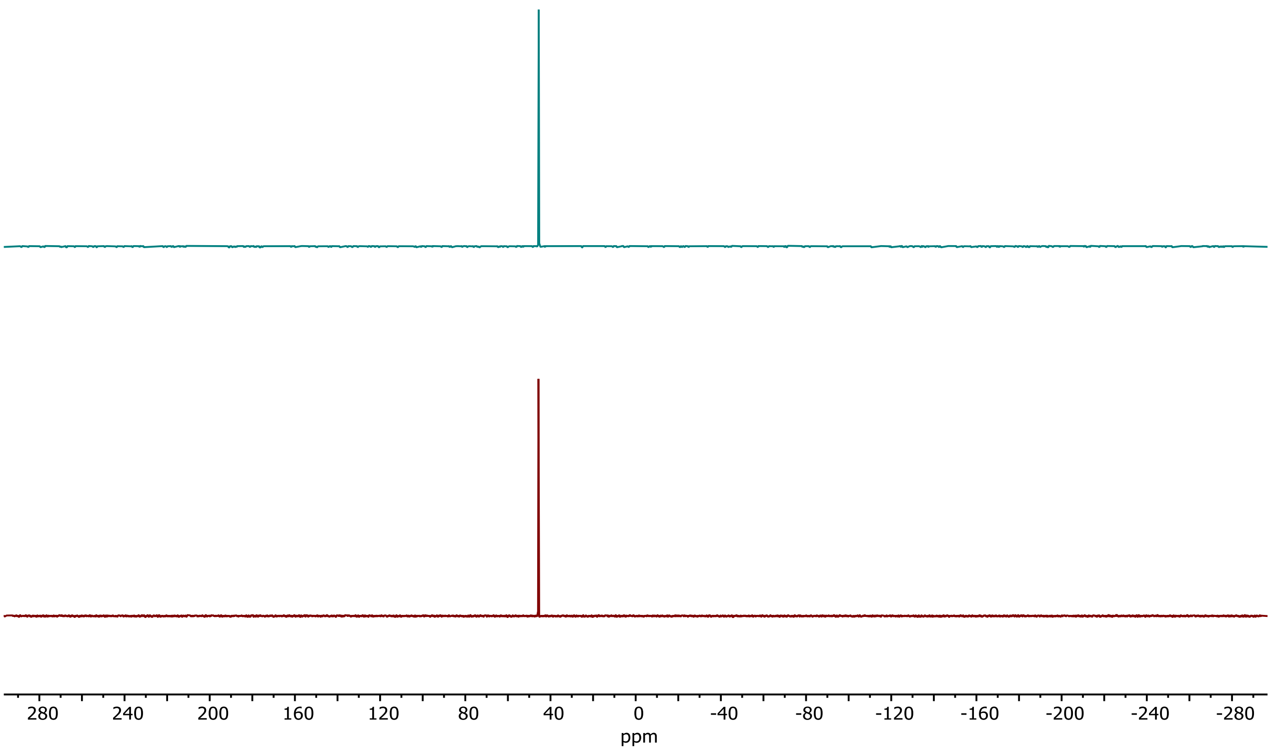

Figure S 62: $^{31}P\{^{1}H\}$-NMR spectrum of a mixture of complex **2-Mo** and triethylphosphine oxide ($OPEt_3$) in benzene-d_6 at 298 K (green), free triethylphosphine oxide in benzene-d_6 at 298 K (red).

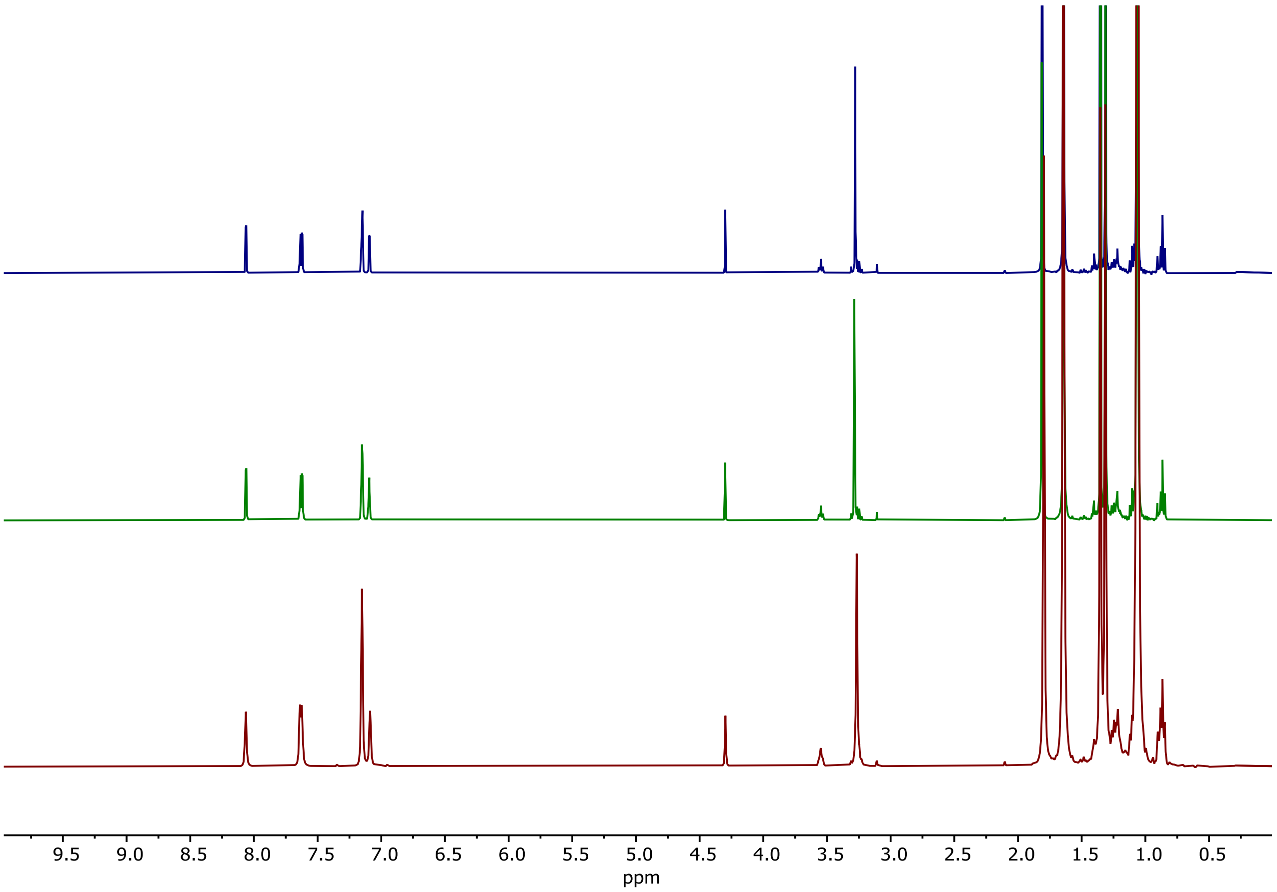

Figure S 63: ^{1}H NMR spectrum of a mixture of **2-Mo** and pinacol (2 equiv.) at room temperature. Red: after mixing, green: after 2 hours, blue: after 4 hours.

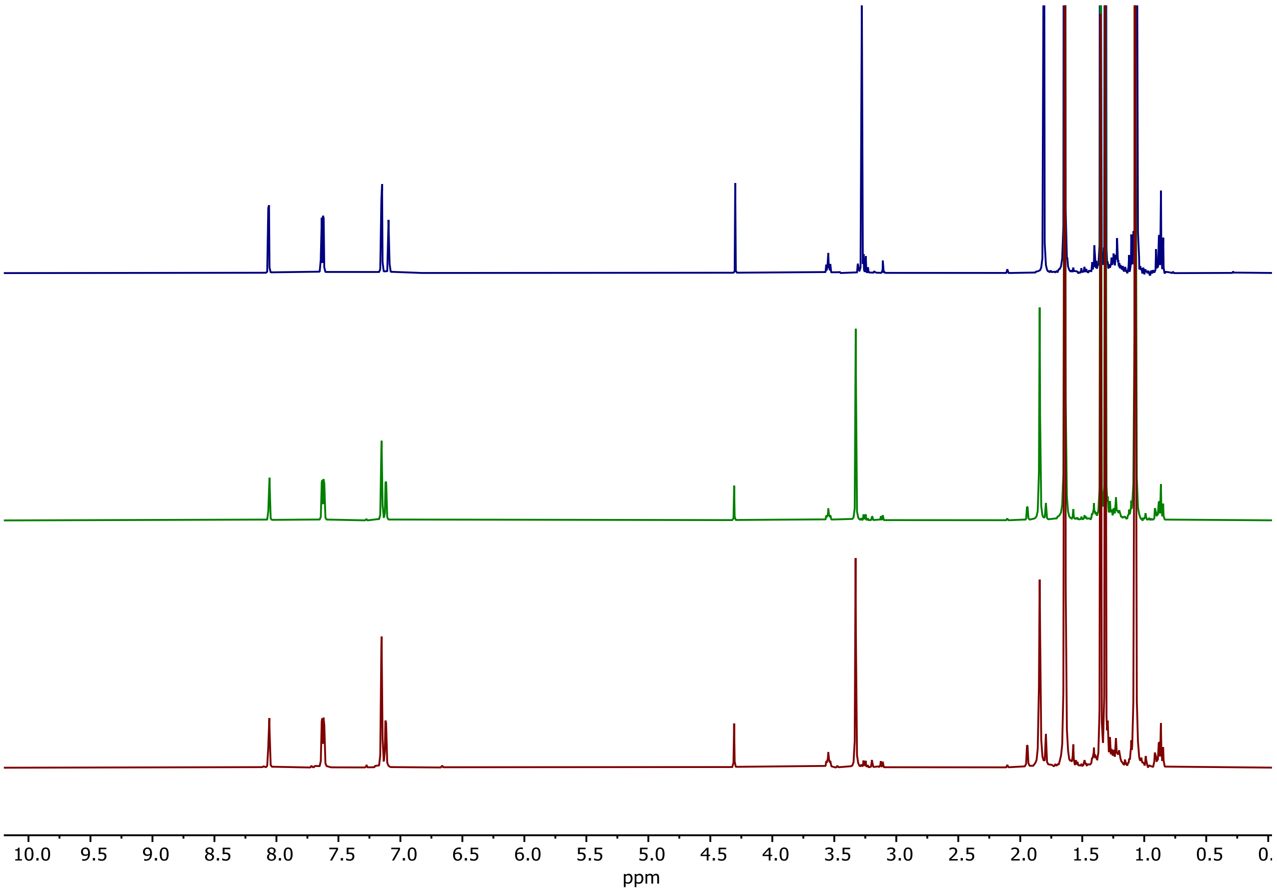

Figure S 64: ^{1}H-NMR spectrum of a mixture of **2-Mo** after heating to 70 °C for 30 minutes, then allowing to cool to room temperature (red: 1st cycle, green: 2nd cycle, blue: 3rd cycle).

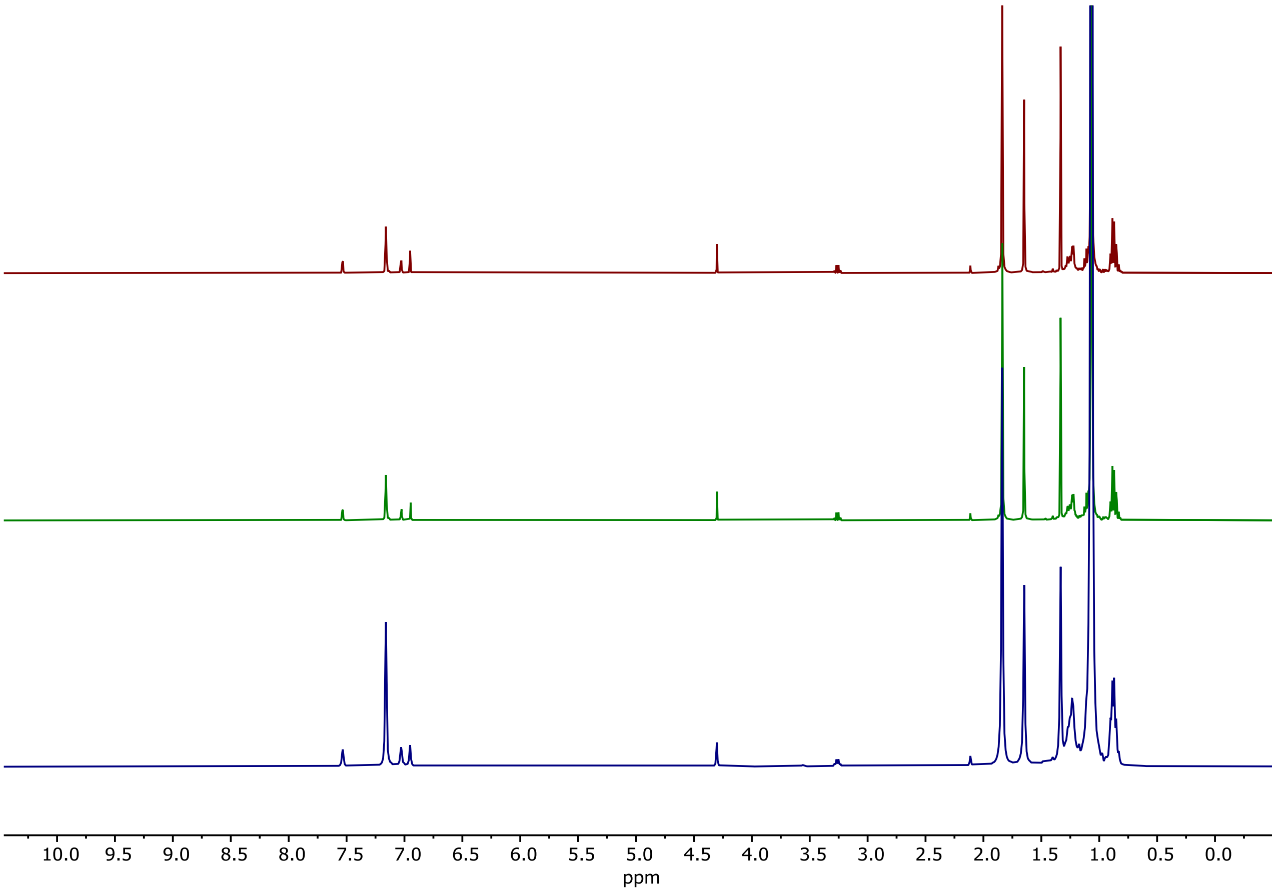

Figure S 65: ^{1}H NMR spectrum of a mixture of **3-Mo** and pinacol (2 equiv.) at room temperature. Red: after mixing, green: after 2 hours, blue: after 4 hours.

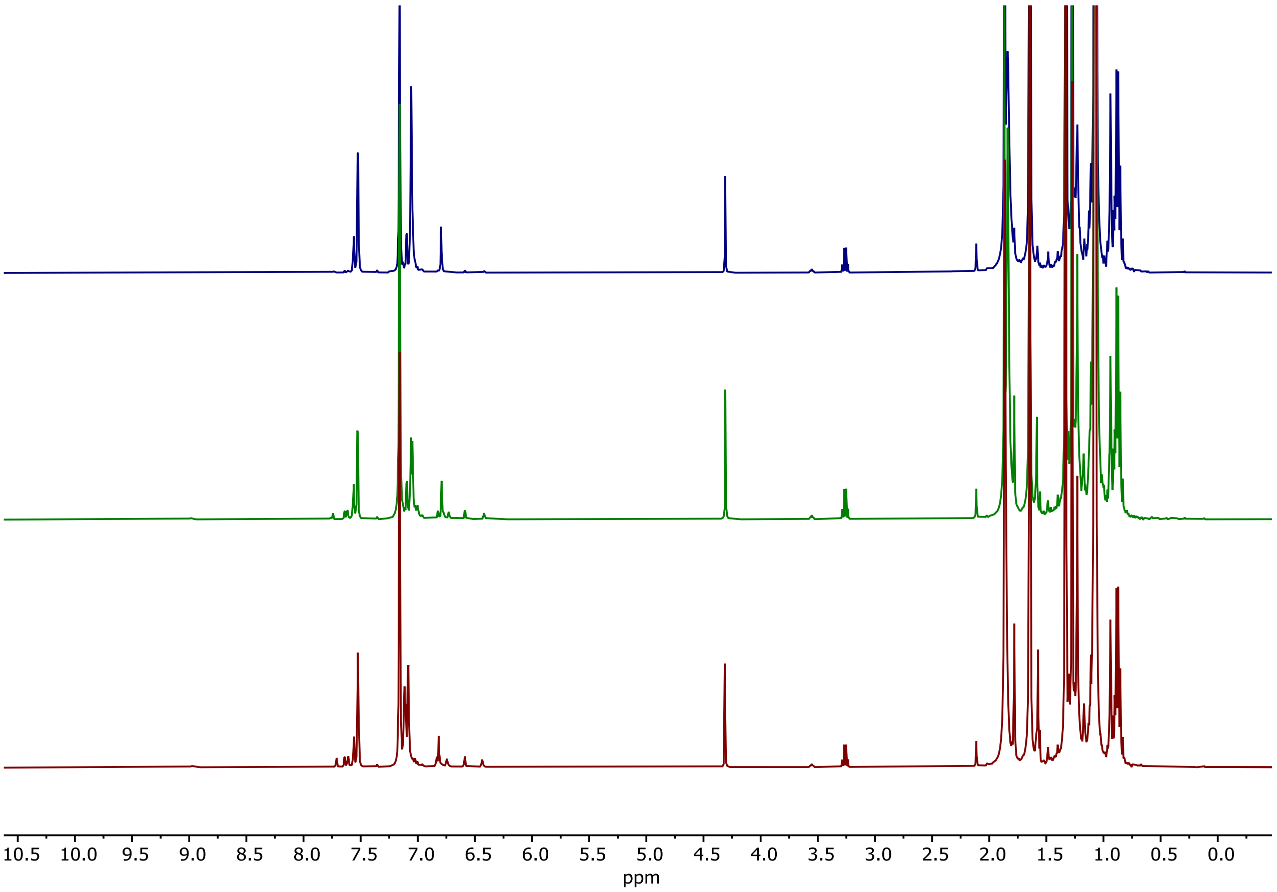

Figure S 66: ^{1}H-NMR spectrum of a mixture of **3-Mo** after heating to 70 °C for 30 minutes, then allowing to cool to room temperature (red: 1st cycle, green: 2nd cycle, blue: 3rd cycle).

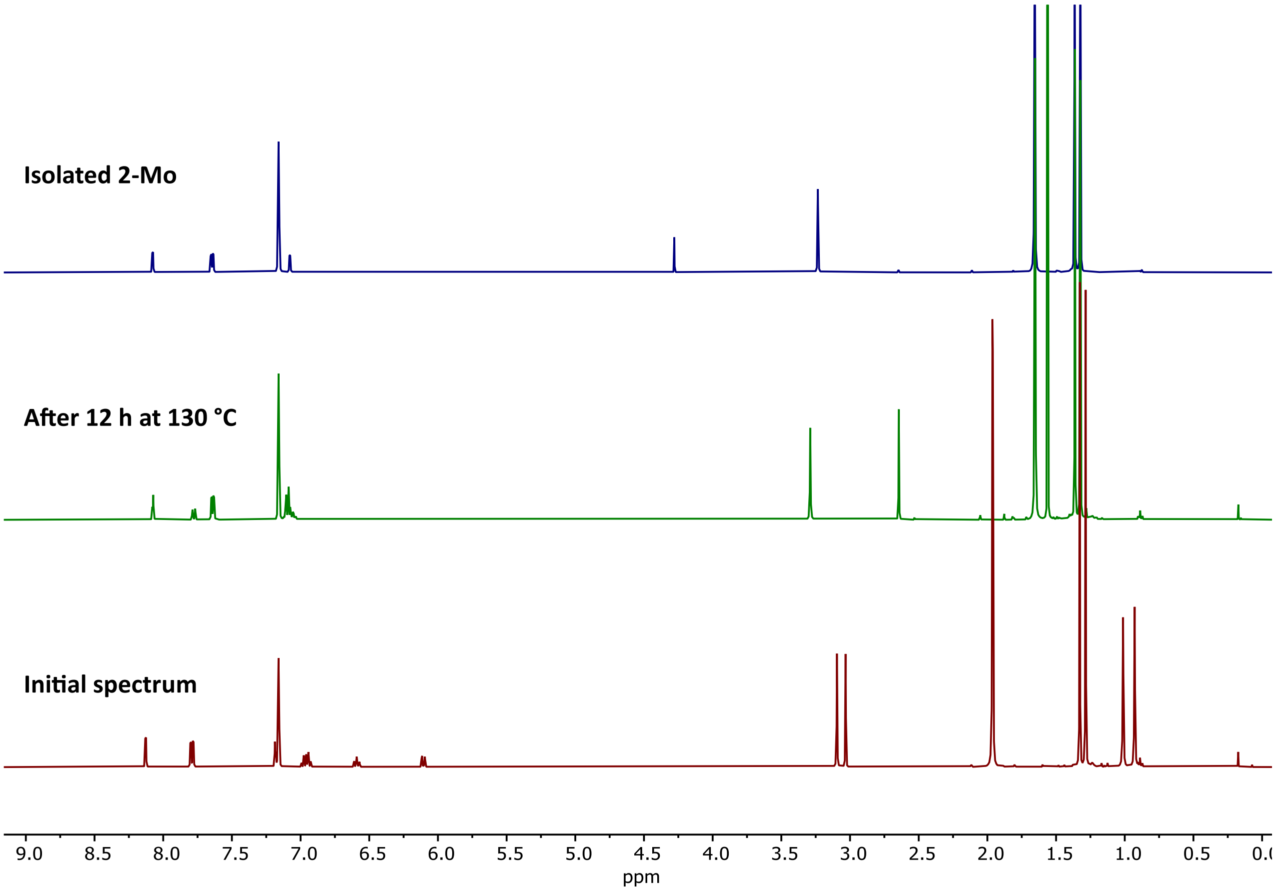

Figure S 67: ^{1}H-NMR spectra of the reaction between pinacolate complex **5-Mo** and *o*-nitrosotoluene at 130 °C.

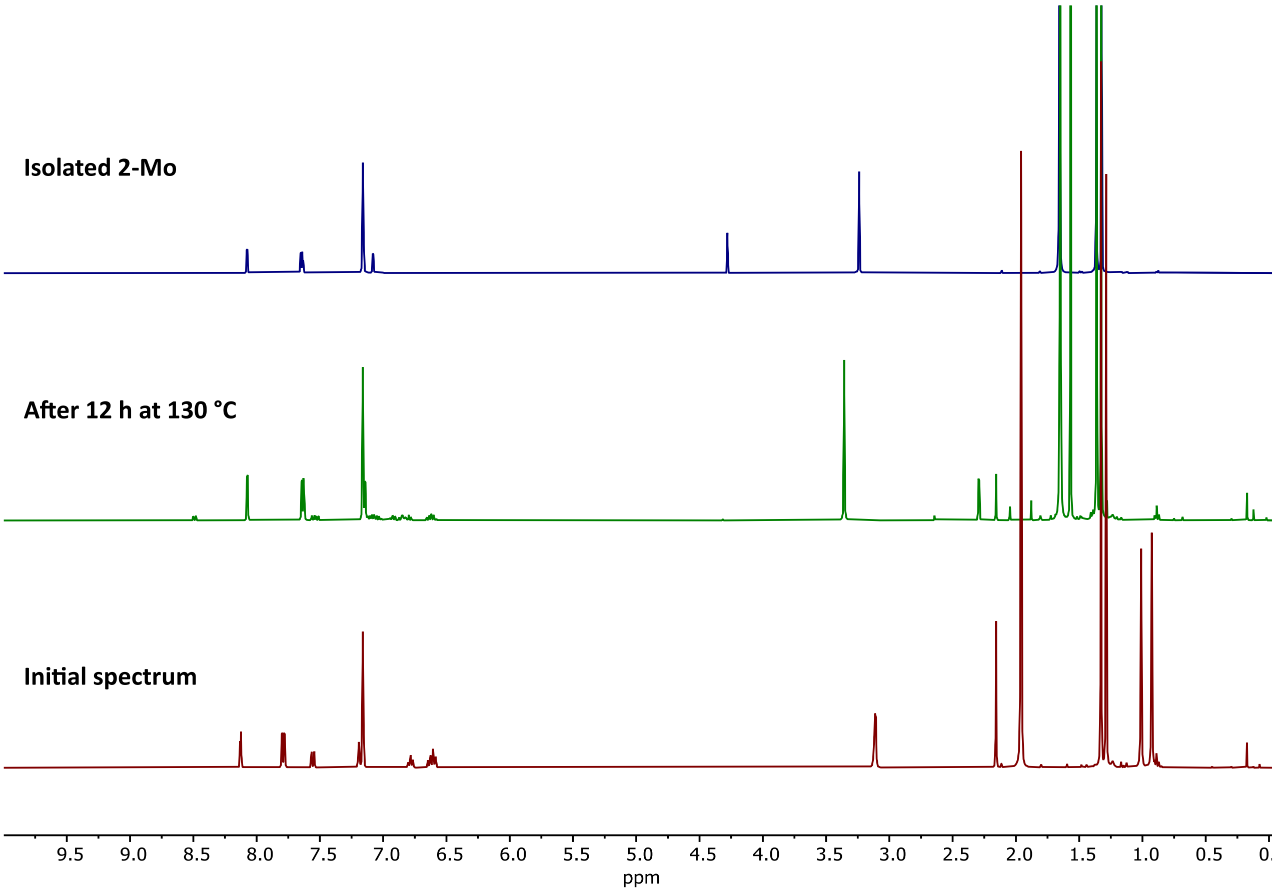

Figure S 68: ^{1}H-NMR spectra of the reaction between pinacolate complex **5-Mo** and *o*-nitrosotoluene at 130 °C.

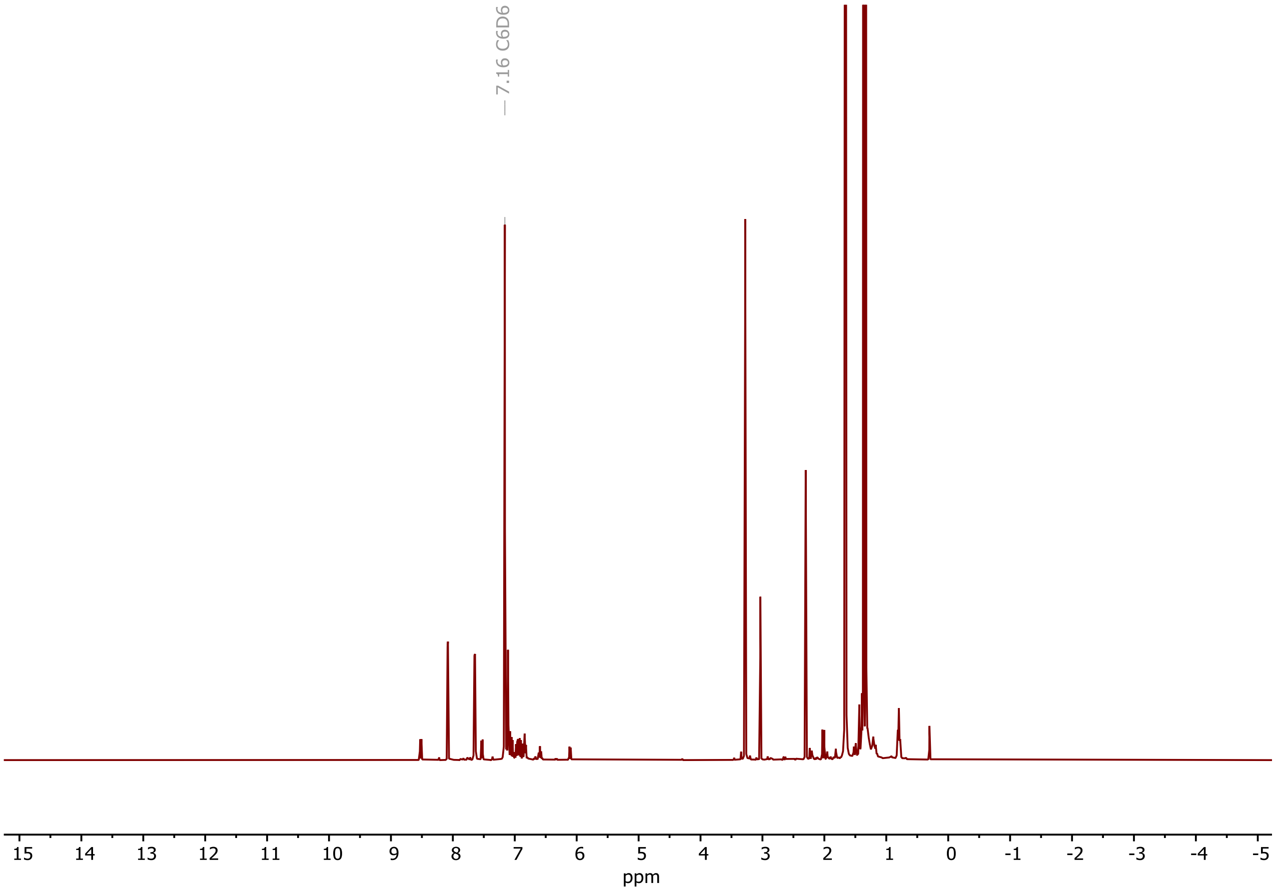

Figure S 69: ^{1}H NMR spectrum of the reaction between **7-Mo** and nitrosotoluene.

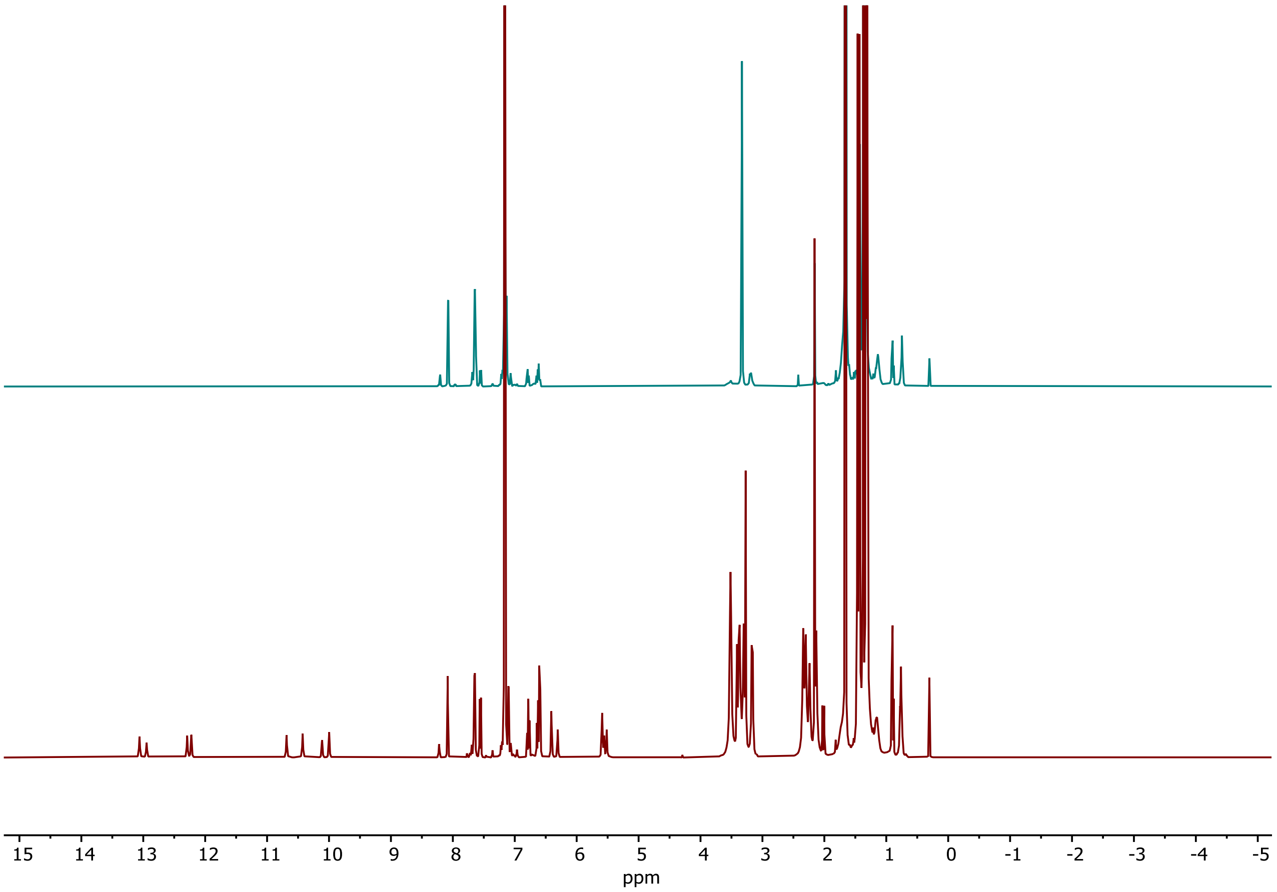

Figure S 70: ^{1}H NMR spectrum of the reaction between **7-Mo** and nitrotoluene at room temperature. The top spectrum was taken after 12 hours, the bottom spectrum 15 minutes after mixing the reagents.

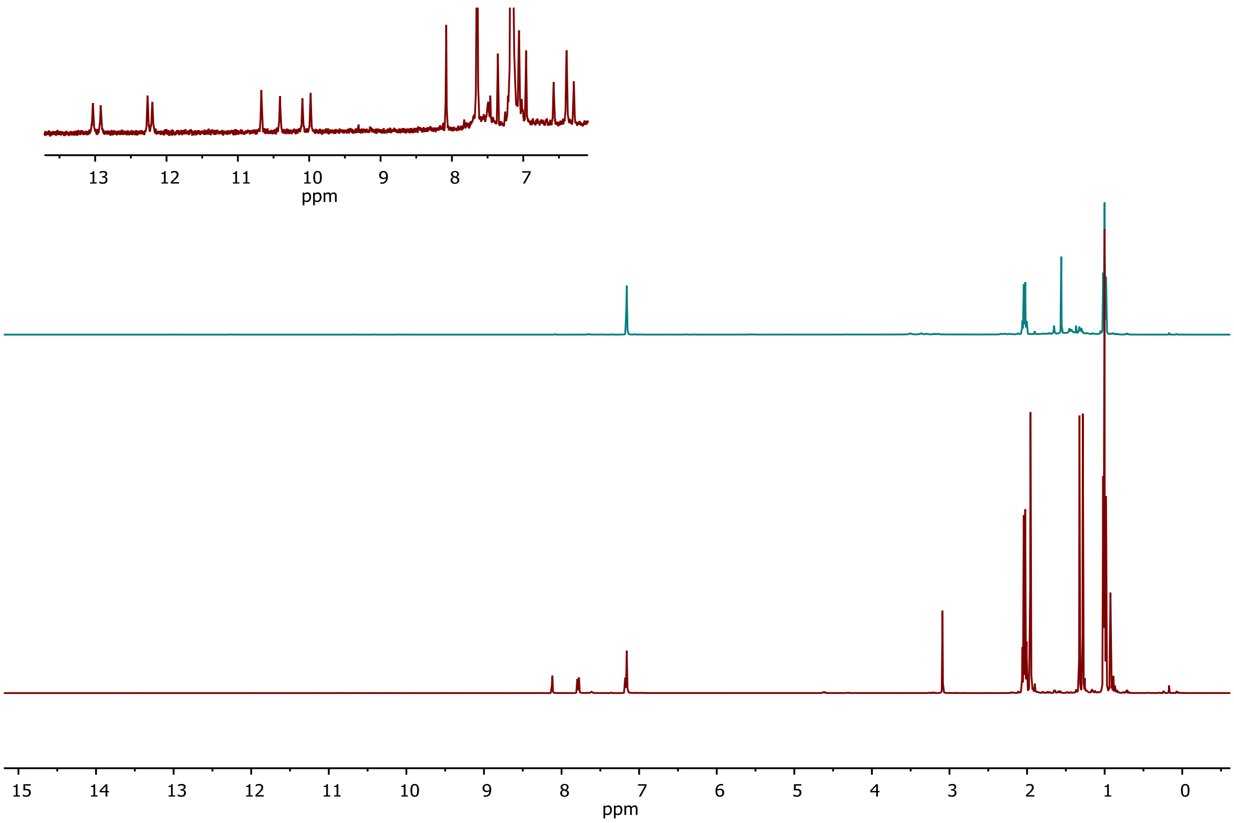

Figure S 71: Mixture of **5-Mo** and 3-hexyne at room temperature (bottom) and after heating for 30 minutes at 130 °C. The resonances of **5-Mo** in the aromatic region clearly disappear and the inlet shows a magnification of the aromatic region after the reaction, displaying the characteristic signals of **7-Mo**. Compare also with Figure S26.

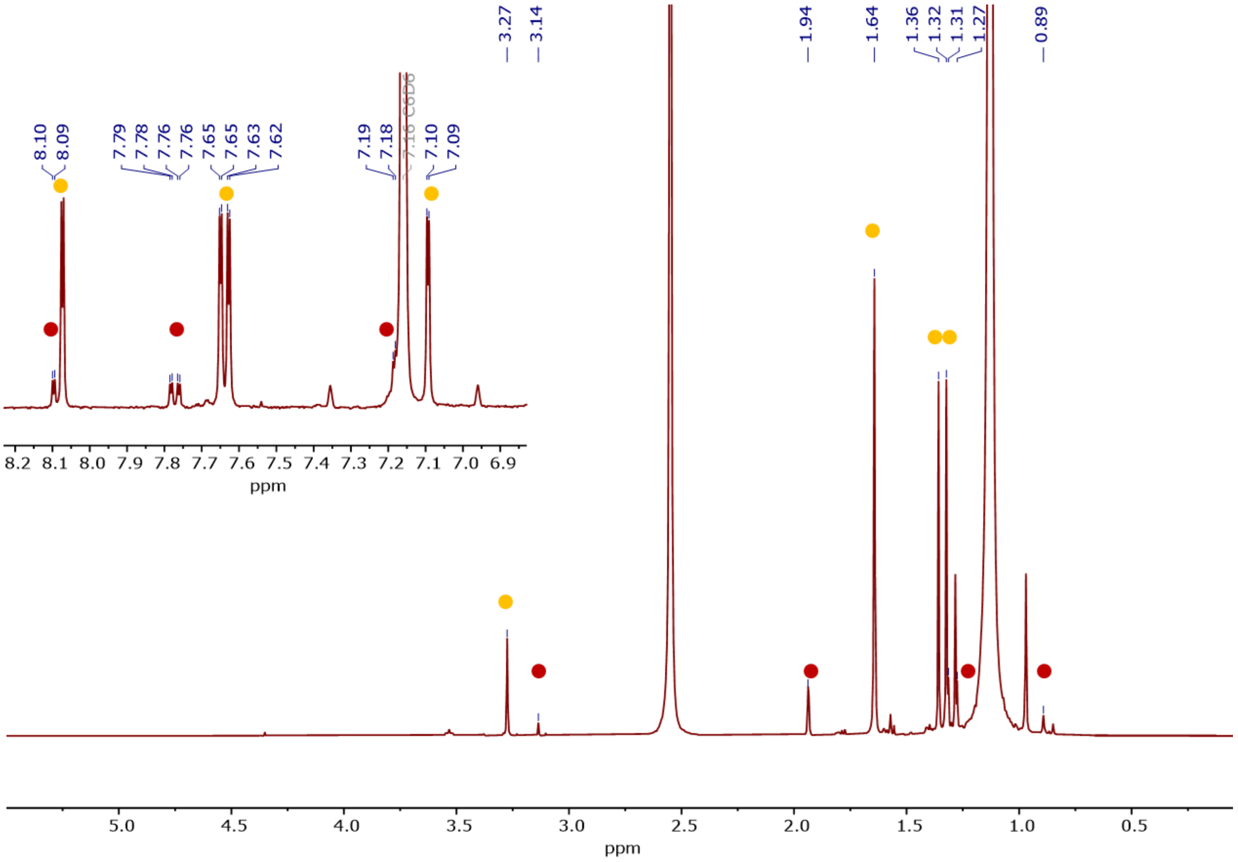

Figure S 72 ^{1}H-NMR spectrum of a mixture of complex **2-Mo** and excess pinacol (63 equiv.), heated to 70 °C and slowly cooled to room temperature. Red dots correspond to oxomolybdenum complex **5-Mo**, yellow dots correspond to the parent dioxomolybdenum complex **2-Mo**.

6. IR spectroscopy

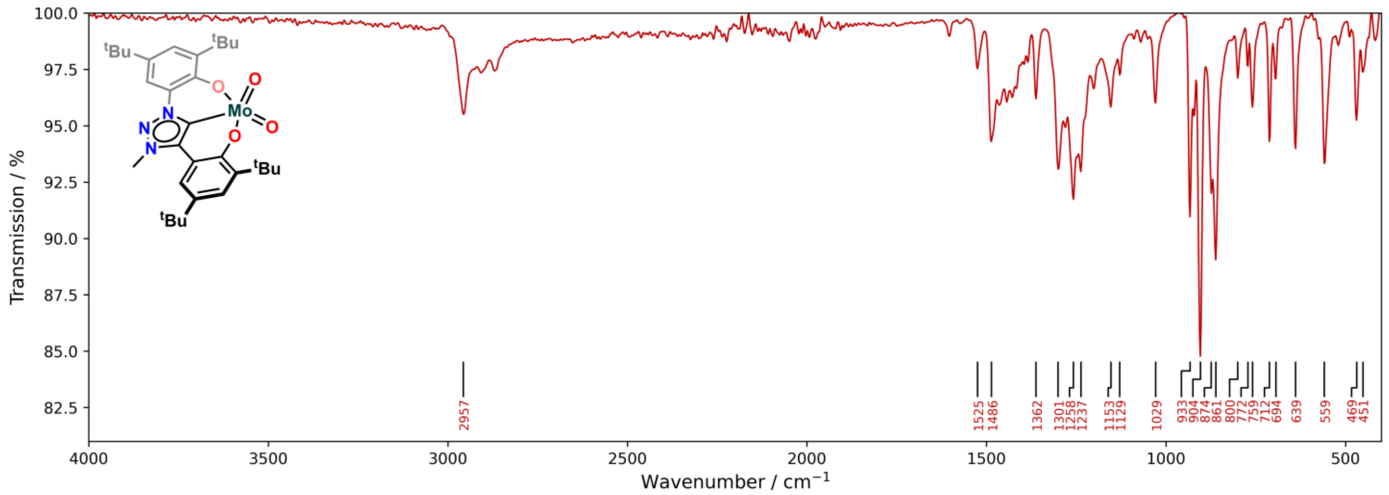

Figure S 73: ATR-IR spectrum of **2-Mo** at 298 K.

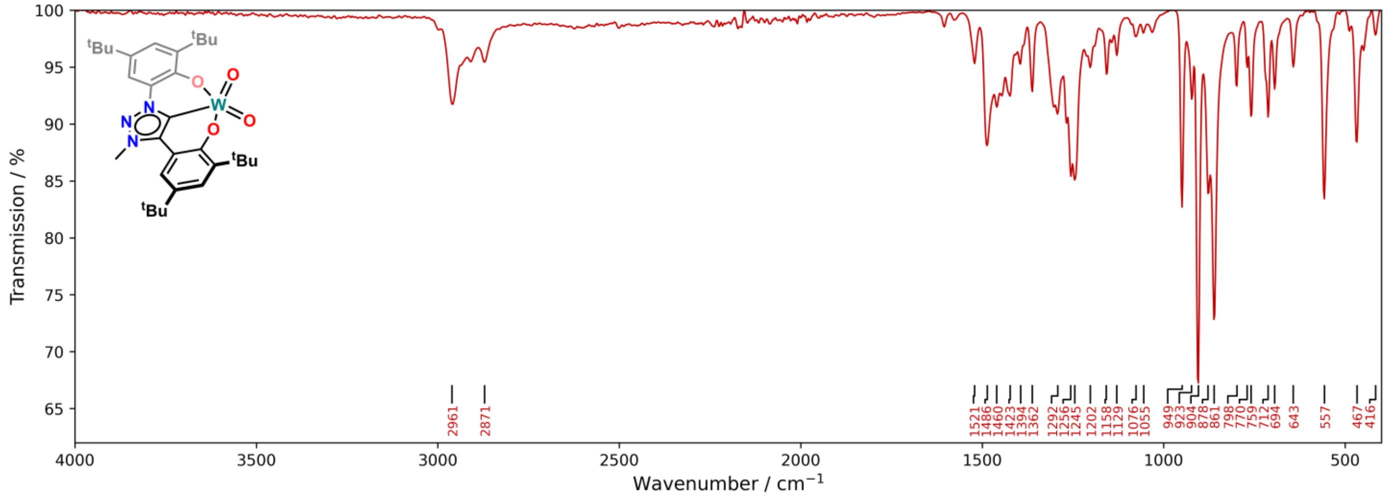

Figure S 74: ATR-IR spectrum of **2-W** at 298 K.

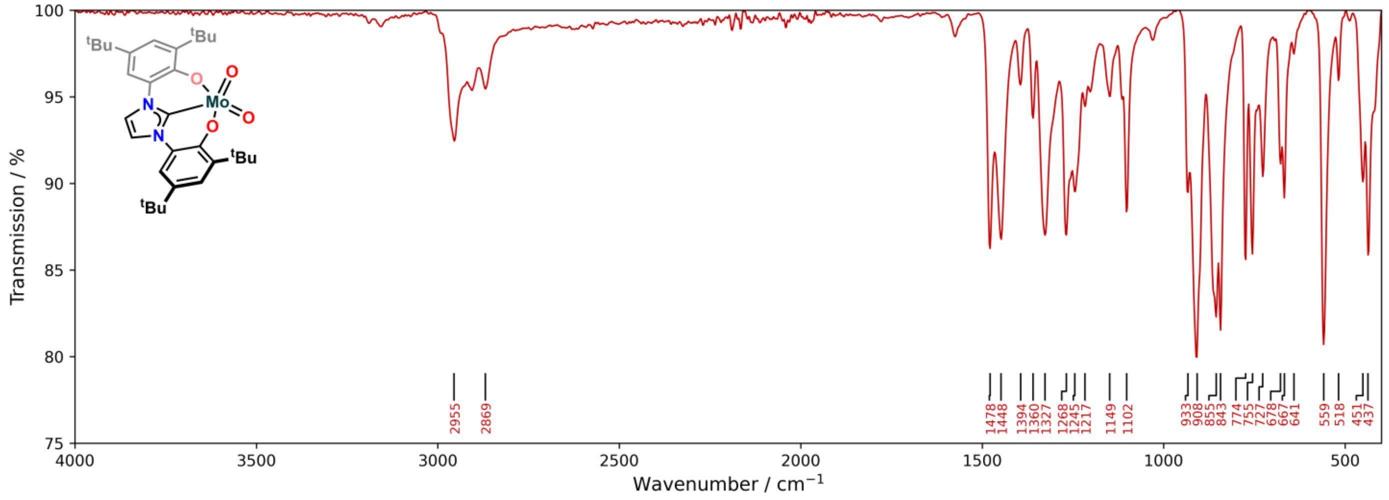

Figure S 75: ATR-IR spectrum of **3-Mo** at 298 K.

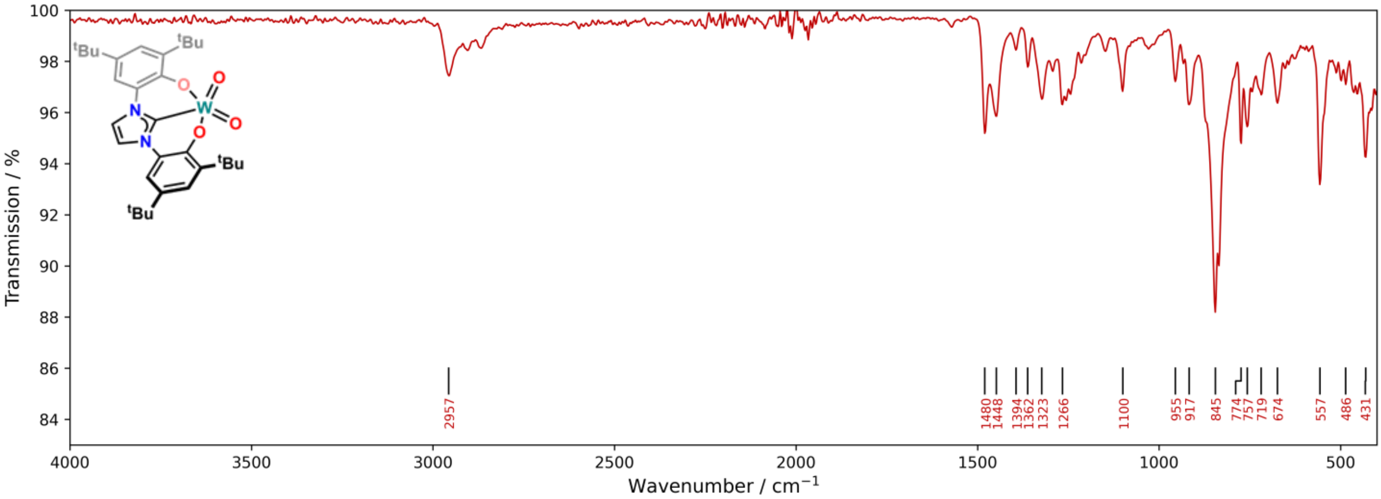

Figure S 76: ATR-IR spectrum of **3-W** at 298 K.

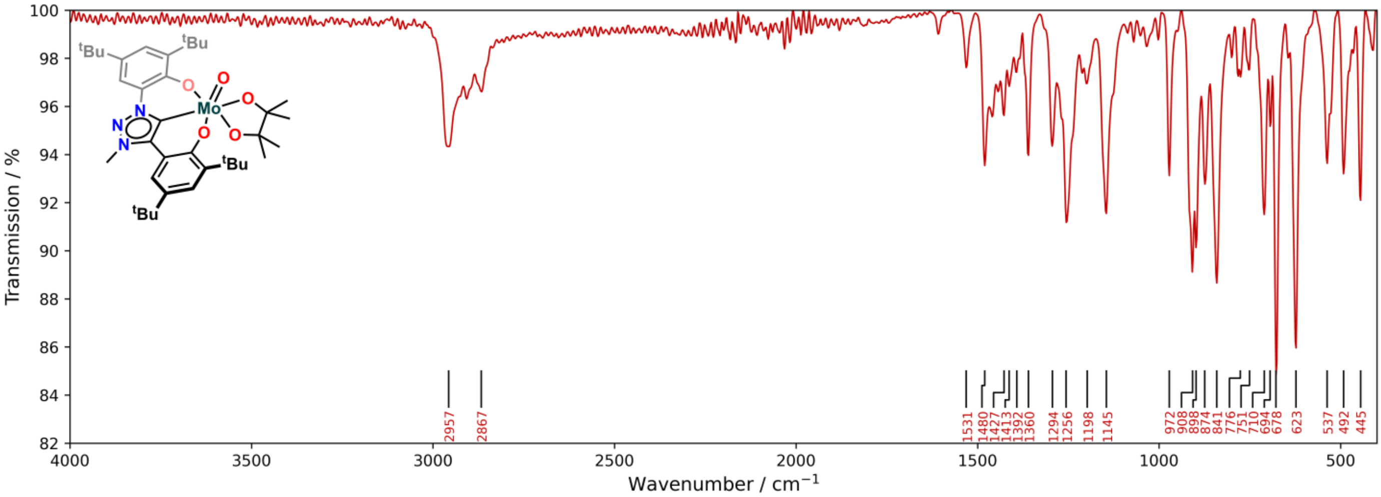

Figure S 77: ATR-IR spectrum of **5-Mo** at 298 K.

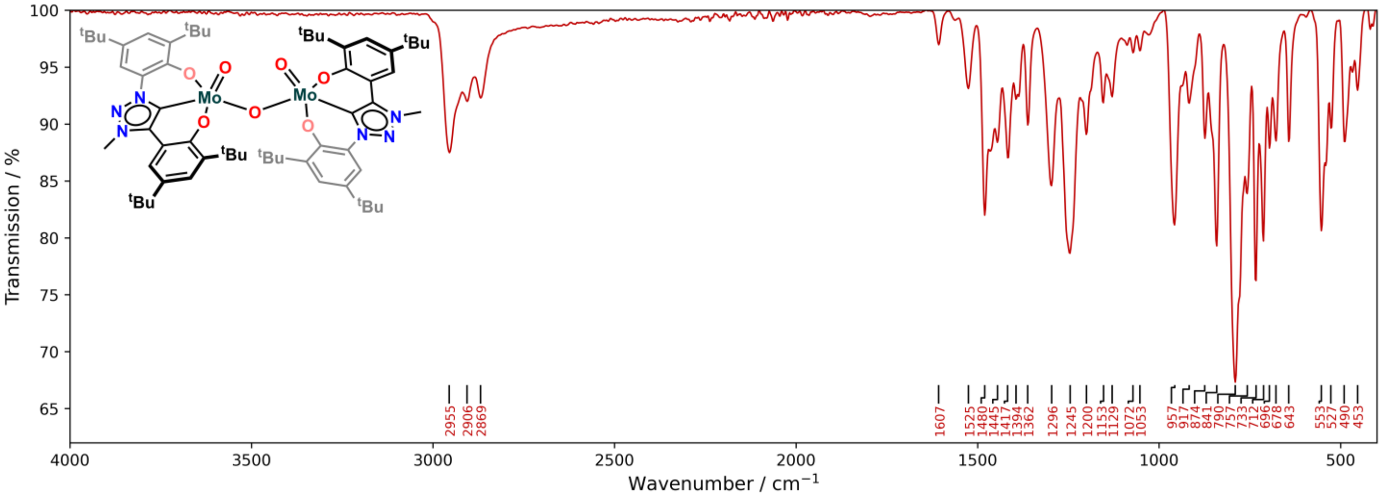

Figure S 78: ATR-IR spectrum of **7-Mo** at 298 K.

7. UV-Vis-NIR spectra

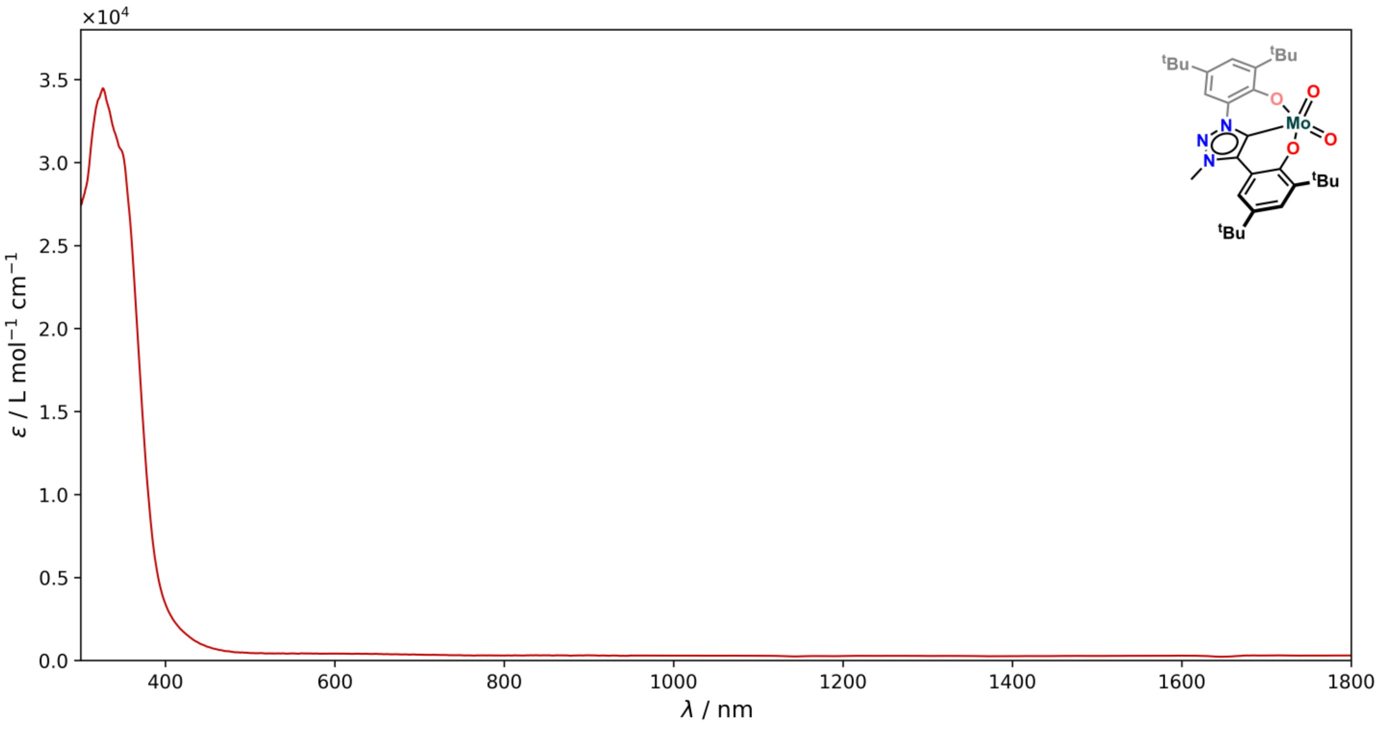

Figure S 79: UV-Vis-NIR spectrum of **2-Mo** in toluene at 298 K.

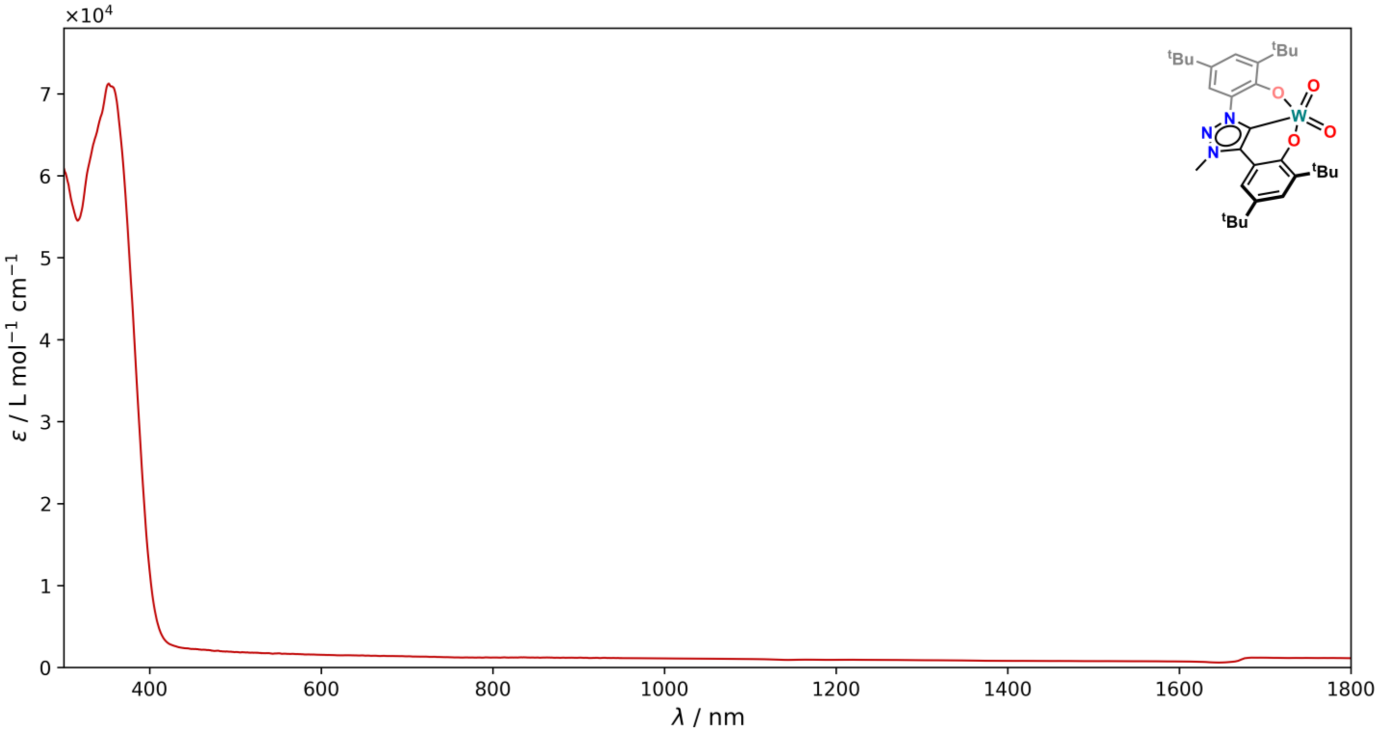

Figure S 80: UV-Vis-NIR spectrum of **2-W** in toluene at 298 K.

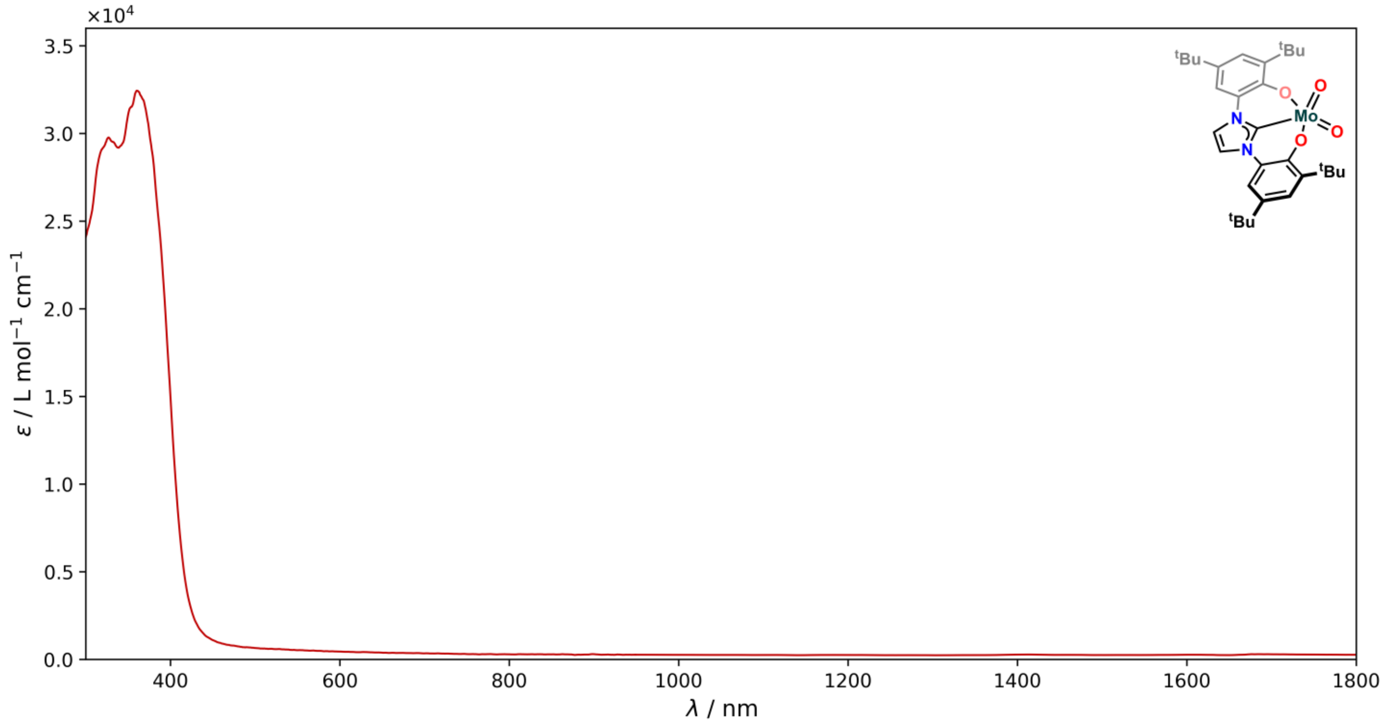

Figure S 81: UV-Vis-NIR spectrum of **3-Mo** in toluene at 298 K.

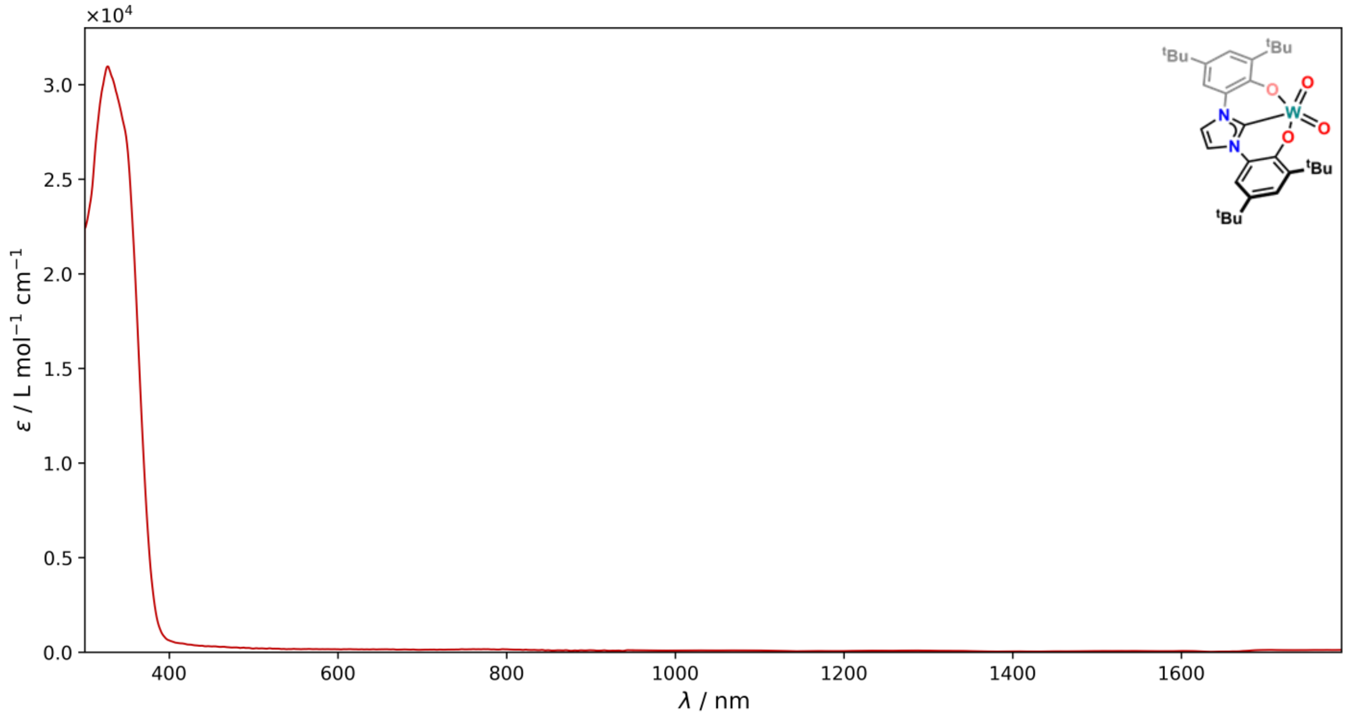

Figure S 82: UV-Vis-NIR spectrum of **3-W** in toluene at 298 K.

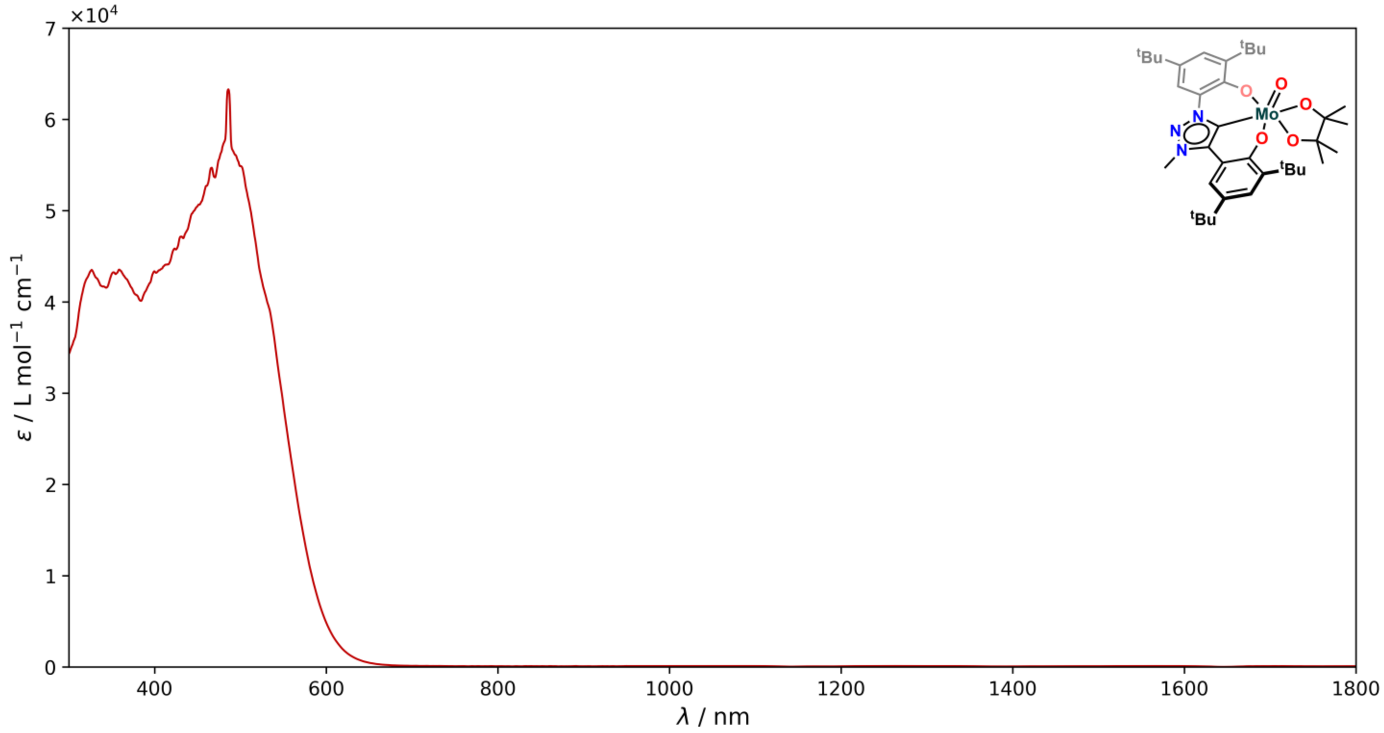

Figure S 83: UV-Vis-NIR spectrum of **5-Mo** in toluene at 298 K.

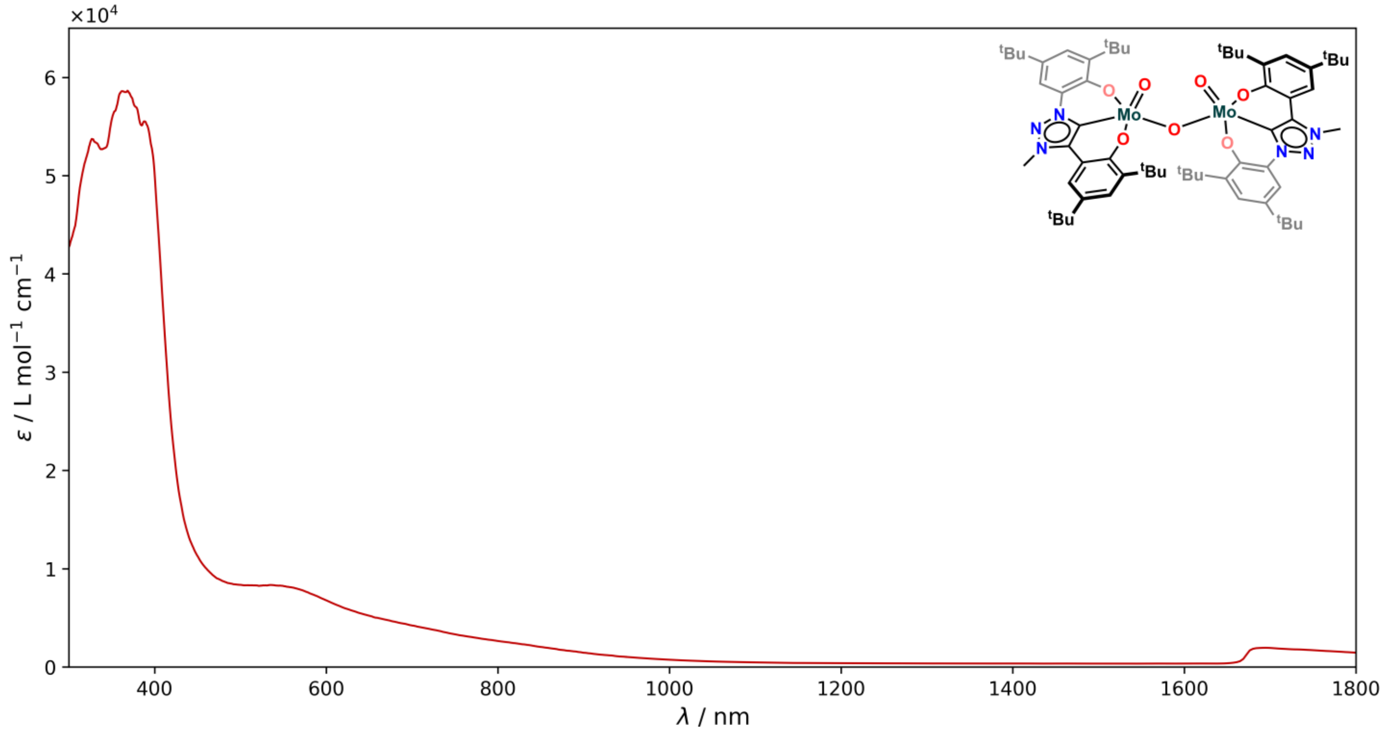

Figure S 84: UV-Vis-NIR spectrum of **7-Mo** in toluene at 298 K.

8. Crystallographic details

Table S 1: Crystallographic details of complexes **2-M** and **3-M** as well as **5-Mo** and **7-Mo** (M = Mo, W)

	**2-Mo**	**2-Mo OPEt₃**	**2-W**	**3-Mo**	**3-W***	**5-Mo**	**7-Mo**
Chemical Formula	$2(C_{31}H_{43}MoN_3O_4)$ C_6H_6	$C_{31}H_{43}N_3O_4Mo$ $C_6H_{15}O_1P_1$	$C_{31}H_{43}N_3O_4W$ C_2H_3N	$C_{31}H_{42}N_2O_4Mo$ 0.5 $C_4H_{10}O$	$C_{353}N_{58}O_{40}W_{12}$	$C_{37}H_{55}MoN_3O_5$	$C_{62}H_{86}Mo_2N_6O_7$
M_r (g mol^{-1})	1313.35	751.77	746.59	639.66	-	717.78	1219.24
Crystal System	*monoclinic*	*Triclinic*	*monoclinic*	*monoclinic*	*triclinic*	*orthorhombic*	*triclinic*
Space Group	$P2_1/c$	$P\bar{1}$	$C2/c$	$P2/c$	$P\bar{1}$	$P2_12_12_1$	$P\bar{1}$
a (Å)	20.485(2)	10.6170(5)	38.186(3)	15.096(3)	31.0457(3)	11.8262(5)	9.9865(6)
b (Å)	19.3444(16)	13.8712(6)	22.3562(14)	15.209(3)	32.1434(3)	15.2730(7)	14.1189(6)
c (Å)	18.6651(16)	15.5294(7)	31.987(2)	43.452(9)	32.2267(3)	20.7099(10)	23.4098(13)
α (°)	90	71.931(2)	90	90	60.396(2)	90	88.577(2)
β (°)	113.992(2)	72.913(2)	105.086(2)	90.09(3)	65.563(2)	90	87.967(2)
γ (°)	90	68.834(2)	90	90	62.823(2)	90	77.070(2)
V (Å^3)	6757.4(11)	1984.45(16)	26367(3)	9977(3)	-	3740.7(3)	3214.5(3)
Z	4	2	32	12	-	4	2
Density (g cm^{-3})	1.291	1.258	1.505	1.278	-	1.275	1.260
F(000)	2760	796	12096	4044	-	1520	1280
Radiation Type	Mo$K\alpha$	Mo$K\alpha$	Mo$K\alpha$	Mo$K\alpha$	Cu$K\alpha$	Mo$K\alpha$	Mo$K\alpha$
μ (mm^{-1})	0.427	0.413	3.546	0.432	-	0.394	0.442
Crystal Size (mm)	0.05 x 0.04 x 0.01	0.15x0.02x0.01	0.15 x 0.14 x 0.05	0.15 x 0.14 x 0.12	0.05x0.04x0.01	0.17 x 0.04 x 0.02	0.13 x 0.08 x 0.07
Meas. Refl.	101298	98059	268245	98369	-	26506	129772
Indep. Refl.	11883	8152	25924	20432	-	6816	13221
Obsvd. $[I > 2\sigma(I)]$	8165	7245	17926	16141	-	6340	9592
R_{int}	0.1277	0.0906	0.0801	0.0487	-	0.0641	0.1312
R [$F^2 > 2\sigma(F^2)$]	0.0517	0.0495	0.0448	0.0438	-	0.0812	0.0528
wR(F^2)	0.1246	0.0957	0.1112	0.0990	-	0.2138	0.1521
S	1.075	1.129	1.101	1.029	-	1.130	1.071
$\Delta\rho_{max}$	1.178	0.387	2.774	1.212	-	4.740	1.248
$\Delta\rho_{min}$	-0.738	-0.879	-1.256	-0.496	-	-1.892	-0.830
CCDC	2383974	2383970	2383972	2383971	-	2383969	2383973

*Due to the low crystal quality, only selected values are given.

Table S 2: Selected bond lengths and angles

	2-Mo	2-Mo OPEt3	2-W	3-Mo	5-Mo	7-Mo
M1-C1	2.170(5)	2.177(3)	2.209(6)	2.225(3)	2.187(12)	2.123(4)
M1-O10	1.696(3)	1.710(2)	1.729(5)	1.713(2)	1.699(8)	1.890(3)
M1-O11	1.705(3)	1.698(2)	1.713(5)	1.691(2)	-	1.656(3)
M1-O1	1.938(3)	1.926(2)	1.924(5)	1.915(2)	2.013(9)	1.968(3)
M1-O2	1.927(3)	1.942(2)	1.922(5)	1.905(2)	2.044(8)	1.989(3)
M1-O40	-	-	-	-	1.910(9)	-
M1-O41	-	-	-	-	1.911(8)	-
O1-M1-O2	155.93(13)	155.87(8)	156.23(19)	156.96(9)	123.4(3)	152.51(14)
O10-M1-O11	112.32(18)	113.65(14)	111.0(3)	112.12(11)	-	116.95(14)
C1-M1-O10	124.24(18)	125.51(12)	128.7(3)	122.56(11)	140.8(4)	129.91(13)
C1-M1-O11	123.43(18)	120.84(12)	120.2(3)	125.30(11)	-	113.06(15)
Mo1-O11-Mo1A	-	-	-	-	-	162.62(17)
τ_5	0.53	0.51	0.46	0.53	-	0.38

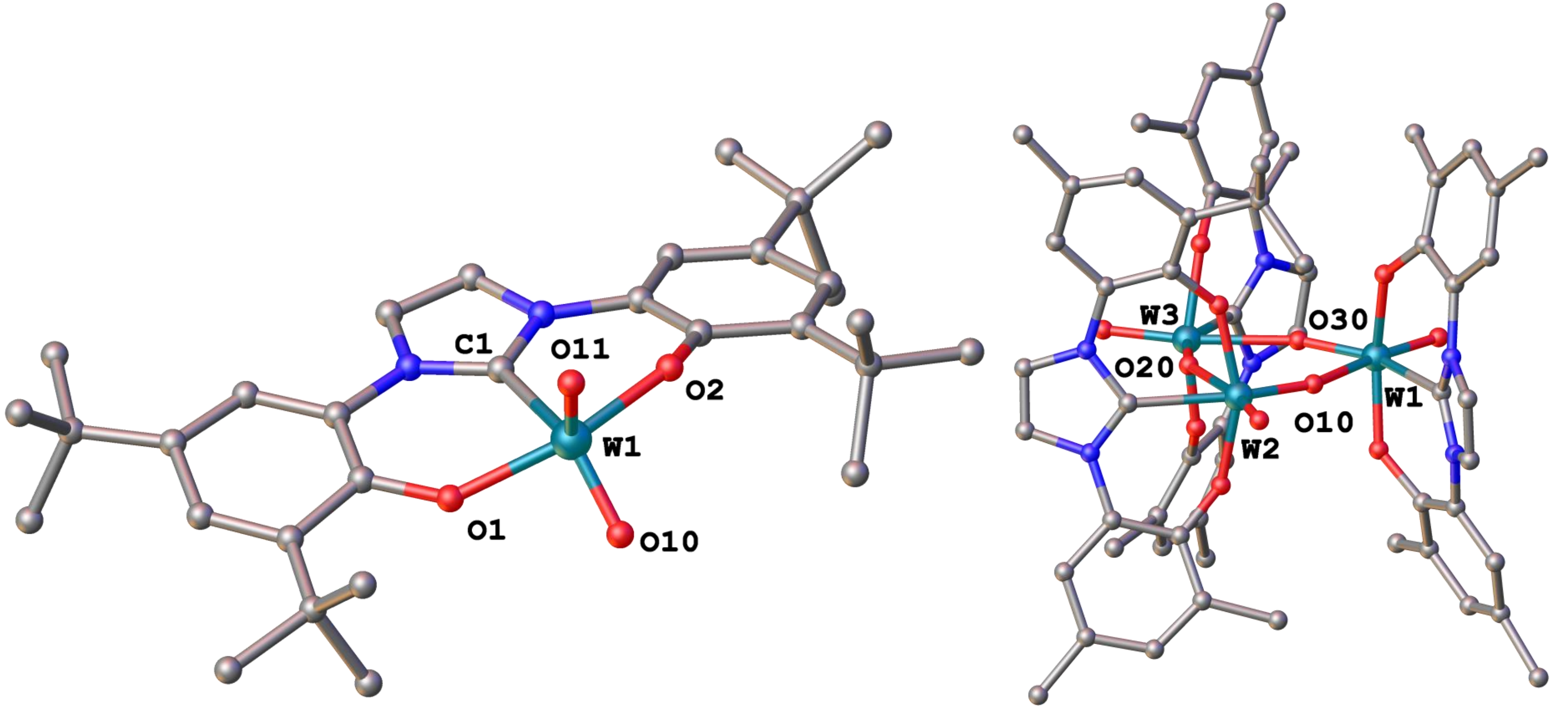

Figure S 85: Molecular structures of the monomeric conformer of **3-W$_{mono}$** (left) and its trimeric form **3-W$_{trimer}$** (right).

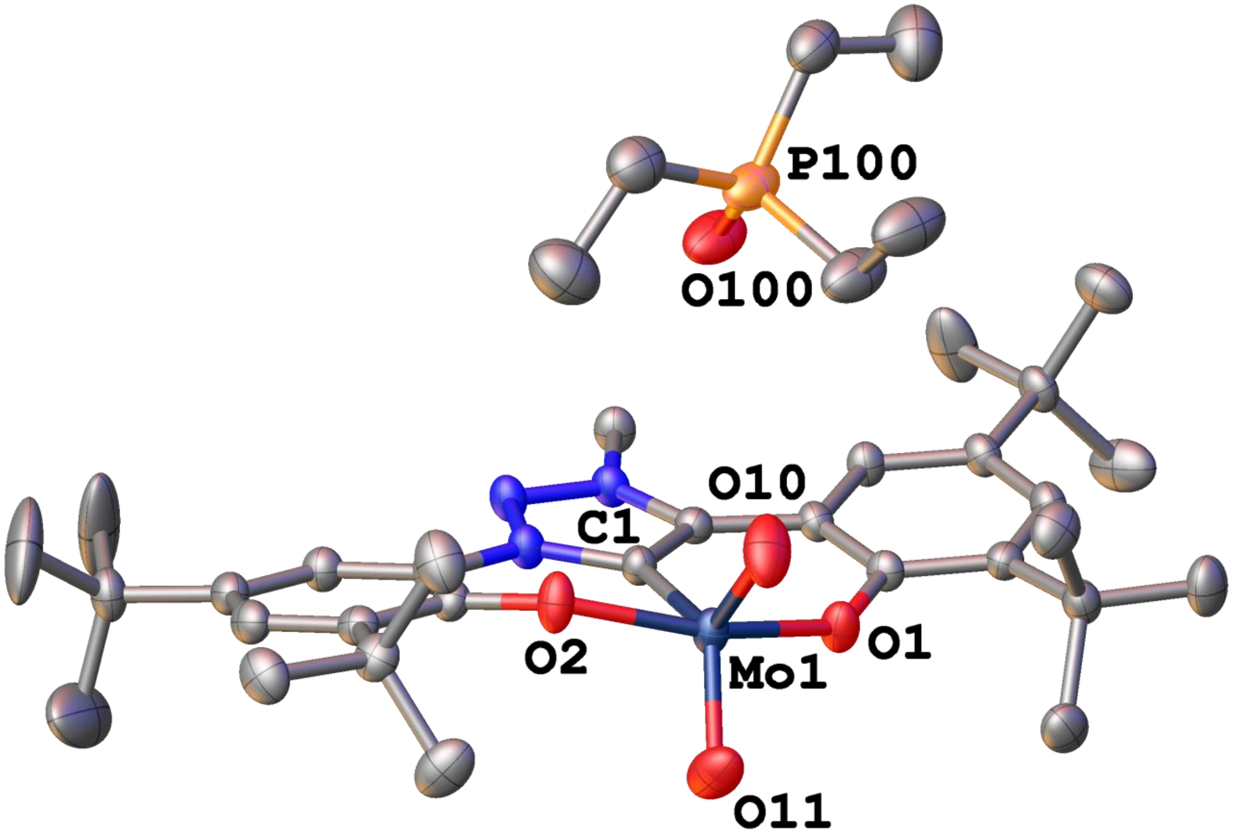

Figure S 86: Molecular structure of **2-Mo OPEt$_3$**, showing that the phosphine oxide donor does not bind to the molybdenum centre in **2-Mo**, contrasting the chemistry of **1-Mo** reported earlier by us. Ellipsoids are shown at a probability level of 50%.[8]

9. Literature

[1] E. Borré, G. Dahm, A. Aliprandi, M. Mauro, S. Dagorne and S. Bellemin-Laponnaz, *Organometallics*, 2014, **33**, 4374–4384.

[2] E. Despagnet-Ayoub, L. M. Henling, J. A. Labinger and J. E. Bercaw, *Dalton Trans.*, 2013, **42**, 15544.

[3] M. Baltrun, F. A. Watt, R. Schoch, C. Wölper, A. G. Neuba and S. Hohloch, *Dalton Trans.*, 2019, **48**, 14611–14625.

[4] M. Baltrun, F. A. Watt, R. Schoch and S. Hohloch, *Organometallics*, 2019, **38**, 3719–3729.

[5] G. M. Sheldrick, *Acta Crystallogr. Sect. A* 2015, **71**, 3–8

[6] O. V. Dolomanov, L. J. Bourhis, R. J. Gildea, J. A. K. Howard and H. Puschmann, *Appl. Crystallogr.,* 2009*,* **42***,* 339–341.

[7] A. L. Spek, *Acta Crystallogr., Sect. A: Found. Crystallogr.*, 2015, **71**, 9–18.

[8] S. Liu, J. I. Amaro-Estrada, M. Baltrun, I. Douair, R. Schoch, L. Maron and S. Hohloch, *Organometallics*, 2021, **40**, 107–118.
